# PLANTA Protocol for the Direct Detection and Identification of Bioactive Compounds in Complex Mixtures via Combined NMR-HPTLC-Based Heterocovariance

**DOI:** 10.1021/acs.analchem.5c02192

**Published:** 2025-10-03

**Authors:** Vaios Amountzias, Evagelos Gikas, Nektarios Aligiannis

**Affiliations:** † Department of Pharmacognosy and Natural Products Chemistry, Faculty of Pharmacy, 68993 National and Kapodistrian University of Athens, Panepistimiopolis Zografou, 15771 Athens, Greece;; ‡ Department of Analytical Chemistry, Faculty of Chemistry, National and Kapodistrian University of Athens, Panepistimiopolis Zografou, 15771 Athens, Greece

## Abstract

The assignment of bioactivity to compounds within complex natural product (NPs) mixtures remains a significant challenge in NPs research. The present research introduces a comprehensive protocol, named “PLANTA (**P**hytochemica**L A**nalysis for **N**a**T**ural bio**A**ctives)” protocol, for the detection and identification of bioactive compounds in complex natural extracts prior to isolation combining the **NMR**-**Het**ero**C**ovariance **A**pproach (NMR-HetCA), high-performance thin-layer chromatography (HPTLC), and chemometric techniques. This study emphasizes two novel components: STOCSY-guided targeted spectral depletion, adapted to resolve overlapping NMR signals in complex matrices, improve minor component detection, and facilitate identification through NMR databases, as well as a new SHY variant termed SH-SCY (**S**tatistical **H**eterocovariance – **S**pectro**C**hromatograph**Y**), a new cross-correlation method linking orthogonal datasets by identifying the corresponding HPTLC spot from a single NMR peak and reconstructing of the ^1^H NMR spectrum from a specific HPTLC spot, enhancing dereplication confidence. In this proof-of-concept study, an artificial extract (ArtExtr) composed of 59 standard compounds was evaluated for the detection of compounds active against the free radical 2,2-diphenyl-1-picrylhydrazyl (DPPH). Statistical approaches were applied to the spectral, chromatographic, and bioactivity data to identify the highly correlated bioactive compounds. The PLANTA protocol achieved an 89.5% detection rate of active metabolites and 73.7% correct identification of them. The integration of NMR and HPTLC with HetCA provides a robust and sensitive strategy for preisolation identification of bioactive constituents. This methodology addresses core challenges in metabolite profiling of complex mixtures and offers a streamlined, reproducible workflow for natural product dereplication and discovery.

## Graphical abstract

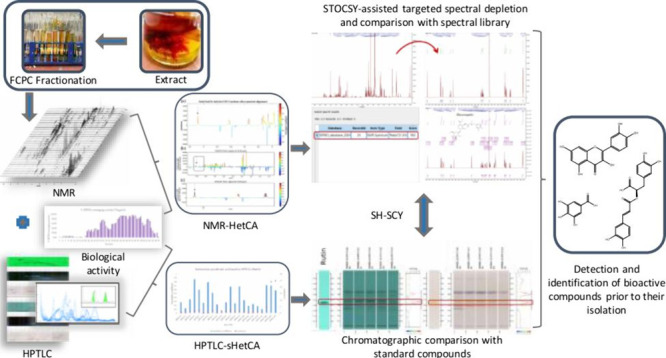

## INTRODUCTION

Pharmacognosy plays a key role in medicine, as many modern pharmaceuticals originate from natural products (NPs). Between 1981 and 2002, over 60% of anticancer drugs and 75% of anti-infective agents were derived from natural sources.^1^ By 2019, 49.5% of all approved drugs were NP-based or NP-inspired.^2,3^ Despite their generally lower toxicity and higher clinical success rates,^4^ the pharmaceutical industry often views NPs as time-consuming, expensive, and high-risk.^5^ Traditional bioassay-guided isolation workflows, comprising extraction, fractionation, bioactivity evaluation, compound isolation, and structural elucidation,^6,7^ frequently lead to the repeated isolation of the same compounds from neighboring fractions. This not only extends experimental timelines but also increases the likelihood of isolating inactive or already known constituents.^5,8^ As a result, there is a growing demand for analytical techniques that enable the rapid identification of secondary metabolites in complex mixtures prior to isolation, thereby streamlining screening and dereplication.^9–12^

Several MS-based workflows have been developed to meet this need, often employing chemometric techniques or molecular networking.^13–16^ However, these methods depend heavily on ionization efficiency^17^ and MS/MS fragmentation libraries, often lack visual traceability, and do not directly facilitate compound isolation. In addition, success rates or false discovery metrics are rarely reported, complicating evaluation.

In contrast, NMR spectroscopy has emerged as a powerful, reproducible, nondestructive, and quantitative tool.^18,19^ Its ability to capture comprehensive chemical profiles without derivatization or ionization makes it particularly well-suited for metabolite analysis. One method developed by our group to extract bioactivity-relevant information from such datasets is the **NMR**-**Het**ero**C**ovariance **A**pproach (NMR-HetCA).^20,21^ It identifies statistically correlated spectral regions across multiple fractions and aligns them with biological activity trends by calculating covariance and Pearson correlation coefficients between ^1^H NMR spectra and corresponding activity data. The output is a pseudospectrum, visually resembling a ^1^H NMR spectrum, where bioactivity-correlated resonances are highlighted. NMR-HetCA builds on foundational concepts from **S**tatistical ****T**O**tal **C**orrelation **S**pectroscop**Y** (STOCSY)^22^ and **S**tatistical **H**eterospectroscop**Y** (SHY),^23^ which assess within- and cross-platform covariance, respectively.^22–24^ These approaches are widely used in metabolomics to resolve overlapping signals and reconstruct pseudospectra of covarying metabolites. Implementation typically involves MATLAB-based scripts, although Borges et al. have introduced a Python-based pipeline (DAFdiscovery) that integrates these algorithms.^25^ NMR-HetCA has demonstrated strong potential in revealing active constituents in natural extracts and has been used by multiple research groups for the early-stage identification of minor bioactive components in combination with STOCSY and/or SHY.^26–30^ Nevertheless, even though these algorithms have been used in multivariate dereplication pipelines, particularly in studies combining NMR and LC–MS data, they are rarely applied in workflows that generate database-compatible spectra. Furthermore, integration with chromatographic correlation for direct compound identification remains uncommon.

On the chromatographic front, HPTLC offers a rapid, robust, high-throughput and low-cost platform for resolving and visualizing complex mixtures.^31^ Recent developments in HPTLC-bioautography have facilitated the rapid detection of bioactive compounds by combining the concurrent separation of multiple samples with effect-directed biological assays.^32,33^ This process typically involves developing two identical plates: one for bioautography and the other for chemical comparison, allowing conclusions to be drawn regarding the chemical nature of bioactive zones. Unlike LC–MS/MS-based methods, HPTLC enables direct visualization of compounds and can be coupled with bioassays, MS or digital quantification using either open-source or commercial software.^34–39^ Although a number of bioautographic methods exist, their availability is still limited relative to standard in vitro bioassays. To address this, our group has developed multivariate chemometric approaches using HPTLC data,^26,40^ and introduced HPTLC-sHetCA (sparse HetCA), a method implemented in Excel (Microsoft Corporation, Redmond, WA, USA), using built-in covariance and correlation functions.^41^

Nevertheless, most existing workflows treat NMR, chromatography, and bioassay data separately or in limited pairwise combinations. Few protocols have been developed to systematically correlate these three orthogonal data types within a reproducible and generalizable framework. Furthermore, existing strategies rarely bridge statistical correlation analysis with direct structure identification using NMR databases, primarily due to spectral complexity and the absence of clean, database-compatible spectra for direct comparison.

To address these gaps, we introduce the “PLANTA” protocol (**P**hytochemica**L A**nalysis for **N**a**T**ural bio**A**ctives), a unified analytical workflow for the detection and identification of bioactive compounds in complex mixtures prior to isolation. PLANTA protocol integrates ^1^H NMR profiling, HPTLC separation, and bioassays with statistical correlation strategies to generate high-confidence predictions of active constituents. This enables the rapid multiparametric fingerprint of the sample, leading to the efficient detection of bioactive compounds within crude herbal extracts prior to isolation (Figure 1). Two novel components distinguish this approach:

- STOCSY-guided targeted spectral depletion. This method enhances dereplication by isolating statistically covarying NMR peaks (via STOCSY), while selectively removing nonmatching peaks from the full spectrum to reveal overlapping or hidden signals. The resulting “depleted” spectrum represents a quasi-pure fingerprint that can be directly compared with known entries in NMR databases. To our knowledge, this is the first method to bridge STOCSY-based correlation with practical database-compatible dereplication.
- SH-SCY (**S**tatistical **H**eterocovariance–**S**pectro**C**hromatograph**Y**). This newly developed technique enables bidirectional correlation between NMR and HPTLC datasets. It allows for the assignment of HPTLC bands to individual NMR peaks and vice versa, facilitating compound tracking across platforms and improving dereplication confidence. SH-SCY thus provides a crucial layer of orthogonal validation that strengthens compound assignment beyond what spectral or chromatographic data can provide independently.

The PLANTA protocol is designed with two implementation paths, depending on how biological activity is evaluated (Figure 1). Option 1, presented in this study, employs in vitro bioassays of chromatographic fractions and integrates NMR-HetCA and HPTLC-sHetCA analyses, followed by SH-SCY-based NMR–HPTLC cross-correlation and dereplication using STOCSY-guided spectral depletion in combination with NMR databases. Option 2, not showcased here, uses HPTLC-bioautography to directly localize bioactive zones on the plate, bypassing the HetCA analyses, and performs identification through cross-correlation with ^1^H NMR spectra and comparison with reference libraries. Both options are structured to enable early stage detection and dereplication of bioactive compounds, but differ in their reliance on in vitro assays versus effect-directed detection.

**Figure 1:**
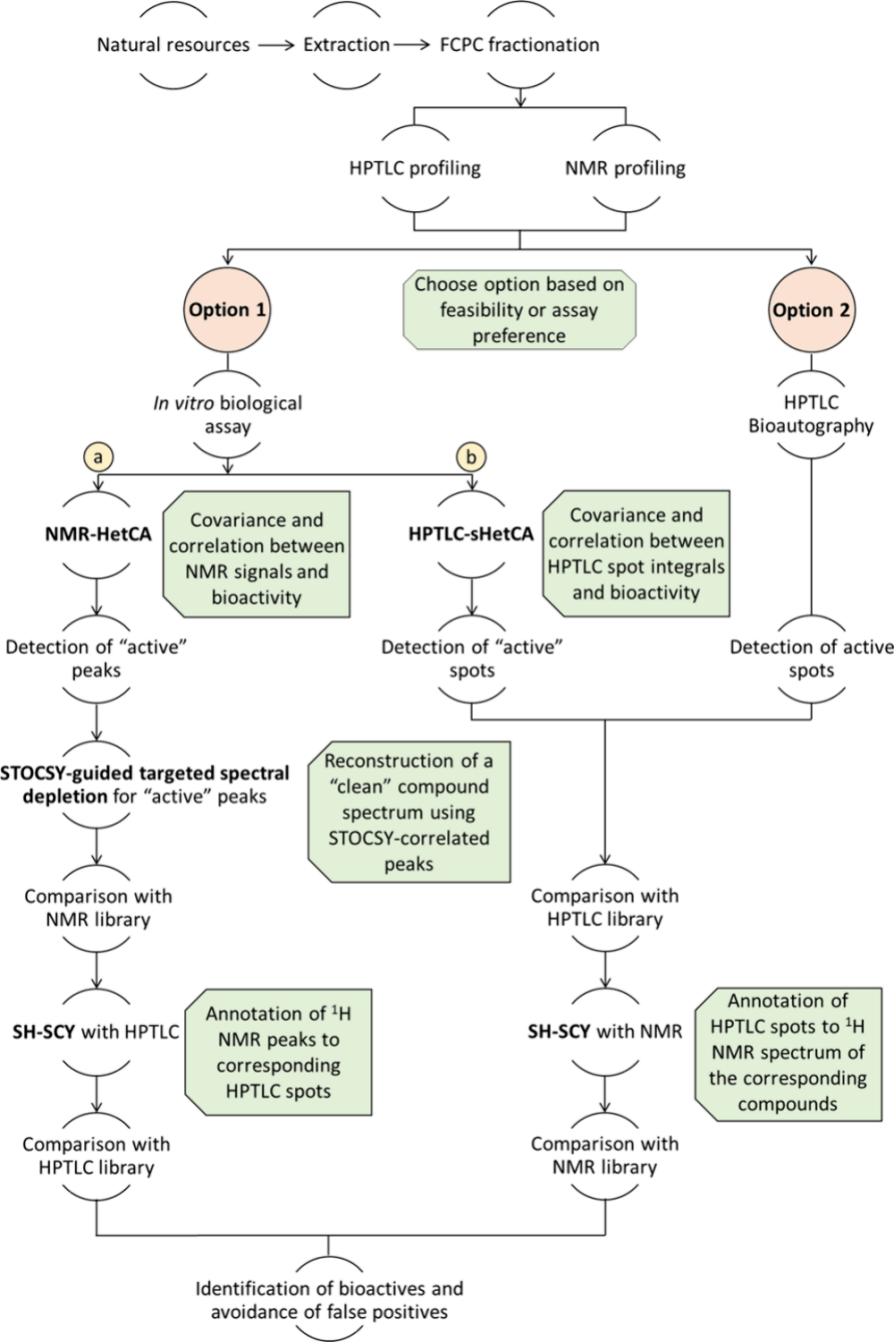
Workflow of the PLANTA protocol.

This study applies the PLANTA protocol to a complex artificial extract (ArtExtr) composed of 59 standard compounds chosen to reflect the chemical diversity and spectral overlap, typical of natural plant extracts, as a case study. The ArtExtr was treated as an unknown sample. By integrating multivariate statistical correlation with visual chromatographic mapping and bioactivity profiling, we demonstrate that the PLANTA protocol achieves high sensitivity and specificity in identifying active constituents. Quantitative performance metrics, including detection and correct identification rates, are reported as a benchmark for untargeted dereplication workflows.

## EXPERIMENTAL SECTION

### ArtExtr Composition and Prior Analyses

The ArtExtr preparation, FCPC fractionation, and evaluation of free radical scavenging activity by the DPPH assay, as well as the procedures related to the software-based pre- and post-processing of HPTLC chromatograms and densitograms were reported previously^21,41^ and are described in detail in the Supporting Information. In contrast, the experimental procedures for NMR spectroscopy and data pretreatment, NMR-HetCA, HPTLC, and HPTLC-sHetCA, also originally described in these works, are reproduced below to provide a complete and self-contained description of the methods relevant to the present study.

### NMR Spectroscopy and Data Pretreatment

The ^1^H NMR data used in this study were generated and reported previously.^21^ The acquisition and processing parameters are reproduced here to provide a complete description of the workflow. The samples were dissolved in methanol-*d*_4_ containing tetramethylsilane (TMS) as a reference (Euriso-Top, Saint-Aubin, France) at a concentration level of 10 mg/mL for the unfiltered ArtFrct samples and 3 mg/mL for the filtered ArtExtr FCPC samples. After sonication (5 min) in an Ultra Sonic bath (Elma Schmidbauer GmbH, Singen, Germany), 650 μL was transferred to 5 mm NMR tubes (LabScape, Bruker, Germany). The ^1^H NMR spectra were acquired at 298 K ± 0.1, after a 5 min resting period for temperature stabilization, on a Bruker Avance III 600 MHz NMR spectrometer equipped with a 5 mm PABBI 1H/D-BB inverse detection probe. Experiments were performed in automation mode using a BACS-60 sample changer operated by IconNMR. Data acquisition and processing were done with Bruker TopSpin 3.6. Profiling ^1^H NMR spectra were acquired using the water suppression 1D NOESY pulse program with the following settings: relaxation delay (d1) = 6 s, acquisition time = 2.73 s, FID (free induction decay) data points = 64 k, spectral width = 20 ppm, and number of scans = 128. The transmitter offset was set manually in order to achieve the optimal suppression of the residual water signal. FIDs were multiplied by an exponential weighting function corresponding to a line broadening of 0.3 Hz prior to Fourier transformation. Automated processing was carried out for phase correction and baseline correction. Chemical shift values were referenced to the residual methanol signal (3.31 ppm). ^1^H NMR spectral alignment was based on a segment-wise peak alignment and was performed in pairs, with the last one set as the “active” spectrum for the alignment of the next spectrum using MestRe Nova 14.2.1 (Mestrelab Research, Santiago de Compostela, Spain) using the implemented cross-correlation algorithm. The first derivative was used, while the missing values filling method was linear.

### NMR-HetCA

The NMR-HetCA analysis was originally performed previously.^21^ The methodology is briefly summarized here for completeness. Regions excluded from the integration of the ^1^H NMR spectra were the residual methanol-*d*_4_ signal (3.29–3.36 ppm) and the water peak (4.76–4.82 ppm). NMR spectra were processed in the MATLAB environment (bucketing of spectra and correlation with DPPH radical scavenging activity) through HetCA as previously described.^20^ The covariance and correlation between NMR resonances and activity values were visualized through the generated NMR pseudospectra, i.e., the HetCA plots. Each point of the HetCA plots depicted the covariance, where positive or negative peaks indicated positive or negative covariance values, respectively. They were additionally color-coded according to the respective correlation coefficients, ranging from blue for those that show low correlation to deep red for those that show high correlation.

### High-Performance Thin Layer Chromatography (HPTLC)

The HPTLC chromatographic data were obtained in our earlier work.^41^ The main experimental steps are outlined here to ensure the protocol remains self-contained. For the chemical profiling of the FCPC fractions of the artificial extract, the filtered samples were rediluted in methanol at a concentration level of 3 mg/mL. Subsequently, 7 μL of each sample was applied on HPTLC normal phase aluminum plates (20 cm × 10 cm) precoated with silica gel 60 F_254_ (150–200 mm), and HPTLC reversed aluminum plates (20 cm × 10 cm) precoated with silica gel 60 RP-18 F_254S_ (150–200 mm) (Merck, Darmstadt, Germany) as 7 mm bands, using an automatic TLC Sampler 4 (ATS-4, CAMAG, Muttenz, Switzerland). The chromatographic separation was performed in the Automatic Developing Chamber 2 (ADC 2) with a solvent system consisting of Tol, EtOAc, and Fa (60/40/1 v/v/v) for the normal phase, and H_2_O, MeCN, and Fa (70/30/1 v/v/v) for the reversed phase, up to a migration distance of 75 mm (from the lower plate edge). The same conditions were used for the development of all the plates. The plates were then documented under 254 nm, 366 nm, and under white light after derivatization with the sulfuric vanillin reagent (SVR) with CAMAG Visualizer 2. The system was operating under the VisionCats 3.0 software (CAMAG, Muttenz, Switzerland).

### HPTLC-sHetCA

The HPTLC-sHetCA procedure was applied and reported previously.^41^ For clarity in the present manuscript, the essential aspects of the methodology are reproduced below. The sHetCA method was based on the correlation and covariance algorithms built into Excel (Microsoft Corporation, Redmond, WA, USA). An integration matrix, comprising the HPTLC densitogram peak integration values (columns) across all fractions (rows, Fr1–Fr70), together with the corresponding bioactivity values (% DPPH inhibition, final column), was used to generate a correlation matrix. Blank cells in the integration matrix indicate the absence of a spot in a given fraction. Covariance was calculated only for those spots that exhibited a positive correlation coefficient with the activity.

### STOCSY-Guided Targeted Spectral Depletion

STOCSY analyses were performed using option 5 of the free-to-use DAFdiscovery platform within the Python environment.^25^ on ArtExtr fractions Fr20–70. Driver peaks were selected at δ values with minimal signal overlap in the ^1^H NMR spectra (Table S6). STOCSY correlation plots were used to identify covarying signals statistically associated with each driver peak. Peak picking was performed in MestReNova (v14.2.1; Mestrelab Research, Santiago de Compostela, Spain) on the NMR spectrum of the fraction where the driver peak appeared with the highest intensity. The “compound view” feature was then used to visually isolate only the selected peaks. Peak integrals were calculated relative to the driver peak across adjacent fractions. Peaks exhibiting inconsistent integration behavior, indicating that they likely originated from different compounds, were excluded. The resulting reduced peak set (“depleted” spectrum) was treated as a quasi-pure fingerprint suitable for dereplication.

### SH-SCY Analysis (Statistical Heterocovariance–SpectroChromatographY)

SH-SCY analysis was performed using Option 3 of the DAFdiscovery platform,^25^ which is originally designed to correlate NMR data with bioactivity profiles. In this study, we adapted the input structure to enable cross-platform correlation between ^1^H NMR and HPTLC datasets. Analyses were conducted in both forward and reverse directions, as detailed below.

### Forward-Mode Analysis (NMR to HPTLC)

In this mode, absolute integration values of ^1^H NMR peaks^21^ were treated as the “Bioactivity” input and binned HPTLC densitograms (687 Rf points per sample),^41^ generated using the rTLC v1.0 software,^36^ were treated as the “NMR” input. Each compound was analyzed individually, using only the fractions in which it was detected by NMR. Both datasets were normalized prior to correlation to generate pseudospectra with interpretable covariance.

### Reverse-Mode Analysis (HPTLC to NMR)

Here, the peak integration values from the HPTLC densitograms^41^ were used as the “Bioactivity” input, and the binned ^1^H NMR spectra^21^ of the corresponding fractions served as the “NMR” input. The integration values for each peak were normalized and expressed as a percentage of the maximum integration for comparative scaling.

In both correlation directions, SH-SCY plots were generated, yielding either pseudospectra (forward mode) or pseudochromatograms (reverse mode). The plots depict covariance magnitude on the vertical axis, with data points color-coded according to the Pearson correlation coefficient: blue indicates low correlation, and deep red indicates strong positive correlation.

### NMR Database Matching

Following STOCSY-guided spectral depletion and SH-SCY analysis, the resulting reduced ^1^H NMR peak sets were matched against an in-house NMR spectral database using the Mnova DB module in MestReNova (v14.2.1; Mestrelab Research, Santiago de Compostela, Spain). Matches with scores ≥ 900/1000 were considered reliable. In cases of peak misalignment or minor spectral overlap, lower scores were accepted based on manual inspection and consistency with chemical shifts and multiplicities.

## RESULTS AND DISCUSSION

The ArtExtr was prepared to simulate a crude plant extract concerning the structural diversity (Table S2, Figure S1). The mixture was fractionated by FCPC and the resulting fractions were evaluated for their DPPH radical scavenging activity in vitro. The fractions’ chemical profiles were obtained using NMR spectroscopy and HPTLC. The application of NMR-HetCA and HPTLC-sHetCA highlighted the highly correlated signals and HPTLC spots, respectively, with the observed bioactivity for each fraction. These aspects of the study, including the ArtExtr preparation, the FCPC fractionation, the evaluation of free radical scavenging activity using the DPPH assay, the NMR spectroscopic analysis, data pretreatment, the application of the NMR-HetCA methodology, the HPTLC and HPTLC-sHetCA methodology were previously detailed in Amountzias et al.^21,41^

The NMR-HetCA^21^ and HPTLC-sHetCA^41^ studies reported that a total of 30 compounds of the ArtExtr were highlighted as bioactive. Of these, 17 were correctly identified as active, while 13 were false positive results (Tables S3 and S4).

The aforementioned tables establish the possibility of detecting different substances through each methodology. For instance, the active substance caffeic acid was detected by NMR but not annotated in HPTLC. Conversely, the active substance curcumin was correctly predicted as active by HPTLC-sHetCA, in contrast to NMR-HetCA. Given these results, it was deemed appropriate to study the ArtExtr as if its components were unknown. Thus, HPTLC-detected substances were assigned new code names, according to their elution order (Table S5). Spot 23 (curcumin, Rf_NP_ = 0.52) in the HPTLC-sHetCA study was observed in fractions Fr20–24 and Fr32–34 (Figure S2). This was attributed to curcumin’s existence in two tautomeric forms (diketo- and enolic keto-form).^42^ Identification was confirmed by comparison of the chromatograms and the ^1^H NMR spectrum of the standard compound. Since the ArtExtr was treated as an unknown extract, the spots from fractions Fr20–24 and Fr32–34 were considered distinct and designated with different codes (HPTLC-I and HPTLC-R, respectively). According to the HPTLC-sHetCA analysis, HPTLC-I was classified as active (Cor = 0.38, Cov = 1.05), while HPTLC-R was classified as inactive (Cor = −0.94, Cov = −4.11). The reason for this false negative result was the coelution of more active compounds (e.g., baicalein) at higher concentrations that overshadowed HPTLC-R’s activity.

### Investigating the ArtExtr Fractions Chemical Composition

The study of the chemical profile is an essential step to obtain information about the contained metabolites. To fully characterize the chemical composition, it is essential to identify characteristic features in both HPTLC and NMR data. In HPTLC, the detection of UV-active compounds and the observation of their colors, either under UV light or following derivatization, can provide valuable insights into the metabolites’ chemical categories.^43^ Likewise, ^1^H NMR spectra often display diagnostic peaks that reveal the presence of specific compound classes. Consulting the literature and relevant databases (e.g., CAS, Reaxys, PubChem, PubMed, lotus.naturalproducts.net, Scopus, ScienceDirect, and Wikidata) can further aid in interpreting these features and gain information about the reported and/or isolated secondary metabolites from the organism of interest and highlights the potential biological activities of the target species or related species within the same genus or family.

Following the ArtExtr FCPC fractionation,^21^ the chemical profiles of the fractions were thoroughly investigated using HPTLC (Figure S3) and ^1^H NMR (Figure S4).

The HPTLC chromatograms revealed the presence of various classes of compounds, including:

- Flavonoids (yellow spots prior to and after derivatization with SVR, absorb at 254 nm, do not fluoresce at 366 nm, and usually fluoresce with a light blue color at 366 nm after derivatization with SVR^44^)
- Terpenoids (blue and purple spots after derivatization with SVR^43^)
- Compounds with extensive π-conjugated systems (intense blue spots at 254 and 366 nm^45^)
- Examination of the ^1^H NMR spectra revealed the presence of characteristic peaks indicative of substances belonging to the following categories:
- Flavonoids and glycosides of flavonoids, mainly kaempferol and quercetin derivatives. Characteristic peaks of H6 and H8 (d, 6.2 and 6.4 ppm, respectively), as well as the B-ring with H2′/H6′ being equivalent (8.1 ppm) for the disubstituted ring kaempferol derivatives and an ABX coupling system in three substituted ring for H6′ (7.8 ppm, d, *J* = 2.0 Hz), H2′ (7.6 ppm, dd, *J* = 8.0/2.0 Hz) and H3′ (6.9 ppm, d, *J* = 8.0 Hz) for the quercetin derivatives
- Simple phenolics (MeO-Phe groups in the range 4.0–3.6 ppm) and cinnamic acid derivatives (characteristic peaks of *trans* double bonds, attached to an aromatic ring 7.4/6.2 ppm)
- Gallic acid derivatives (single peak at 7.06 ppm)
- Aldehydes (peaks in the range 11.0–9.5 ppm)
- Terpenoids (methyl peaks in the range 1.6–0.6 ppm)
- Fatty acids (peak at 1.2 ppm)

Based on the above, the statistical analysis was expected to identify substances such as gallic acid derivatives and flavonoids, as these are known from the literature to be active against DPPH.^46,47^

### Utilization of HetCA Studies for the Identification of Bioactive Metabolites

Following the investigation of the ArtExtr fractions’ chemical profiles, guided by literature review, as well as the in vitro evaluation of their DPPH activity (Figure S5), HetCA analyses were conducted using both NMR and HPTLC data, according to PLANTA protocol. Specifically, the previously conducted NMR-HetCA^21^ and HPTLC-sHetCA^41^ methods were used as the basis for identifying bioactive signals (Figures S6 and S7, respectively). The NMR-Total-HetCA pseudospectrum and the covariance/correlation output (HPTLC) were retrieved from these studies and reanalyzed for dereplication purposes. The identification of the bioactive compounds was achieved through database matching, as well as the comparison with standard compounds and supporting literature data.

### Application of STOCSY-Guided Targeted Spectral Depletion and Direct Comparison with an In-House ^1^H NMR Spectral Database for Compound Identification

In accordance with the PLANTA protocol, STOCSY-guided targeted spectral depletion was applied following the analysis of the NMR-Total HetCA plot,^21^ which was prioritized due to its lower rate of false positives compared to HPTLC-sHetCA (Table S4). STOCSY was conducted on fractions Fr20–70 using the DAFdiscovery platform.^25^ The initial 18 fractions (Fr02–19) were excluded from the analysis due to their negligible DPPH-scavenging activity (<5.0%).

Driver peaks were located and selected at δ values with minimal overlap from neighboring signals in the ArtExtr ^1^H NMR spectra (Table S6). In several cases, STOCSY plots revealed peaks that were statistically correlated but did not originate from the same compound, as determined by inconsistent integration ratios (compared to the driver peak) across adjacent fractions. To address this, correlated peaks from the STOCSY output were manually selected within the NMR spectra using MestReNova (v14.2.1; Mestrelab Research, Santiago de Compostela, Spain), and the “compound view” function was used to isolate only the relevant peaks. These selected peaks were then integrated, and their ratios to the driver peak were measured across adjacent fractions. Peaks that deviated significantly from the expected trend were excluded via deselection (spectral depletion), producing a simplified spectrum resembling that of a single compound. The remaining peak set was compared to the in-house ^1^H NMR database constructed in MestReNova v14.2.1 for dereplication. This approach allowed the tentative identification of chemical classes and, in several cases, exact structural matching with database compounds. Representative examples illustrating this workflow are described below.

#### Example of the STOCSY-Guided Targeted Spectral Depletion Workflow–Driver Peak at 9.09 ppm

An STOCSY application workflow example is as follows. A red peak was detected in the NMR Total-HetCA pseudospectrum at 9.09 ppm (d, *J* = 1.4 Hz). Following the application of STOCSY, two peaks with very high correlation were highlighted (Figure 2).

**Figure 2:**
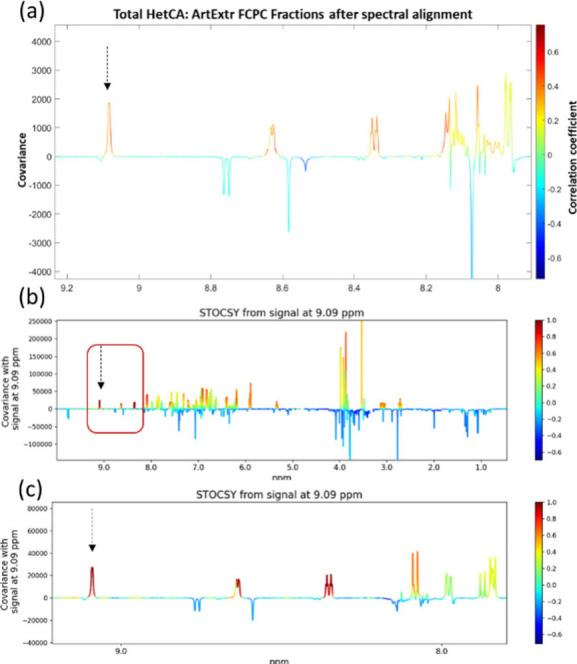
(a) NMR-Total HetCA plot region (9.20–7.80 ppm); (b) STOCSY pseudospectrum from the signal at 9.09 ppm (10.00–0.50 ppm) and (c) zoomed area (9.20–7.80 ppm).

This peak was detected in the fractions Fr30–60s ^1^H NMR spectra (Figure S8) and selected in the fraction where it exhibited the highest intensity (Fr38), along with the two highlighted peaks from the STOCSY pseudospectrum (Figure 3a). The remaining peaks were excluded using the compound view via the MestRe Nova 14.2.1 software (Figure 3b). After comparison with the spectral database, the result was nicotinic acid (RecordId 17, Table S2) with a score of 923/1000 (Figures 3c and 3d). The spectral comparison showed that these peaks correspond to either nicotinic acid or a derivative thereof with high confidence, despite the peak at 7.55 ppm not being visible in the ArtExtr fractions’ NMR spectra due to overlap with peaks of other substances present at higher concentrations.

**Figure 3:**
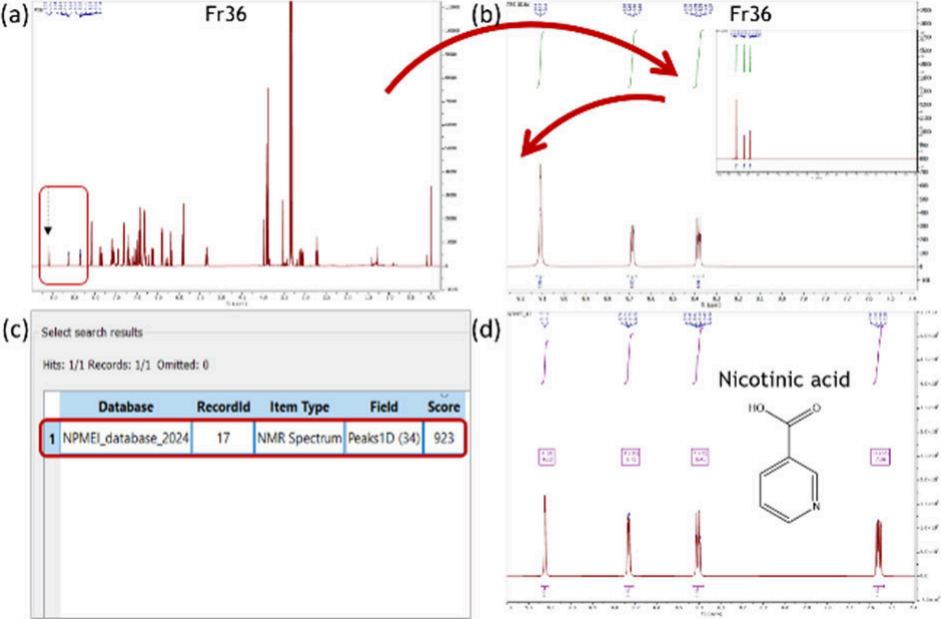
(a) ^1^H NMR spectrum of fraction Fr36 (9.15–0.00 ppm); (b) selection of the highlighted STOCSY peaks, application of spectral depletion and zoomed region (9.25–8.25 ppm); (c) results of the NMR spectral library; and (d) ^1^H NMR spectrum region of standard nicotinic acid (9.25–7.40 ppm). The driver peak that was integrated as 1.00 is indicated by a black arrow.

After this process, the corresponding peaks of the NMR-Total HetCA plot were assigned to the specific compound and excluded from the identification process (Figure S9).

#### Identification of Glycosides Based on Peaks of Aglycon–Driver Peak at 6.09 ppm

The peak at 6.09 ppm (m) (Figure S10) was detected in fractions Fr57–60. It was selected and integrated along with the rest of the STOCSY-derived peaks in fraction Fr59 (Figure S11­(a)–S11­(c)), where its intensity was the highest. In this case, all STOCSY highlighted peaks were selected, except those that correspond to the saccharide region (4.00–3.00 ppm), due to extensive overlapping that could lead to identification issues. After the spectral depletion and the comparison with the spectral library, the result was oleuropein (RecordId 24, Table S2) with a score of 942/1000 (Figures S11­(d) and S11­(e)). Due to the noninclusion of the glycoside ^1^H NMR peaks during the spectral depletion, the score was lower than 1000.

#### Identification Based on Partially Overlapped Driver-Peak–Driver Peak at 6.20 ppm

The peak at 6.20 ppm appears as a singlet in the NMR-Total HetCA plot (Figure S12­(a)), but it is, in fact, a doublet corresponding to a *trans* double bond (d, *J* = 15.9 Hz), as can be seen in the corresponding ^1^H NMR spectra (Fr41–50, Figure S12­(b)). Figure S12­(b) shows the two components of the double bond peak, with the selected component marked by a red dashed arrow (6.204 ppm) and the second component marked by a red arrow (6.231 ppm).

The reason for this phenomenon in the HetCA plot is the peak at 6.228 ppm (d, *J* = 2.2 Hz) which has a higher intensity, it is present in a similar number of fractions (Fr11–18 vs. Fr38–46) and has no correlation with the activity. The binning of the spectra in 0.005 ppm intervals resulted in the simultaneous presence of the two peaks in the same bin (6.225–6.230 ppm). This caused a decrease in the correlation coefficient with the activity for the component at 6.231 ppm. However, in the STOCSY plot, both components of the double peak at 6.20 ppm are clearly visible (Figure S12­(d)). This occurs because the fractions Fr02–19 spectra, where the peak at 6.228 ppm is located, were excluded during the calculation of the correlation coefficient and covariance, preventing the decrease in these values.

The driver peak at 6.20 ppm was located in fractions Fr41–50, as previously mentioned. Its highest intensity was located in the fraction Fr48, where it was selected and integrated along with the other peaks identified through STOCSY analysis (Figure S13). After spectral depletion based on integration values, the comparison with the spectral database revealed similarity to standard compounds caffeic acid, rosmarinic acid, ferulic acid and chlorogenic acid (RecordIds 10, 11, 41 and 50, respectively; see Table S2), all scoring a match of 1000/1000. Considering the STOCSY results, which showed no correlation with any peaks in the range of 5.00–0.50 ppm, as well as the NMR spectra where no such peaks are observed in this region, rosmarinic acid and chlorogenic acid were rejected (Figures S13­(c), S14­(c), and S14­(e)). The only difference between caffeic acid and ferulic acid is the presence of a methoxy- group (see Figures S14­(b) and S14­(d)), signals of which are absent in the fraction Fr48’s ^1^H NMR spectrum. Therefore, this substance has been annotated as caffeic acid.

Using the same methodology (Table S7), 14 compounds were reliably identified, while one was partially identified as a quercetin-type flavonol glycoside (see the Supporting Information and Figures S18–20), accounting for most of the highly correlated signals with the radical scavenging activity in the NMR-Total HetCA. To improve further the reliability of the identification results, an assignment to HPTLC spots was performed via SH-SCY, and the obtained results were compared with the chromatograms of standard compounds.

### Application of SH-SCY for the Assignment of NMR-HetCA “Active” Compounds to HPTLC Spots

To link NMR-HetCA-identified compounds with their corresponding HPTLC spots, SH-SCY analysis was applied in forward mode (NMR to HPTLC) using the DAFdiscovery platform^25^ (option 3). For each compound, the analysis was performed individually, correlating the integration values of the NMR driver peaks with the binned densitogram data from the same set of fractions. Only fractions where the compound was detectable by ^1^H NMR were included. Normalization of both datasets produced pseudospectra in which the covariance with specific HPTLC zones could be visualized. The most distinct and illustrative cases are presented below.

#### Use of the Chromatographic Data for the Correct Assignment to an HPTLC Spot Assignment of Rutin (Driver Peak at 7.67 ppm)

An example of ^1^H NMR peaks assignment to HPTLC spots is the driver peak at 7.67 ppm that corresponds to a compound that was identified with high confidence as a quercetin-type flavonoid with at least two sugar moieties, one of which is glucose, following the above-described methodology. The peak integration indicated that this specific compound is present in fractions Fr60–64. Comparison with the spectral database yielded a match with rutin (RecordId 24, Table S2) scoring 1000/1000 (Figures S15 and S16). The corresponding HPTLC spot is expected to absorb at 254 nm, appear yellow before and after derivatization with SVR, and fluoresce with a light blue color at 366 nm after derivatization.^44^ Figure 4 displays the cross-correlation between the ^1^H NMR driver peak at 7.67 ppm and the fractions Fr60–64 HPTLC densitograms, with the annotated HPTLC spots shown.

**Figure 4:**
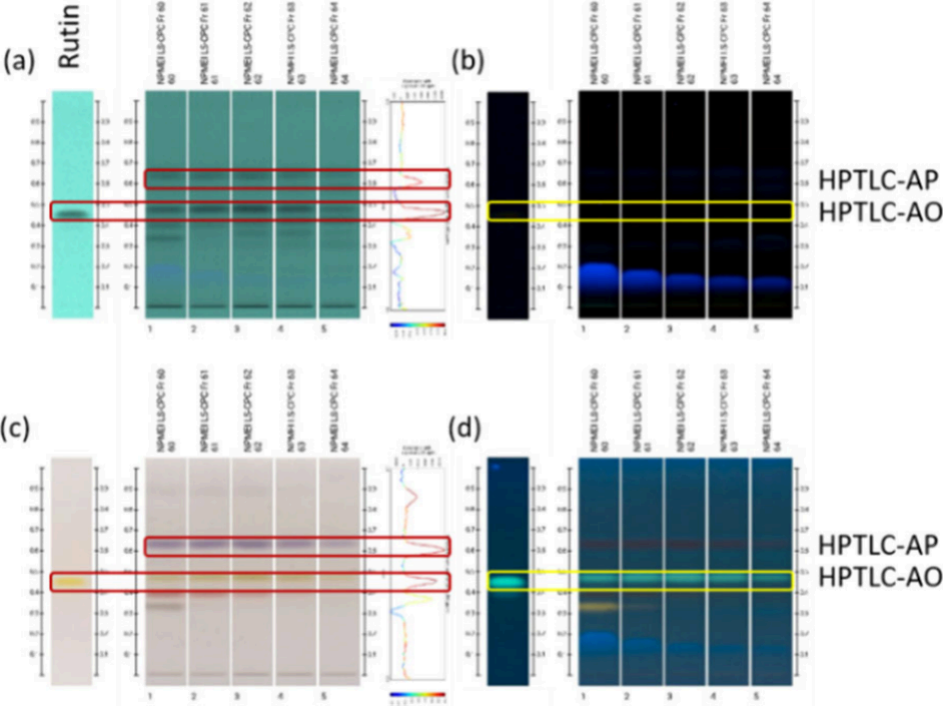
HPTLC comparison of standard rutin with the ArtExtr fractions Fr60–64 and SH-SCY between NMR and HPTLC for the driver peak at 7.67 ppm in RP (a) at 254 nm, (b) at 366 nm, (c) under visible light after derivatization with SVR, and (d) at 366 nm after derivatization.

Two correlations are observed at 254 nm in RP, with the spots HPTLC-AP (Rf 0.62, Cor = 0.82) and HPTLC-AO (Rf 0.47, Cor = 0.99). Correlations with the same spots appear after derivatization with SVR (Cor = 0.90 and 0.96, respectively). Both spots absorb at 254 nm, while HPTLC-AP appears as purple and HPTLC-AO as yellow after derivatization with SVR.

Based on the above, the spot HPTLC-AP did not exhibit flavonol compatible staining, while a comparison was performed with the standard rutin chromatogram for the verification of the result (Figure 4), and the spot HPTLC-AO was found to match that of the standard compound.

#### Use of NMR-HPTLC Cross-Correlation for the Assignment of Previously Not Detected Compounds—Assignment of Caffeic Acid (Driver Peak at 6.20 ppm)

The peak at 6.20 ppm in the ^1^H NMR spectrum corresponds to caffeic acid, as previously mentioned. This suggests that the corresponding HPTLC spot will exhibit absorption at 254 nm. The overlapping by other compounds in the HPTLC had initially led to the oversight of caffeic acid. Nevertheless, the use of SH-SCY between NMR and HPTLC enabled the annotation of this compound. Integration of this ^1^H NMR peak indicates that the compound is present in fractions Fr41–50. Figure S17 shows the cross-correlation of the ^1^H NMR driver peak at 6.20 ppm with the HPTLC densitograms of the fractions Fr41–50. Correlations with the spots HPTLC-Y and HPTLC-AD are observed. The spot HPTLC-Y absorbs at 254 nm, exhibiting a blue color in the fractions Fr37–48. The spot HPTLC-AD appears in the fractions Fr44–52 and also absorbs at 254 nm. Additionally, a light gray/purple spot appears in the fractions Fr41–50 under visible light after derivatization with SVR. This light gray/purple spot does not seem to correspond to either HPTLC-Y or HPTLC-AD, despite having the same Rf index, as it appears in the intermediate fractions. Comparison with the standard caffeic acid chromatogram (Figure S17) revealed that the light gray/purple spot corresponds to caffeic acid, whose spot at 254 nm is overlapped by the HPTLC-Y and HPTLC-AD spots. Consequently, this specific spot was designated as HPTLC-AR.

Overall, eight substances were reliably assigned to HPTLC spots using this method (Table S8), while the combination of NMR and HPTLC helped the partial identification of one more compound (quercetin-type flavonol glycoside) and its assignment to an HPTLC spot. Of the remaining compounds, two (baicalein and 3,5-dihydroxybenzoic acid) could not be assigned to specific HPTLC spots due to overlap, one because the development system was suboptimal (ellagic acid (dihydrate) Rf = 0.0), while three exhibited high covariance in their concentration, either between each other or with other compounds and could not be distinguished (nicotinic acid, caffeine, and sinapic acid).

### Identification of HPTLC-sHetCA “Active” Compounds via SH-SCY with NMR

The HPTLC-sHetCA analysis identified 22 putative bioactive compounds. Nine of these were assigned to specific metabolites using STOCSY-guided targeted spectral depletion (resveratrol, caffeic acid, rosmarinic acid, oleuropein, rutin, gallic acid, protocatechic acid, 6,7-dihydroxycoumarin and a quercetin-type flavonol glycoside). To assist in the assignment of the remaining bioactive HPTLC spots, SH-SCY analysis was employed in reverse mode (HPTLC to NMR) using Option 3 of the DAFdiscovery platform.^25^ In this setup, integration values from the densitogram peaks were treated as the “Bioactivity” input, and the binned ^1^H NMR spectra of the corresponding fractions served as the “NMR” data. Integration values were normalized for each HPTLC peak to generate pseudospectra with visible covariance. The most representative and interpretable cases are presented below.

#### Direct Identification via NMR Library-Spot HPTLC-N

An example of HPTLC spots identification via SH-SCY with NMR is the spot HPTLC-N (fractions Fr24–37, Rf_RP_ 0.27, Figure S23). Analysis of the chromatograms revealed that HPTLC-N absorbs at 254 nm and appears blue in color. Additionally, it fluoresces with a blue hue at 366 nm both prior to and after derivatization, while it does not react with SVR. Based on these observations, the corresponding compound is likely to contain π-conjugated systems.^45^ Figure 5 shows the results of SH-SCY between HPTLC and NMR. The peaks that showed the highest correlation with the spot HPTLC-N were located in fraction Fr33, where the HPTLC densitogram peak had the highest integration value (Figure S24). After spectral depletion and comparison of the ^1^H NMR data with the spectral database, the result was a match with umbelliferone (RecordId 58, Table S2) with a score of 1000/1000. To further validate this identification, the chromatograms of the standard umbelliferone and the ArtExtr fractions Fr24–37 were compared, confirming that the Rf index, absorption, and fluorescence properties of the spot HPTLC-N were consistent with those of the standard compound (Figure S25).

**Figure 5:**
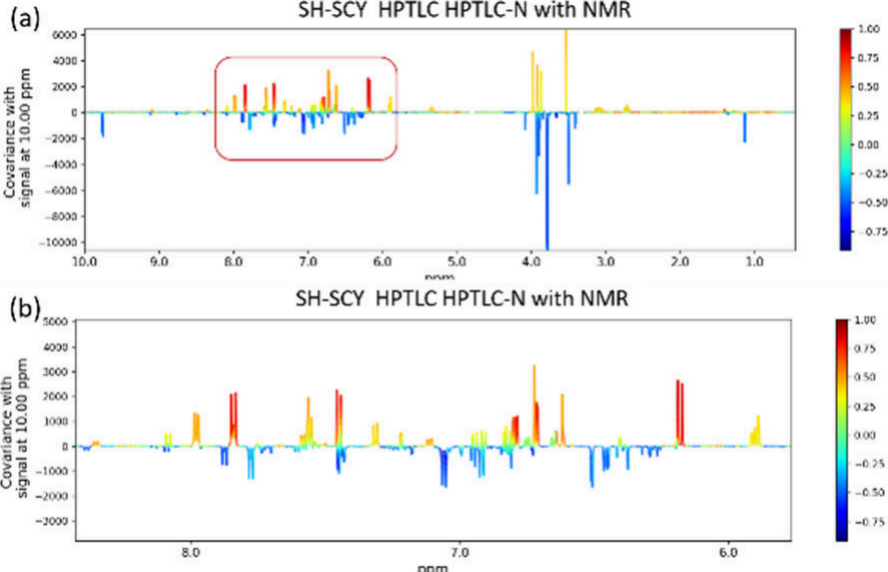
(a) SH-SCY pseudospectrum of HPTLC and NMR in the ArtExtr fractions Fr24–37 (10.00–0.80 ppm) for the spot HPTLC-N and (b) zoomed region (8.50–5.80 ppm).

#### Identification via NMR Library Based on the Aglycon-Spot HPTLC-Μ

Based on the chromatogram analysis, the spot HPTLC-Μ (fractions Fr24–29, Rf_NP_ 0.56, Rf_RP_ 0.09, Figure S31) absorbs at 254 nm, does not fluoresce at 366 nm either before or after derivatization, but instead appears pale orange after derivatization with SVR. Figure S32 depicts the results of SH-SCY between HPTLC and NMR. The peaks that showed the highest correlation with the spot HPTLC-Μ were located in the ^1^H NMR spectrum of the fraction Fr24, where the HPTLC peak showed the highest integration value (Figure S33­(a)). After spectral depletion of the peaks that did not correspond to the same compound according to the integration values (see Figures S33­(b) and S34), a comparison was performed with the spectral database. The results indicated naringin (narigenin glycoside) and narigenin, with scores of 875 and 687 out of 1000, respectively (see Figures S3­(c) and S3­(d)). Naringin can be easily rejected since there are no sugar signals in the ^1^H NMR specific fractions spectra. However, since the database results correspond to the same aglycon part of the compound, the chromatograms of standard narigin and the fractions Fr24–29 were compared (Figure S35). The results confirmed that the Rf index, absorption, and color after derivatization of the spot HPTLC-M were consistent with those of the standard compound. This result is in agreement with the report by Lawag et al.^44^ regarding the appearance of narigenin in HPTLC.

During this process, 10 out of the 15 spots that were not already assigned to a substance were reliably identified, while one more was partially identified as a caffeic acid derivative (see Figures S36–S38, and Table S9). It is worth noting that the spots HPTLC-Q and HPTLC-P were effectively assigned to caffeine and nicotinic acid, respectively, complementing the previous method where they were not assigned with high reliability to an HPTLC spot. Of the remaining spots, two exhibited the same covariance in their integration values (HPTLC-V and HPTLC-W), so the SH-SCY plot showed signals from both compounds. Furthermore, two spots had a very low concentration and were detected only in the HPTLC due to their high absorption index (ε) (HPTLC-AC and HPTLC-AE).

### Investigation of the Identified Compounds Activity via the Activity Database

The identified compounds activity was investigated using a biological activity database (Table S2), which led to the rejection of eight false positive results (Table S10).

The application of the “PLANTA” protocol led to the detection of 17 of the 19 (89.5%) active substances included in the study and the identification of 14 of them. Moreover, there were five false positive results due to inability for identification (Tables S10 and S11).

## CONCLUSIONS

This study presents Option 1 of the PLANTA protocol, a chemometric pipeline for the dereplication of bioactive compounds in complex mixtures, which integrates ^1^H NMR spectroscopy, HPTLC-UV/vis, and in vitro bioassays. While Option 2, based on HPTLC-bioautography, offers a more rapid route to identifying bioactive components, the more technically demanding Option 1 was chosen to demonstrate the protocol’s robustness in cases where bioautography is not feasible. Both paths ultimately converge in their ability to cross-correlate orthogonal analytical data and dereplicate compounds with high confidence.

The protocol showed strong identification performance in an artificial extract with considerable signal overlap. However, fractionation schemes and HPTLC development systems may require adjustment when applied to other extracts, depending on sample complexity. Despite this, PLANTA protocol is modular and adaptable, allowing the workflow to be tuned to the polarity, matrix type, and chromatographic behavior of the analytes under study. Further studies with diverse NMR solvents (e.g., D_2_O, DMSO-*d*_6_, CDCl_3_) could expand the protocol’s applicability across the full polarity range of NPs extracts.

STOCSY-guided targeted spectral depletion for compound identification performed well, but it is crucial to locate ^1^H NMR peaks that exhibit both high correlation and covariance with the driver peak in the respective fractions. Peaks within a similar spectral range that are not strongly correlated with the driver peak can affect coefficient calculations, potentially leading to the omission of target compound peaks. Examining the ^1^H NMR spectra of corresponding fractions helps address this issue, although unresolved or overlapping peaks may remain undetected. To enhance identification, peak selection and integration should be performed on the fraction where peaks have the highest intensity and minimal overlap. Targeted spectral depletion improves clarity, especially when using the compound view in the MestRe Nova 14.2.1 software, but must be applied carefully to avoid excluding peaks that are part of the target compound. While the STOCSY pseudospectrum offers valuable guidance, direct examination of ^1^H NMR spectra across adjacent fractions is essential to differentiate overlapping compounds. A spectral database aids classification but should be cross-checked against fraction spectra to ensure all relevant peaks are considered. To our knowledge, STOCSY-guided targeted spectral depletion represents the first approach that bridges statistical correlation analysis with practical compound identification via standard NMR databases. By converting correlated signal clusters into simplified, database-compatible spectra, this method enables direct dereplication.

For the cross-correlation of NMR and HPTLC, SH-SCY proved valuable for assigning compounds across orthogonal datasets. Successful application requires a good understanding of both the NMR spectral features and the chromatographic behavior of the metabolites involved. In cases where the cross-correlation cannot be performed reliably due to overlapping spots, performing SH-SCY on specific fractions is recommended. Moreover, in cases where the development systems used in HPTLC are suboptimal and result in very low or high Rf values, using alternative development systems or conducting two-dimensional HPTLC on targeted fractions is considered appropriate.

Looking ahead, the protocol could enable rapid isolation of identified target compounds through preparative HPTLC, while their Rf values could be translated to other techniques, such as HPLC. This is crucial for new NPs, since further investigation of their bioactivity, toxicity, and stereochemistry is required. Using various derivatization reagents in HPTLC could provide insights into the chemical classes present in the original mixture and produced fractions, improving the success rate of the protocol. This procedure could be further simplified by using the CAMAG TLC-MS Interface 2 system (CAMAG, Muttenz, Switzerland) or the Direct Analysis in Real Time mass spectrometry (DART-MS) system,^39,48,49^ which have the ability to extract mass data directly from HPTLC spots. Further research is needed for the proper integration of additional analytical techniques into the protocol, such as LC-MS and GC-MS, as well as to explore more complex bioactivity assays and to incorporate in silico docking models to evaluate binding affinities. This is necessary to account for the potential synergistic and antagonistic effects of the mixture components. Additionally, this protocol can be complementary to other existing methods for the detection of bioactive NPs.

Finally, although NMR-HetCA was originally implemented in MATLAB, the same analysis can also be performed using DAFdiscovery.^25^ In combination with ongoing developments in open-source chromatography software and instrumentation,^34,37,38^ this helps ensure that the PLANTA protocol remains largely accessible beyond proprietary environments.

## Supplementary Material



## Notes

Additional prior experimental procedures (ArtExtr preparation, FCPC, DPPH scavenging activity evaluation and software for pre- and post-processing of HPTLC chromatograms and densitograms), additional details for STOCSY and SH-SCY analyses, as well as HPTLC chromatograms, ^1^H NMR spectra, STOCSY, HetCA and SH-SCY pseudospectra, additional methodology examples and tables of results regarding the PLANTA protocol and additional bibliography (PDF)
