# Supplementary material for: PLANTA Protocol for the Direct Detection and Identification of Bioactive Compounds in Complex Mixtures via Combined NMR-HPTLC-Based Heterocovariance
Source: Anal Chem. 2025 Oct 3;97(40):21843–52. doi: 10.1021/acs.analchem.5c02192 (PMC12529470; doi:10.1021/acs.analchem.5c02192)
Supplement: Supplementary file 1 [file ac5c02192_si_001.pdf]

SUPPORTING INFORMATION

PLANTA Protocol for the Direct Detection and Identification of Bioactive Compounds in Complex Mixtures via Combined NMR-HPTLC-Based Heterocovariance

Vaios Amountzias[1], Evagelos Gikas[2], Nektarios Aligiannis[1]*

[1]Department of Pharmacognosy and Natural Products Chemistry, Faculty of Pharmacy, National and Kapodistrian University of Athens, Panepistimiopolis Zografou, 15771, Athens, Greece;

[2]Department of Analytical Chemistry, Faculty of Chemistry, National and Kapodistrian University of Athens, Panepistimiopolis Zografou, 15771, Athens, Greece

Supplementary Methods 7

Solvents and reagents 7

ArtExtr Preparation 7

Fast Centrifugal Partition Chromatography (FCPC) 7

Table S1. Selected biphasic solvent systems for the FCPC stepwise elution-extrusion fractionation of the artificial extract (ArtExtr). 8

Evaluation of free radical scavenging activity by DPPH assay 8

Software for Pre- and Post-Processing of HPTLC Chromatograms and Densitograms 9

Table S2. Standard substances of the ArtExtr (name, code, molecular weight) and their scavenging activity against DPPH free radicals. 10

Figure S1. Structures of ArtExtr ingredients. 14

Table S3. Compounds predicted as active against DPPH via NMR-HetCA and HPTLC-sHetCA. 15

Table S4. Comparison of results between NMR-HetCA and HPTLC-sHetCA methods for the detection of compounds with activity against DPPH. 16

Table S5. New codification of HPTLC spots. 17

Figure S2. Chromatograms of ArtExtr fractions Fr20-34, where spot 23 is highlighted in NP. The chromatogram order is 254 nm, 366 nm, visible light before derivatization, visible light after spraying with sulfuric vanillin reagent and 366 nm after derivatization. 19

Figure S3. HPTLC chromatograms of FCPC fractions of the ArtExtr. (a) NP at 254 nm; (b) NP at 366 nm; (c) NP in visible light after spraying with sulfuric vanillin reagent; (d) RP at 254 nm; (e) RP at 366 nm and (f) RP in visible light after derivatization with sulfuric vanillin reagent. Figure reproduced from Amountzias *et al.* (manuscript reference 41) with permission. 20

Figure S4. ^{1}H NMR spectra of the ArtExtr fractions. The region 3.38-3.31 ppm corresponding to the methanol-d_4 peak has been excluded from the spectra for better visualization. Figure reproduced from Amountzias *et al.* (manuscript reference 41) with permission. 21

Figure S5. % DPPH scavenging activity of all the fractions of the ArtExtr at 75 μg/mL. Inhibition is expressed as mean ± SD (N=3). Figure reproduced from Amountzias *et al.* (manuscript reference 41) with permission. 22

Figure S6. Total HetCA plot obtained from covariance of NMR data with the biological activity of ArtExtr fractions against DPPH, after spectral alignment. Figure reproduced with modifications from Amountzias *et al.* (manuscript reference 21) with permission. 22

Figure S7. Substances predicted as active from the HPTLC-sHetCA Fr20-70 study. 23

Table S6. Selected peaks that showed high correlation and covariance with the activity against DPPH in the NMR-Total HetCA plot and that were used as driver peaks for the application of STOCSY. 23

Figure S8. ^{1}H NMR spectra of the ArtExtr fractions Fr30-60 (9.15-8.30 ppm) where the highlighted peaks from the STOCSY pseudospectrum are presented. 24

Figure S9. Total HetCA plot where the two peaks related to the driver peak at 9.09 ppm are marked with red Xs and excluded from the identification process. 25

Figure S10. (a) Region of the Total HetCA plot (6.15-5.85 ppm); (b) STOCSY pseudospectrum from the signal at 6.09 ppm in the ArtExtr fractions Fr20-70 (10.00-0.80 ppm) and zoomed regions (c: 7.65-5.85 and d: 4.40-1.60 ppm). 25

Figure S11. (a) STOCSY pseudospectrum from the signal at 6.09 ppm in the ArtExtr fractions Fr20-70 (10.00-0.80 ppm); (b) ^{1}H NMR spectrum of the fraction Fr59 (9.50-0.00 ppm); (c) selection of STOCSY resulting peaks and spectral depletion (7.60-1.50 ppm); (d) NMR database results, and (e) ^{1}H NMR spectrum of standard oleuropein (7.60-1.50 ppm). 26

Figure S12. (a) NMR-Total HetCA plot region (6.55-6.25 ppm), where the black dashed arrow shows the selected component, while the black arrow shows the second component of the peak of the *trans* double bond; (ii) stack plot of the spectra ^{1}H NMR of the ArtExtr fractions (Fr02-70), where the red dashed arrow marks the selected component of the trans double bond peak, while the red arrow marks the second component of the trans double bond peak; (c) STOCSY pseudospectrum from the signal at 6.33 ppm in the Fr20-70 fractions of ArtExtr (10.00-0.80 ppm) and zoomed regions (d: 8.00-5.95 and e: 4.00-3.35 ppm). 27

Figure S13. (a) ^{1}H NMR spectrum of the fraction Fr48 (8.00-0.00 ppm); (b) selection of STOCSY resulting peaks and spectral depletion (7.60-6.15 ppm); (c) remaining peaks (7.60-6.15 ppm) and (d) NMR database results. 28

Figure S14. (a) Spectral region (7.60-6.15 ppm) of the fraction Fr48 where the selected peaks are shown; (b) spectral region of the standard caffeic acid (7.60-6.15 ppm); (c) spectral region of the standard rosmarinic acid (7.60-2.95 ppm); (d) spectral region of the standard ferulic acid (7.65-3.60 ppm) and (e) spectral region of the standard chlorogenic acid (7.65-2.00 ppm). 29

Table S7. Compounds identified via the STOCSY algorithm and the NMR database. 30

Figure S15. (a) Region of Total HetCA plot (7.90-7.29 ppm); (b) STOCSY pseudospectrum from the signal at 7.67 ppm in the fractions Fr20-70 of ArtExtr (10.00-0.80 ppm) and zoomed areas (c: 7.75-6.15; d: 5.80-3.00 and f: 2.40-0.80 ppm). 31

Figure S16. (a) ^{1}H NMR spectrum of the fraction Fr62 (8.00-0.00 ppm); (b) selection of STOCSY resulting peaks and spectral depletion (8.00-1.00 ppm); (c) remaining peaks (8.00-1.00 ppm); (d) NMR library results and (e) ^{1}H NMR spectrum region of standard rutin (8.00-1.00 ppm). 32

Figure S17. HPTLC comparison of standard caffeic acid with the ArtExtr fractions Fr41-50 and SH-SCY between NMR and HPTLC for the driver peak at 6.20 ppm in NP (a) at 254 nm and (b) in visible light after derivatization with sulfuric vanillin reagent. 33

Partial identification of compounds with high covariance in their concentrations via combination of NMR and HPTLC – Driver peak at 6.38 ppm 33

Figure S18. (a) Region of Total HetCA plot (6.55-6.25 ppm); (b) STOCSY pseudospectrum from the signal at 6.38 ppm in the fractions Fr20-70 of ArtExtr (10.00-0.80 ppm) and (c) zoomed area (7.50-3.80 ppm). 34

Figure S19. (a) ^{1}H NMR spectrum of the fraction Fr55 (8.50-0.50 ppm) and (b) selection of STOCSY resulting peaks and spectral depletion (8.50-0.50 ppm). 35

Figure S20. Selected STOCSY peaks from the signal at 6.38 ppm in the fraction Fr54 ^{1}H NMR spectrum (7.45-6.65 ppm). 35

Figure S21. SH-SCY between NMR and HPTLC in the fractions Fr51-58 for the driver peak at 6.38 ppm in RP (a) at 254 nm and (b) in visible light after derivatization with sulfuric vanillin reagent. 36

Figure S22. SH-SCY between NMR and HPTLC in the fractions Fr51-58 for the driver peak at 6.38 ppm in RP at 366 nm, after derivatization with sulfuric vanillin reagent. 36

Table S8. Assignment of NMR-HetCA resulting compounds to HPTLC spots. 36

Figure S23. Chromatograms of the ArtExtr fractions Fr24-37, where the spot HPTLC-N in RP is highlighted. The order of the chromatograms is 254 nm, 366 nm, visible light after derivatization with sulfuric vanillin reagent, and 366 nm after derivatization. 37

Figure S24. (a) ^{1}H NMR spectrum of the fraction Fr33 (10.00-0.00 ppm); (b) selection of STOCSY resulting peaks (7.90-6.10 ppm); (c) NMR library results and (d) ^{1}H NMR spectrum region of standard umbelliferone (7.90-6.10 ppm). 38

Figure S25. Comparison of the chromatograms of standard umbelliferone with the chromatograms of the fractions Fr24-37 of ArtExtr in RP. The order of the chromatograms is 254 nm, 366 nm, visible light after derivatization with sulfuric vanillin reagent, and 366 nm after derivatization. 39

Identification via NMR database despite peak misalignment - Spot HPTLC-AJ 39

Figure S26. Chromatograms of the ArtExtr fractions Fr49-61, where the spot HPTLC-AJ is highlighted in RP. The chromatograms order is 254 nm, 366 nm, visible light after derivatization with SVR, and 366 nm after derivatization. 40

Figure S27. (a) SH-SCY pseudospectrum between HPTLC and NMR in the ArtExtr fractions Fr49-61 (10.00-0.80 ppm) for the spot HPTLC-AJ and zoomed areas (b: 8.40-6.00 and c: 4.30 - 2.70 ppm). 41

Figure S28. Region of ^{1}H NMR spectra (8.25-7.90 ppm) of ArtExtr fractions (a) Fr54, (b) Fr56, (c) Fr58 and (d) Fr59 depicting the shift displacement of some of the peaks that exhibited a high correlation coefficient with the spot HPTLC-AJ. 42

Figure S29. (a) ^{1}H NMR spectrum of the fraction Fr54 (10.00-0.00 ppm); (b) selection of STOCSY resulting peaks and spectral depletion (8.20-2.50 ppm); (c) remaining peaks (8.20-2.80 ppm); (d)

NMR library results and (e) ^{1}H NMR spectrum region of the standard harmine (8.20-2.80 ppm). 43

Figure S30. Comparison of the chromatograms of standard harmine with the chromatograms of the ArtExtr fractions Fr49-61 in RP. The order of the chromatograms is 254 nm, 366 nm, visible light after derivatization with SVR and 366 nm after derivatization. 44

Figure S31. Chromatograms of ArtExtr fractions Fr24-29, where the HPTLC-M spot is highlighted (a) in NP and (b) in RP. The order of the chromatograms is 254 nm, 366 nm, visible after derivatization with SVR and 366 nm after derivatization. 45

Figure S32. (a) SH-SCY pseudospectrum between HPTLC and NMR in the ArtExtr fractions Fr24-29 (10.00-0.80 ppm) for the spot HPTLC-M and zoomed areas (b: 9.20-5.80 and c: 4.20-2.60 ppm). 46

Figure S33. (a) ^{1}H NMR spectrum of the fraction Fr24 (10.00-0.00 ppm); (b) selection of STOCSY resulting peaks and spectral depletion (8.20-2.50 ppm); (c) remaining peaks (7.40-2.60 ppm); (d) NMR library results and (e) ^{1}H NMR spectrum region of the standard naringenin (7.40-2.60 ppm). 47

Figure S34. ^{1}H NMR spectra of ArtExtr fractions Fr24-27, in which the peaks at 8.09 ppm (black frame, excluded) and 7.31 ppm (green frame, selected) are highlighted and the inconsistency in peak integration is depicted. 48

Figure S35. Comparison of the chromatograms of standard naringenin with the chromatograms of the ArtExtr fractions Fr24-29 in RP. The order of the chromatograms is 254 nm, 366 nm, visible light after derivatization with SVR and 366 nm after derivatization. 49

Figure S36. Chromatograms of ArtExtr fractions Fr60-67, where the HPTLC-AO spot is highlighted in RP. The order of the chromatograms is 254 nm, 366 nm, visible after derivatization with SVR and 366 nm after derivatization. 50

Figure S37. (a) SH-SCY pseudospectrum between HPTLC and NMR in the ArtExtr fractions Fr60-67 (10.00-0.80 ppm) for the spot HPTLC-AO and (b) zoomed area (7.80-6.00 ppm). 50

Figure S38. (a) ^{1}H NMR spectrum of the fraction Fr64 (10.00-0.00 ppm); (b) selection of STOCSY resulting peaks and spectral depletion (7.60-6.25 ppm); (c) remaining peaks (7.60-6.25 ppm) and (d) NMR library results. 51

Table S9. Identification of HPTLC-sHetCA resulting spots via SH-SCY with NMR. 52

Table S10. Summary table of results from the application of the "PLANTA" protocol for the detection of bioactive compounds against DPPH in the ArtExtr. 53

Table S11. Percentages of actives, false positive and false negative results predicted by the application of the PLANTA protocol for the identification of bioactive compounds against DPPH in the ArtExtr. 55

ADDITIONAL REFERENCES 56

Supplementary Methods

The detailed experimental procedures for the ArtExtr preparation, FCPC fractionation and evaluation of free radical scavenging activity by the DPPH assay, as well as the procedures related to the software-based pre- and post-processing of HPTLC chromatograms and densitograms are reproduced below from Amountzias *et al.*, 2024 (manuscript references 21 and 41).

Solvents and reagents

All solvents were of analytical grade and were purchased from Merck (Merck, Darmstadt, Germany), while 2,2-diphenyl-1-picrylhydrazyl (DPPH) was purchased from Sigma-Aldrich (Sigma-Aldrich, Steinheim, Germany). Water was produced by a LaboStar PRO TWF system (Evoqua Water Technologies, Pittsburgh, USA).

18 beta-glycyrrhetinic acid, 2,4-dimethoxyphenylacetic acid, 3,5-dihydroxybenzoic acid, 4-hydroxybenzaldehyde, 4-hydroxybenzoic acid, 6,7-dihydroxycoumarin (esculetin), arbutin, aristolochic acid, baicalein, biochanin A, caffeic acid, caffeine, catechol, chlorogenic acid, colchicine, *p*-coumaric acid, curcumin, daidzein, diosgenin, ellagic acid (dihydrate), ephedrine, ferulic acid, galantamine hydrobromide, gallic acid, harmine, hesperetin, homovanillic acid, *p*-hydroxyphenylacetic acid, kaempferol, naringenin, naringin, nicotinic acid, oleanolic acid, oleuropein, oxytetracycline hydrochloride, palmitic acid, protocatechic acid, quercetin, D-(−)-quinic acid, reserpine, resveratrol, rosmarinic acid, sclareol, shikonin, sinapic acid, sucrose, tannic acid, thymol, umbelliferone, vanillic acid and vanillin were purchased from Sigma-Aldrich (Sigma Aldrich, Steinheim, Germany). Aucubin, *m*-coumaric acid, isoferulic acid and loganin were purchased from Supelco (Supelco, Inc, Sigma Aldrich, Steinheim, Germany). (−)-Scopolamine methyl bromide, phlorizin quercitrin and rutin were purchased from PhytoLab (PhytoLab GmbH & Co. KG, Vestenbergsgreuth, Germany) and trolox from Acros Organics (Acros Organics B.V.B.A., Thermo Fisher Scientific, USA).

ArtExtr Preparation

A mixture (ArtExtr) composed of 59 standard compounds (Table S2 and Figure S1) was prepared with 50 mg of each compound diluted in 50 mL of MeOH. Mole fractions of all compounds were used in order to simplify the concentration and activity covariance study.

Fast Centrifugal Partition Chromatography (FCPC)

The fractionation of the ArtExtr was performed by FCPC (FCPC KROMATON, France) with a 1000 mL column and adjustable rotation of 650-1700 rpm, equipped with a Gilson PLC 2250 pump and fraction collector compact system (Gilson Incorporated, Middleton, USA). The ArtExtr was fractionated using a step-gradient elution-extrusion normal phase method consisting of n-Hept, EtOAc, n-BuOH, MeOH and H_2O in ascending mode, starting with the mobile phase of S1 and gradually increasing the polarity by consecutively applying the mobile phases of S2-S8 (Table S1). More specifically, the 1000 mL column was initially filled with the stationary phase (i.e. the lower phase) of S1 (Table S1) at 650 rpm, with a flow rate of 50 mL/min. After increasing the

rotation to 1200 rpm, the mobile phase of S1 (i.e. the upper phase) was pumped through the column with a flow rate of 10 mL/min. The equilibration was reached after 65 mL and the sample (1.2 g dissolved in 50 mL MeOH) was injected, while the fraction collector was set to 20 mL/fraction. The volume of each of the mobile phases used was 800 mL, so 40 fractions/mobile phase were collected. Elution extrusion was performed with the lower phase of S8 with a flow rate of 30 mL/min at 1700 rpm and 36 fractions were collected. The extrusion was completed by applying 2 L of the lower phase of S8 that was collected in a flask and not included in the results. The total number of fractions collected was 367, which were then pooled to 69 fractions based on their TLC profiles, aiming to the optimal variance of the included compounds. The resulting 69 pooled fractions were diluted in methanol at a concentration level of 4 mg/mL, filtered with polytetrafluoroethylene (PTFE) filters (13 mm × 22 μm, RephiLe Bioscience Ltd, USA) and solvent evaporation was achieved under reduced pressure using an RVC 2-33 CDplus evaporator system equipped with an Alpha 2-4 LSCbasic freeze dryer (Martin Christ, Germany).

Table S1. Selected biphasic solvent systems for the FCPC stepwise elution-extrusion fractionation of the artificial extract (ArtExtr).

	n-Hept	EtOAc	n-BuOH	MeOH	H_2O
S1	9	1	0	5	5
S2	8	2	0	5	5
S3	7	3	0	5	5
S4	6	4	0	5	5
S5	5	5	0	5	5
S6	2	8	0	5	5
S7	2	8	1	4	5
S8	2	8	2	3	5

Evaluation of free radical scavenging activity by DPPH assay

The screening of the antioxidant activity of the standard substances and, subsequently, the FCPC fractions was estimated by the DPPH assay as previously described by Lee *et al.*.[1] Samples were diluted at a stock concentration level of 10 mg/mL in DMSO and 10 μL of each sample were mixed

with 190 μL of DPPH solution (≈ 0.317 mM, 12.5 mg DPPH/100 mL EtOH) in a 96-well plate and then subsequently incubated, at room temperature, for 30 min in darkness. Finally, the absorbance was measured at 517 nm, using the Tecan Infinite M1000 PRO reader (Tecan Austria GmbH), while the system was operating under the Tecan i-control v.1.11. All evaluations were performed in triplicates, while gallic acid was used as positive control (IC_{50} = 30.2 μM). The % inhibition of the DPPH radical for each dilution was calculated using the following formula:

$$\%\text{Inhibition} = \{[1-(A_S-A_B)]/A_C\}\times 100$$

where A_S is the absorbance of the sample, A_C the absorbance of the control and A_B the absorbance of the sample without the DPPH radical. Inhibition was expressed as mean ± SD (N = 3). The *in vitro* DPPH inhibition assay for the ArtFrcts, as well as for the standards comprising them, was performed at 50 μg/mL and 25 μg/mL. The standard substances comprising the ArtExtr were evaluated *in vitro* at 100 μg/mL and IC_{50} values were calculated for the most active ones. Regarding the ArtExtr FCPC fractions, the evaluation took place at a final concentration of 75 μg/mL in the well.

Software for Pre- and Post-Processing of HPTLC Chromatograms and Densitograms

The HPTLC chromatogram photos were processed using the free software rTLC v.1.0 (manuscript reference 36), which converted the photos into 1000 data points/track that were then exported as Excel CSV files. Of these, only 687 points/track constituted the corresponding densitogram, while the rest were the areas below the baseline and above the solvent front, so they were discarded. The experimental parameters were used to obtain the data (height of sample application, track application length, track distance, distance of the solvent front from the edge of the plate). Finally, all the zones were considered, excluding a total of 1 mm from the outer edges of the spots.

The densitograms were then processed using the free Fityk v. 1.3.1 software[2] to perform baseline correction, which was accomplished through the cubic spline method. In addition, the peaks at the respective wavelengths were integrated using the same software with the GaussianA function, and an integration table was created for statistical processing. Deconvolution of the peaks was applied when necessary.

Table S2. Standard substances of the ArtExtr (name, code, molecular weight) and their scavenging activity against DPPH free radicals.

Code	Name	Chemical category	DPPH activity (100 μg/mL)	IC_{50}* (μM)	Molecular Weight (g/mol)	mmoles	Molecular fraction (%) in ArtExtr
01	Galanthamine hydrobromide	Alkaloid	1.2 ± 0.4		368.30	0.14	1.13
02	Quercitrin	Flavonoid	91.4 ± 0.2	50.5	448.40	0.11	0.89
03	Quercetin	Flavonoid	96.6 ± 0.1	30.3	302.23	0.17	1.37
04	Kaempferol	Flavonoid	96.7 ± 0.0	68.1	286.24	0.17	1.37
05	Phlorizin	Chalcone	0.0 ± 0.6		436.40	0.11	0.89
06	Resveratrol	Stilbenoid	74.5 ± 0.7	225.4	228.24	0.22	1.78
07	Aristolochic acid	Monocarboxylic acid	0.0 ± 1.1		341.27	0.15	1.21
08	Palmitic acid	Fatty acid	0.0 ± 1.2		256.42	0.19	1.53
09	Reserpine	Alkaloid	0.0 ± 1.0		608.70	0.08	0.65
10	Caffeic acid	Phenolic acid	95.8 ± 0.1	44.7	180.16	0.28	2.26
11	Rosmarinic acid	Phenolic acid	95.8 ± 0.0	37.7	360.30	0.14	1.13
12	Ephedrine	Alkaloid	0.0 ± 0.4		165.23	0.30	2.42

13	Harmine	Alkaloid	0.0 ± 0.8		212.25	0.24	1.94
14	Oleanolic acid	Terpenoid	0.0 ± 0.4		456.70	0.11	0.89
15	Naringenin	Flavonoid	0.0 ± 0.4		272.25	0.18	1.45
16	Hesperetin	Flavonoid	40.2 ± 0.4		302.28	0.17	1.37
17	Nicotinic acid	Pyridinecarboxylic acid	0.4 ± 0.5		123.11	0.41	3.31
18	Shikonin	Naphthoquinone	41.0 ± 3.1		288.29	0.17	1.37
19	Thymol	Phenol	18.6 ± 0.6		150.22	0.33	2.66
20	Oxytetracycline hydrochloride	Tetracycline (Alkaloid)	8.0 ± 0.5		496.90	0.10	0.81
21	18-β-glycyrrhetinic acid	Terpenoid	0.0 ± 1.6		470.70	0.11	0.89
22	2,4-dimethoxyphenylacetic acid	Phenolic acid	0.0 ± 0.4		196.20	0.25	2.02
23	Curcumin	Polyphenol	95.6 ± 0.2	80.8	368.40	0.14	1.13
24	Oleuropein	Iridoid	96.2 ± 0.0	61.1	540.50	0.09	0.73
25	Sucrose	Sugar	0.0 ± 0.7		342.30	0.15	1.21
26	Rutin	Flavonoid	91.6 ± 0.0	46.8	610.50	0.08	0.65
27	Arbutin	Phenol	41.1 ± 0.5		272.25	0.18	1.45
28	*p*-Coumaric acid	Phenolic acid	1.4 ± 0.5		164.16	0.30	2.42

29	Homovanillic acid	Phenolic acid	66.5 ± 0.3	261.3	182.17	0.27	2.18
30	Ellagic acid (dihydrate)	Polyphenol	95.7 ± 0.0	20.8	338.22	0.15	1.21
31	Tannic acid	Polyphenol	96.4 ± 0.1	3.1	1701.20	0.03	0.24
32	Caffeine	Xanthine	0.0 ± 0.8		194.19	0.26	2.10
33	Vanillin	Benzaldehyde	2.4 ± 0.5		152.15	0.33	2.66
34	Gallic acid	Phenolic acid	95.7 ± 0.0	30.2	170.12	0.29	2.34
35	Biochanin A	Flavonoid	0.0 ± 0.4		284.26	0.18	1.45
36	Daidzein	Flavonoid	0.0 ± 0.3		254.24	0.20	1.61
37	Naringin	Flavonoid	0.0 ± 1.3		580.50	0.09	0.73
38	Catechol	Phenol	95.9 ± 0.1	48.2	110.11	0.45	3.63
39	3,5-dihydroxybenzoic acid	Phenolic acid	1.0 ± 0.3		154.12	0.32	2.58
40	D-(−)-quinic acid	Cyclitol	0.0 ± 0.9		192.17	0.26	2.10
41	Ferulic acid	Phenolic acid	90.2 ± 0.3	95.2	194.18	0.26	2.10
42	4-hydroxybenzoic acid	Phenolic acid	0.0 ± 0.6		138.12	0.36	2.91
43	*m*-Coumaric acid	Phenolic acid	2.2 ± 2.3		164.16	0.30	2.42
44	Isoferulic acid	Phenolic acid	22.4 ± 0.1		194.18	0.26	2.10
45	4-Hydroxybenzaldehyde	Benzaldehyde	0.0 ± 1.2		122.12	0.41	3.31
46	Sinapic acid	Phenolic acid	93.5 ± 0.1	82.2	224.21	0.22	1.78

47	Vanillic acid	Phenolic acid	6.1 ± 0.1		168.15	0.30	2.42
48	Diosgenin	Terpenoid	0.0 ± 1.0		414.60	0.12	0.97
49	*p*-Hydroxyphenylacetic acid	Phenolic acid	0.0 ± 0.7		152.15	0.33	2.66
50	Chlorogenic acid	Cyclitol	91.7 ± 0.5	87.9	354.31	0.14	1.13
51	Aucuboside	Iridoid	0.0 ± 0.9		346.33	0.14	1.13
52	Sclareol	Terpenoid	0.0 ± 0.0		308.50	0.16	1.29
53	Protocatechic acid	Phenolic acid	92.7 ± 0.3	110.6	154.12	0.32	2.58
54	(−)-Scopolamine methyl bromide	Alkaloid	0.0 ± 0.6		398.30	0.13	1.05
55	Loganin	Iridoid	0.0 ± 0.5		390.40	0.13	1.05
56	Baicalein	Flavonoid	95.4 ± 0.0	26.3	270.24	0.19	1.53
57	6,7-Dihydroxycoumarin (Esculetin)	Coumarin	95.2 ± 0.0	27.8	178.14	0.28	2.26
58	Umbelliferone	Coumarin	0.0 ± 0.4		162.14	0.31	2.50
59	Colchicine	Alkaloid	0.0 ± 0.3		399.40	0.13	1.05

*IC_{50}: half-maximal inhibitory concentration. Results are expressed as the mean ±SD of three independent experiments.

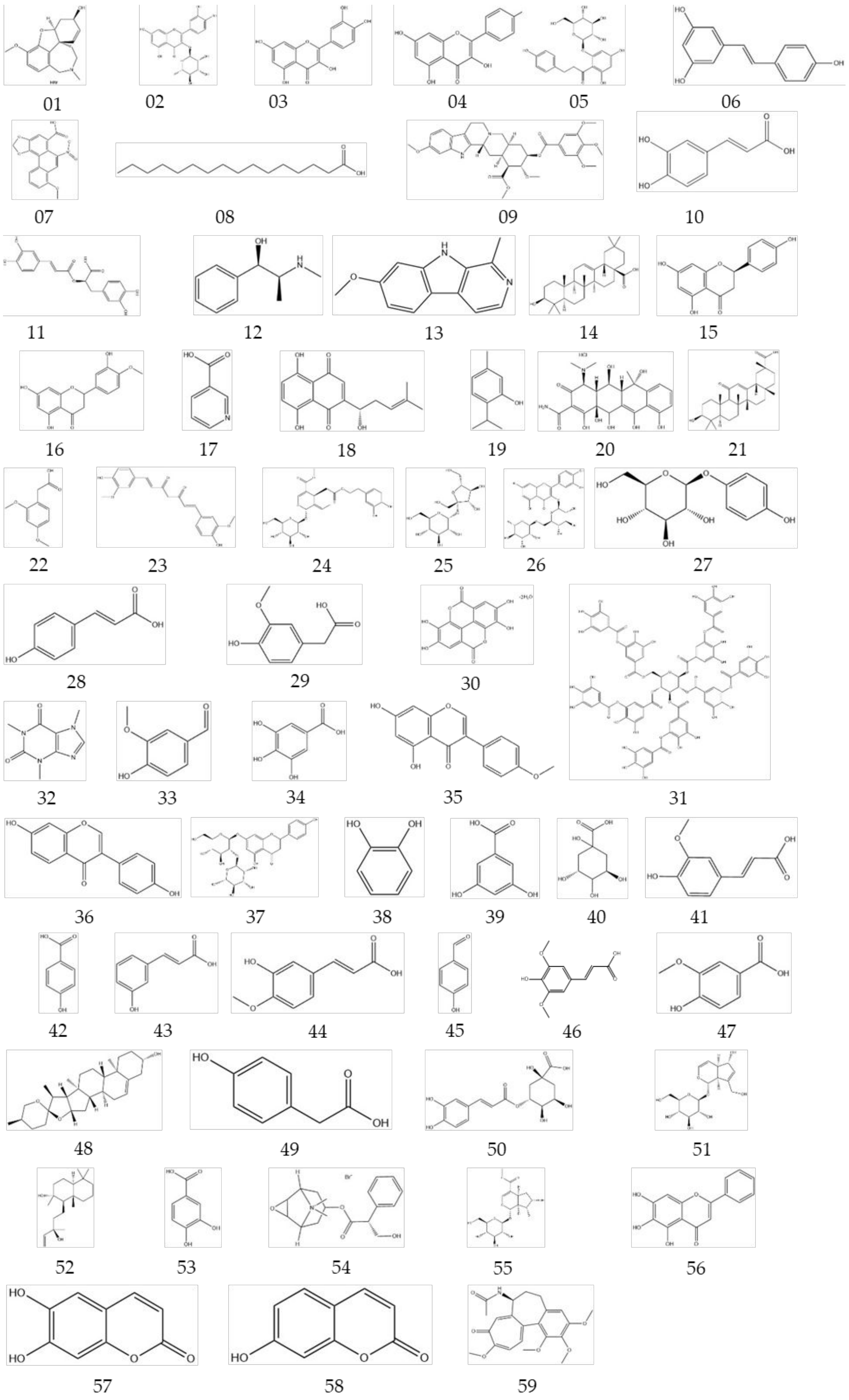

Figure S1. Structures of ArtExtr ingredients.

Table S3. Compounds predicted as active against DPPH via NMR-HetCA and HPTLC-sHetCA.

Code	Name	DPPH activity (100 μg/mL)	NMR-HetCA DPPH	HPTLC sHetCA DPPH Fr20-70
02	Quercitrin	91.4 ± 0.2	Correct	False negative
03	Quercetin	96.6 ± 0.1	N.D.	Correct
05	Phlorizin	0.0 ± 0.6	False positive	False positive
06	Resveratrol	74.5 ± 0.7	Correct	Correct
07	Aristolochic acid	0.0 ± 1.1	Correct	False positive
09	Reserpine	0.0 ± 1.0	n.i.	False positive
10	Caffeic acid	95.8 ± 0.1	Correct	N.D.
11	Rosmarinic acid	95.8 ± 0.0	Correct	Correct
12	Ephedrine	0.0 ± 0.4	n.i.	False positive
13	Harmine	0.0 ± 0.8	n.i.	False positive
15	Naringenin	0.0 ± 0.4	Correct	False positive
17	Nicotinic acid	0.4 ± 0.5	False positive	Correct
23	Curcumin	95.6 ± 0.2	N.D.	Correct
24	Oleuropein	96.2 ± 0.0	Correct	Correct
26	Rutin	91.6 ± 0.0	Correct	Correct
30	Ellagic acid (dihydrate)	95.7 ± 0.0	Correct	N.D.
32	Caffeine	0.0 ± 0.8	False positive	False positive
34	Gallic acid	95.7 ± 0.0	Correct	Correct
38	Catechol	95.9 ± 0.1	False negative	Correct

39	3,5-dihydroxybenzoic acid	1.0 ± 0.3	False positive	N.D.
41	Ferulic acid	90.2 ± 0.3	N.D.	Correct
46	Sinapic acid	93.5 ± 0.1	Correct	False negative
47	Vanillic acid	6.1 ± 0.1	N.D.	False positive
50	Chlorogenic acid	91.7 ± 0.5	N.D.	Correct
53	Protocatechic acid	92.7 ± 0.3	Correct	Correct
55	Loganin	0.0 ± 0.5	N.D.	False positive
56	Baicalein	95.4 ± 0.0	Correct	N.D.
57	6,7-Dihydroxycoumarin (Esculetin)	95.2 ± 0.0	Correct	Correct
58	Umbelliferone	0.0 ± 0.4	N.D.	False positive
59	Colchicine	0.0 ± 0.3	False positive	Correct

N.D.: Not detected, n.i.: Not included in the study.

Table S4. Comparison of results between NMR-HetCA and HPTLC-sHetCA methods for the detection of compounds with activity against DPPH.

**DPPH**	**NMR-HetCA**	**HPTLC-sHetCA**
Compounds included in the study	52	37
Identified compounds	33	37
Correct	27	25
Correct (%)	81.8	67.6
Active compounds included in the study	19	14

Correctly predicted active compounds	12	12
Correctly predicted active compounds (%)	63.2	85.7
Correctly predicted active compounds on the total active compounds of the mixture (%)	63.2	63.2
False positive results	5	10
False positive results (%)	25.0	43.5
False negative results	1	2
False negative results (%)	5.3	14.3

Table S5. New codification of HPTLC spots.

**New codification**	**Phase**	**λ (nm)**	**Fractions**	**Rf**	**Previous Code[a]**
HPTLC-A	NP	van	2-4	0.58	48
HPTLC-B	NP	van	2-8	0.65	14
HPTLC-C	NP	W	4-7	0.81	18
HPTLC-D	NP	van	4-9	0.32	52
HPTLC-E	NP	254	10-22	0.36	21
HPTLC-F	NP	254	11-18	0.7	35
HPTLC-G	NP	W	19-23	0.39	07
HPTLC-H	NP	254	19-23	0.55	36
HPTLC-I	NP	W	20-24	0.52	23a
HPTLC-J	RP	van	20-32	0.2	22
HPTLC-K	RP	254	21-31	0.29	33
HPTLC-L	RP	254	22-31	0.32	45
HPTLC-M	RP	van	24-29	0.09	15

HPTLC-N	RP	366	24-37	0.27	58
HPTLC-O	RP	van	25-32	0.4	38
HPTLC-P	NP	366	26-44	0.1	32
HPTLC-Q	RP	254	30-48	0.82	17
HPTLC-R	NP	W	32-34	0.52	23b
HPTLC-S	NP	van	32-37	0.57	16
HPTLC-T	RP	254	34-41	0.44	42
HPTLC-U	NP	254	35-40	0.37	43
HPTLC-V	NP	van	36-41	0.39	41
HPTLC-W	RP	254	36-41	0.45	47
HPTLC-X	RP	van	37-41	0.16	03
HPTLC-Y	RP	366	37-50	0.44	57
HPTLC-Z	RP	van	37-46	0.32	28
HPTLC-AA	RP	254	37-46	0.31	46
HPTLC-AB	NP	van	38-44	0.43	06
HPTLC-AC	RP	366	40-48	0.02	09
HPTLC-AD	RP	254	42-54	0.59	53
HPTLC-AE	RP	van	49-54	0.27	12
HPTLC-AF	RP	254	49-56	0.76	34
HPTLC-AG	RP	van	49-57	0.15-0.18	59
HPTLC-AH	RP	254	49-57	0.34-0.36	02
HPTLC-AI	RP	366	49-58	0.35	05
HPTLC-AJ	RP	254	49-61	0.1-0.3	13
HPTLC-AK	NP	van	54-59	0.05	11

HPTLC-AL	RP	van	58-60	0.32	24
HPTLC-AM	RP	van	60-63	0.89	27
HPTLC-AN	RP	van	60-63	0.39	37
HPTLC-AO	RP	254	60-65	0.48	26
HPTLC-AP	RP	van	60-66	0.61	55
HPTLC-AQ	RP	366	60-67	0.58	50
HPTLC-AR	RP	254	64-69	0.31	54

[a] The previous codification is according to Table S2.

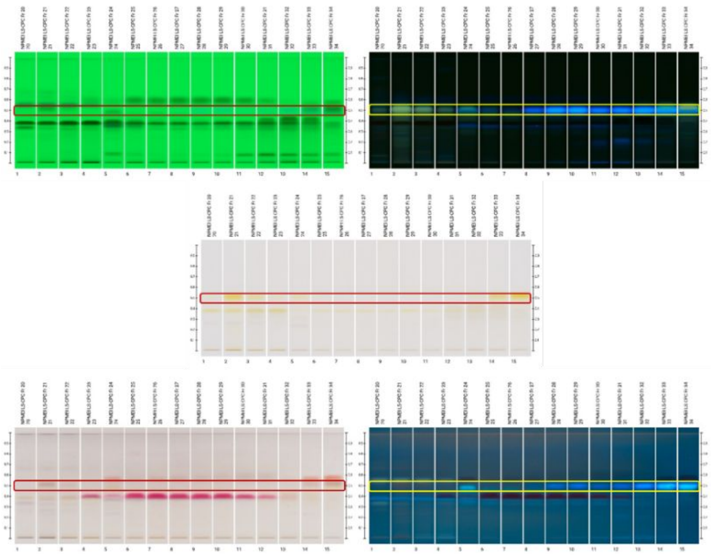

Figure S2. Chromatograms of ArtExtr fractions Fr20-34, where spot 23 is highlighted in NP. The chromatogram order is 254 nm, 366 nm, visible light before derivatization, visible light after spraying with sulfuric vanillin reagent and 366 nm after derivatization.

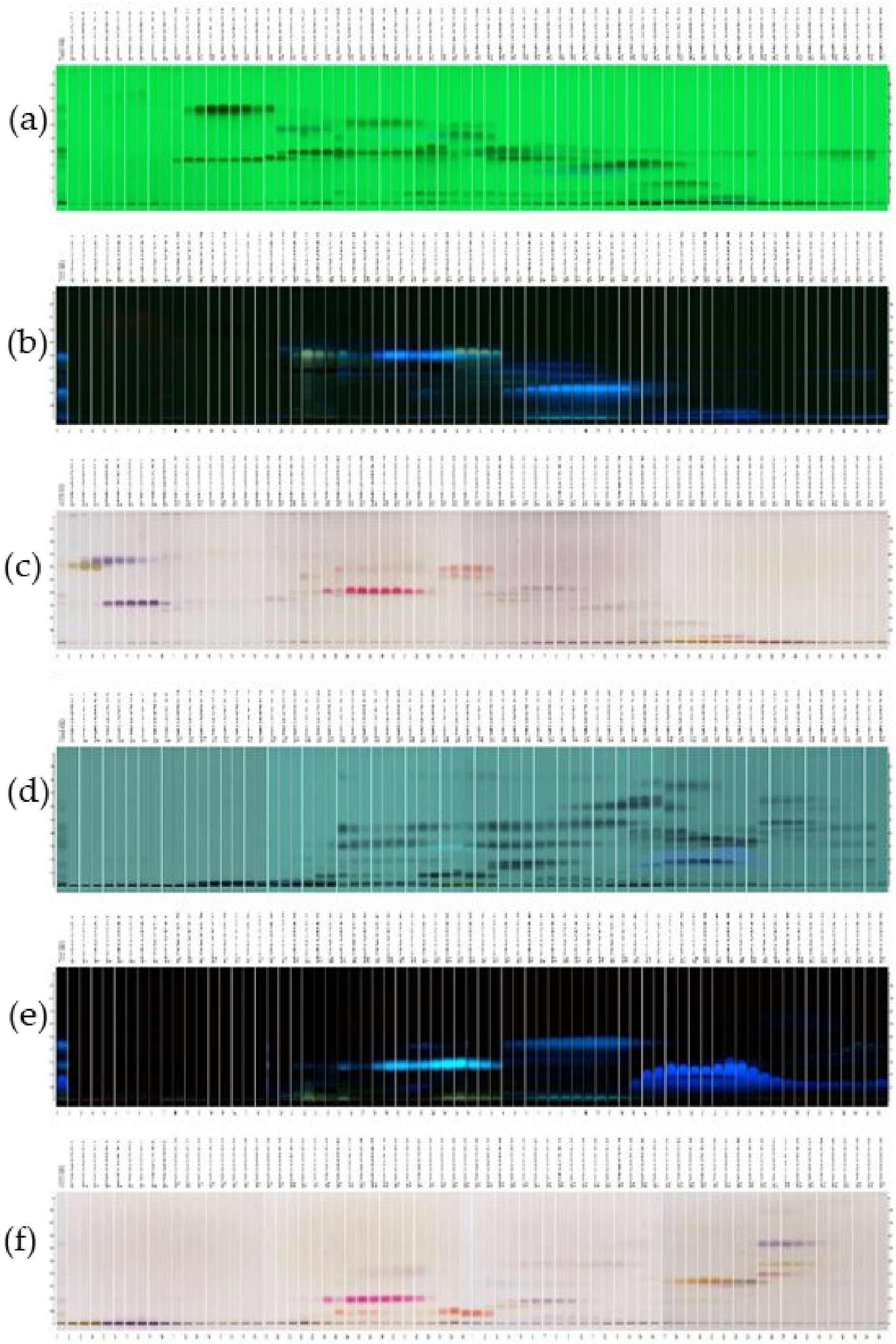

Figure S3. HPTLC chromatograms of FCPC fractions of the ArtExtr. (a) NP at 254 nm; (b) NP at 366 nm; (c) NP in visible light after spraying with sulfuric vanillin reagent; (d) RP at 254 nm; (e)

RP at 366 nm and (f) RP in visible light after derivatization with sulfuric vanillin reagent. Figure reproduced from Amountzias *et al.* (manuscript reference 41) with permission.

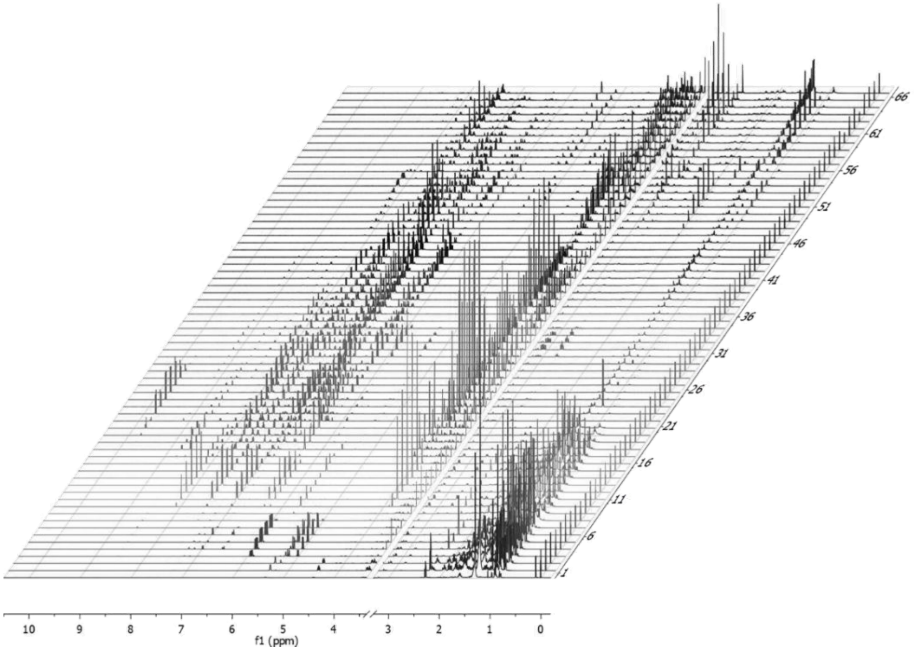

Figure S4. ^{1}H NMR spectra of the ArtExtr fractions. The region 3.38-3.31 ppm corresponding to the methanol-d_4 peak has been excluded from the spectra for better visualization. Figure reproduced from Amountzias *et al.* (manuscript reference 41) with permission.

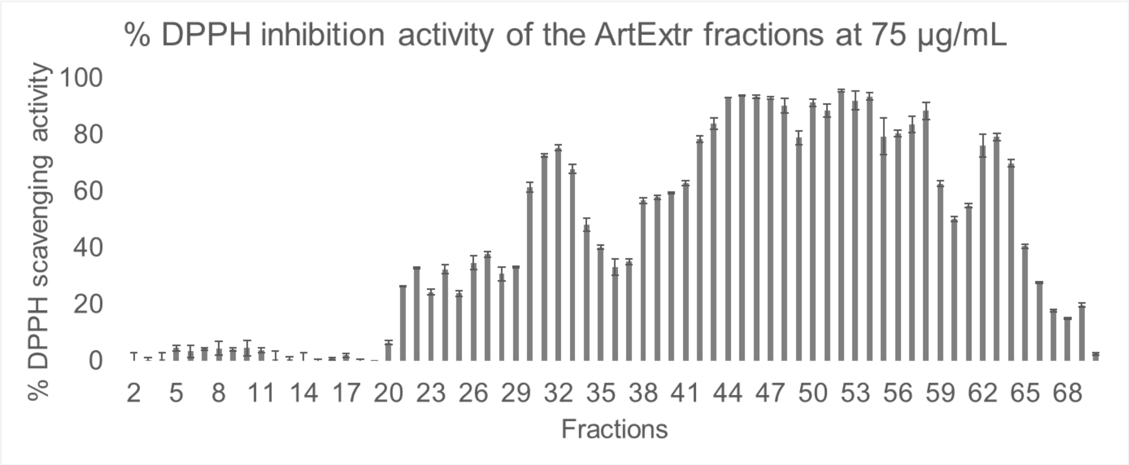

Figure S5. % DPPH scavenging activity of all the fractions of the ArtExtr at 75 μg/mL. Inhibition is expressed as mean ± SD (N=3). Figure reproduced from Amountzias *et al.* (manuscript reference 41) with permission.

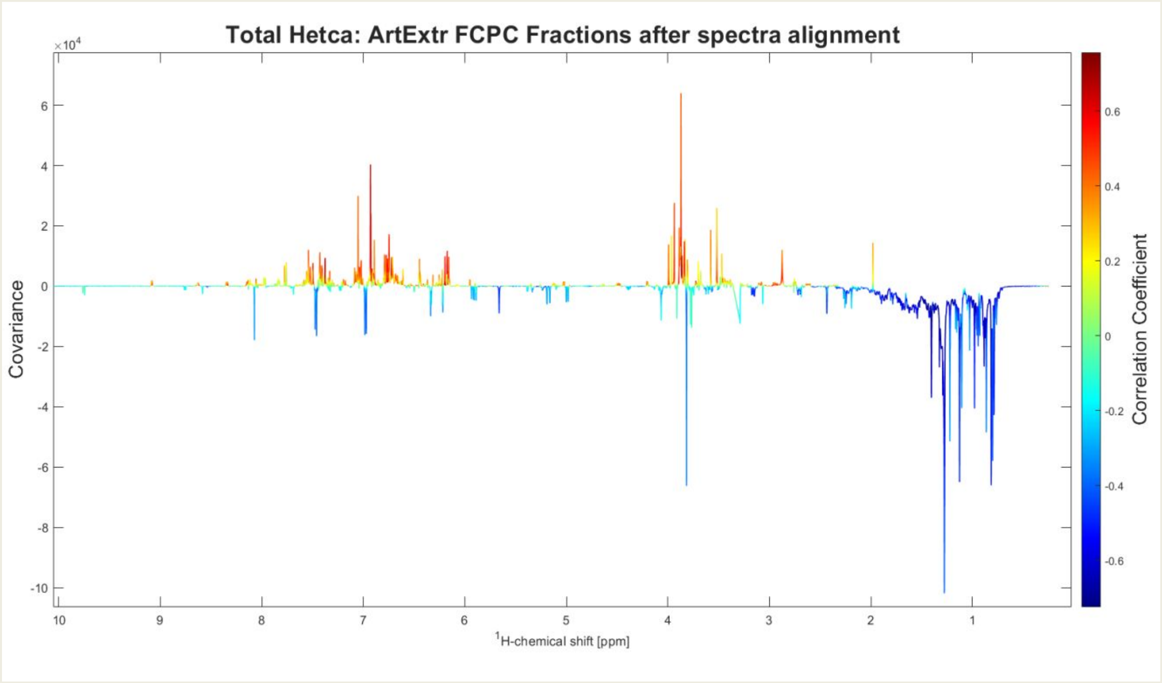

Figure S6. Total HetCA plot obtained from covariance of NMR data with the biological activity of ArtExtr fractions against DPPH, after spectral alignment. Figure reproduced with modifications from Amountzias *et al.* (manuscript reference 21) with permission.

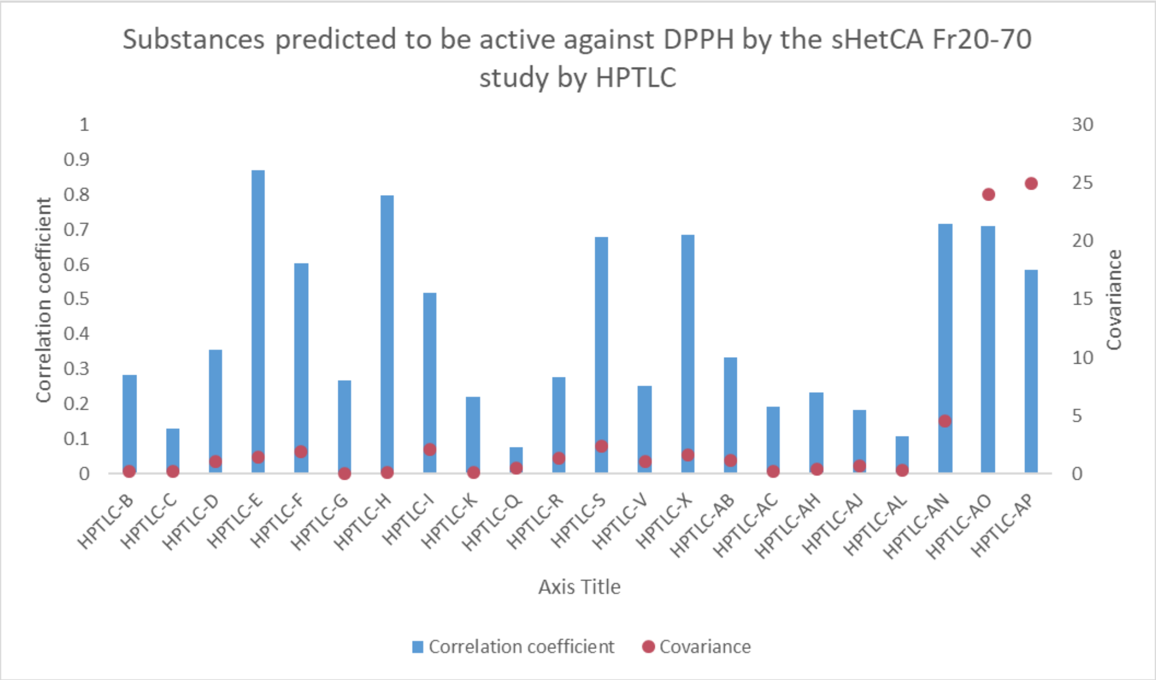

Figure S7. Substances predicted as active from the HPTLC-sHetCA Fr20-70 study.

Table S6. Selected peaks that showed high correlation and covariance with the activity against DPPH in the NMR-Total HetCA plot and that were used as driver peaks for the application of STOCSY.

Chemical shift (ppm)	Multiplicity*	Coupling constant (Hz)
9.09	d	1.4
7.67	d	2.0
7.55	s	
7.42	d	2.0
7.06	s	
6.62	s	
6.46	t	2.3
6.45	d	2.1

6.38	d	2.1
6.33	d	15.9
6.20	d	15.9
6.17	d	9.4
6.09	m	
5.14	dd	9.1/3.8
3.97	s	

*s: singlet, d: doublet, t: triplet, dd: doublet of doublets, m: multiple.

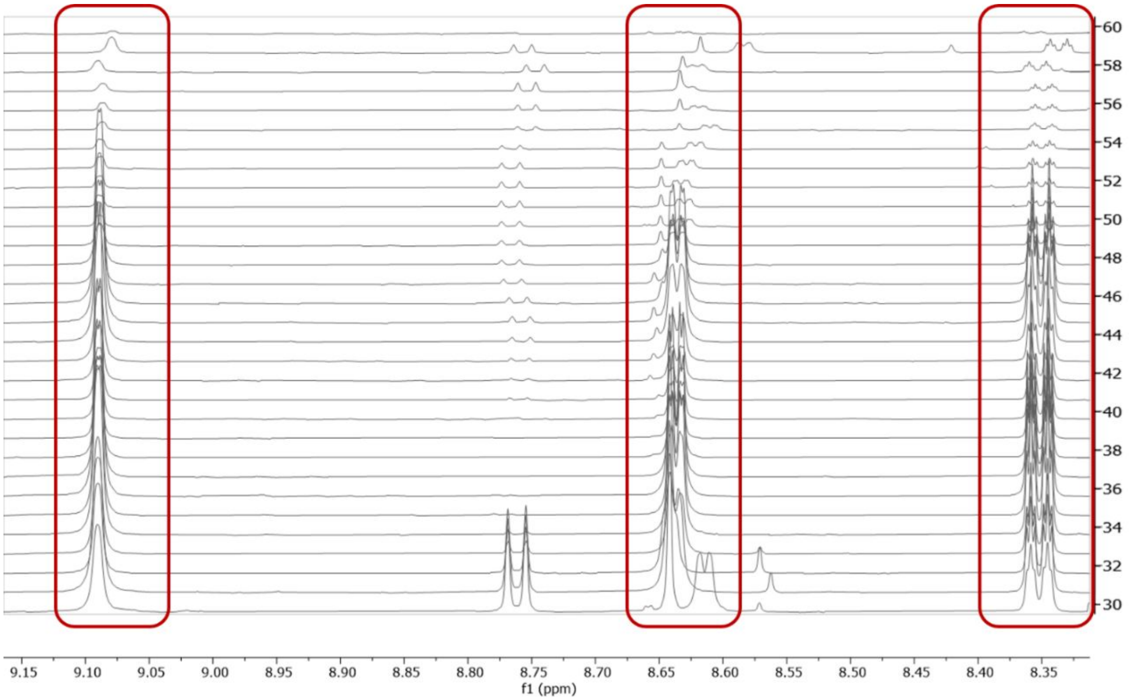

Figure S8. ^{1}H NMR spectra of the ArtExtr fractions Fr30-60 (9.15-8.30 ppm) where the highlighted peaks from the STOCSY pseudospectrum are presented.

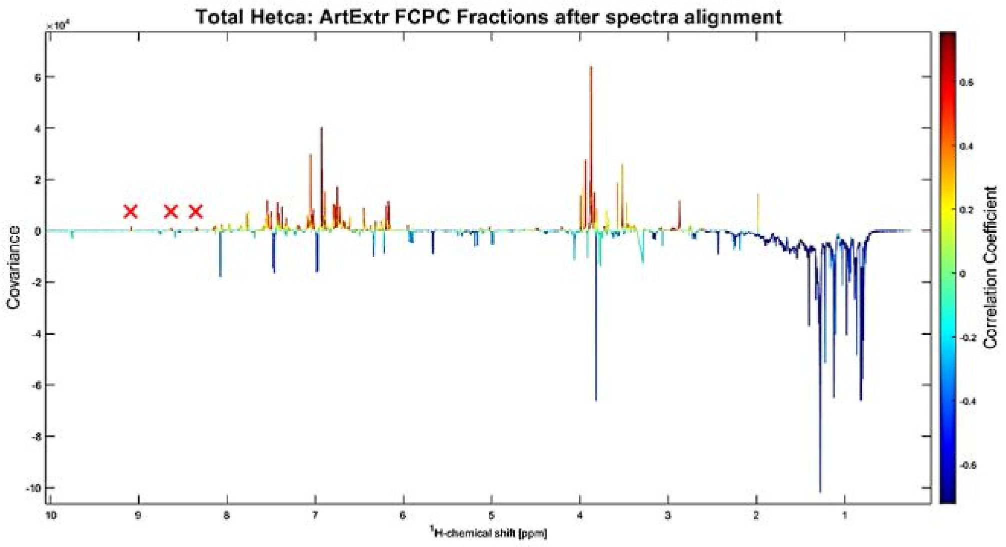

Figure S9. Total HetCA plot where the two peaks related to the driver peak at 9.09 ppm are marked with red Xs and excluded from the identification process.

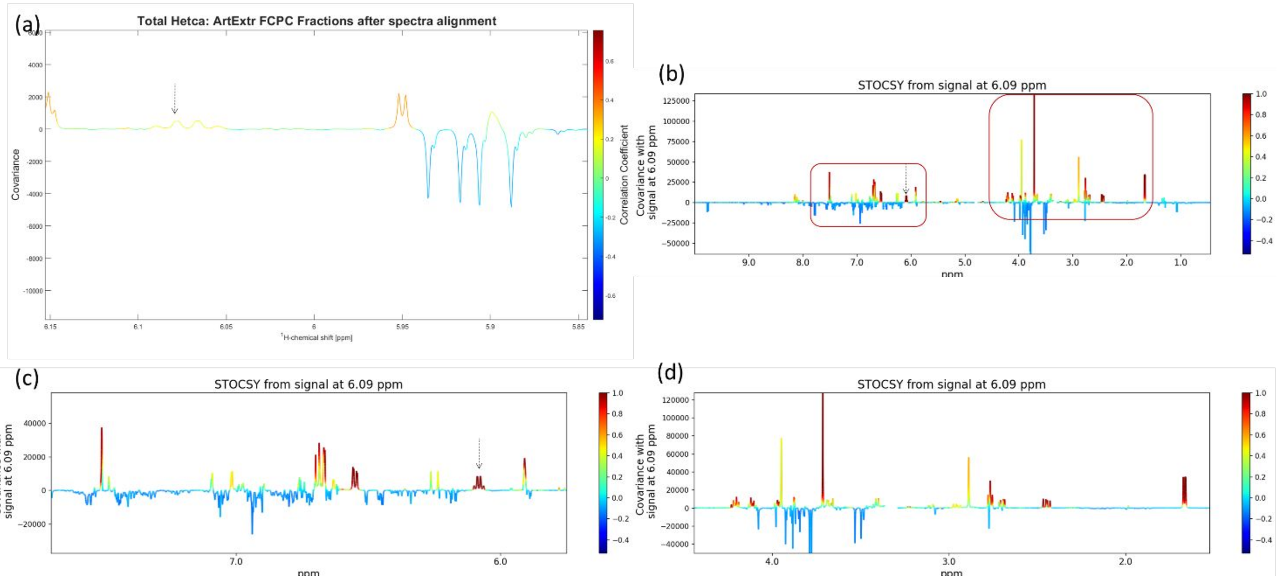

Figure S10. (a) Region of the Total HetCA plot (6.15-5.85 ppm); (b) STOCSY pseudospectrum from the signal at 6.09 ppm in the ArtExtr fractions Fr20-70 (10.00-0.80 ppm) and zoomed regions (c: 7.65-5.85 and d: 4.40-1.60 ppm).

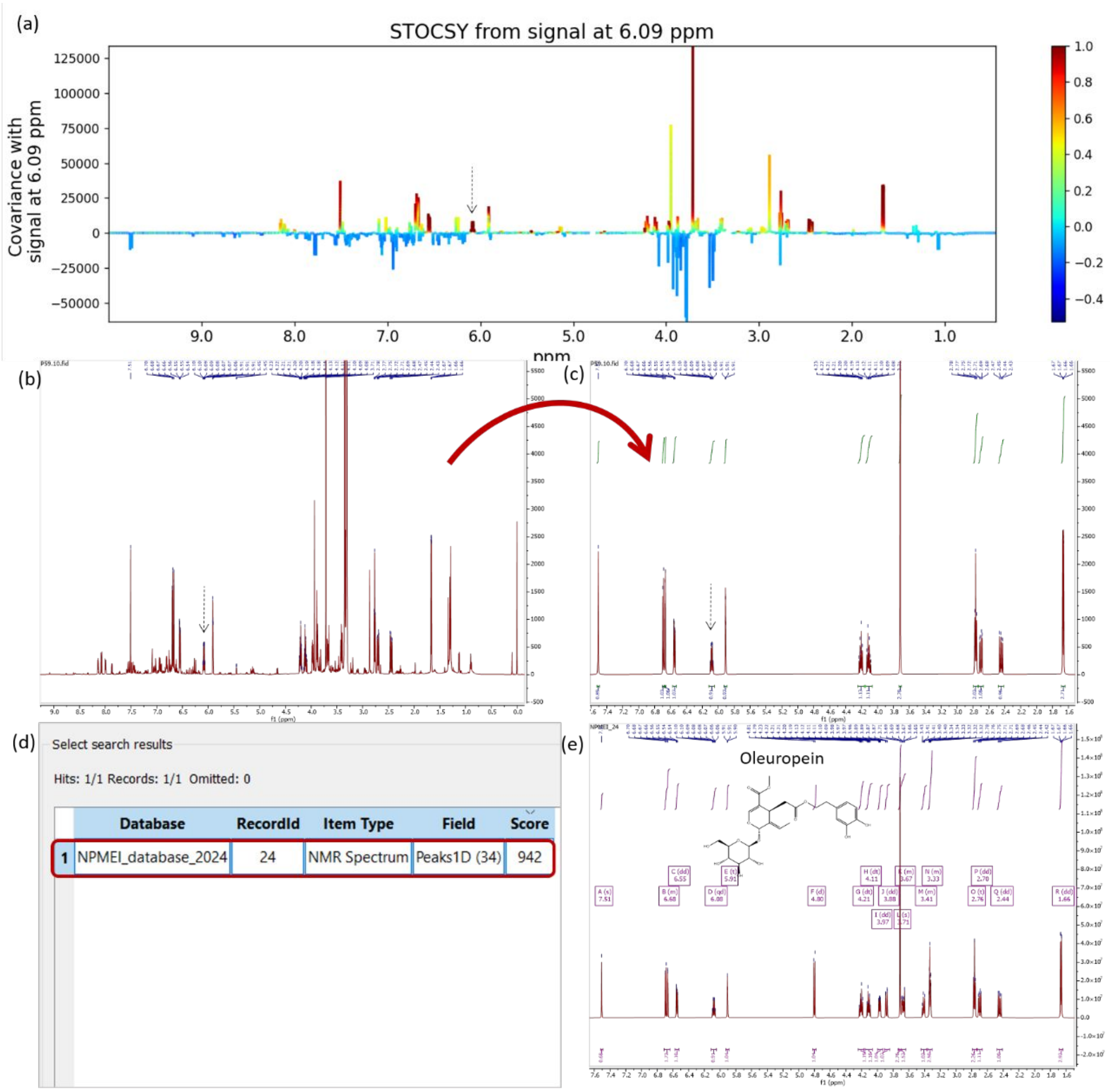

Figure S11. (a) STOCSY pseudospectrum from the signal at 6.09 ppm in the ArtExtr fractions Fr20-70 (10.00-0.80 ppm); (b) ^{1}H NMR spectrum of the fraction Fr59 (9.50-0.00 ppm); (c) selection of STOCSY resulting peaks and spectral depletion (7.60-1.50 ppm); (d) NMR database results, and (e) ^{1}H NMR spectrum of standard oleuropein (7.60-1.50 ppm).

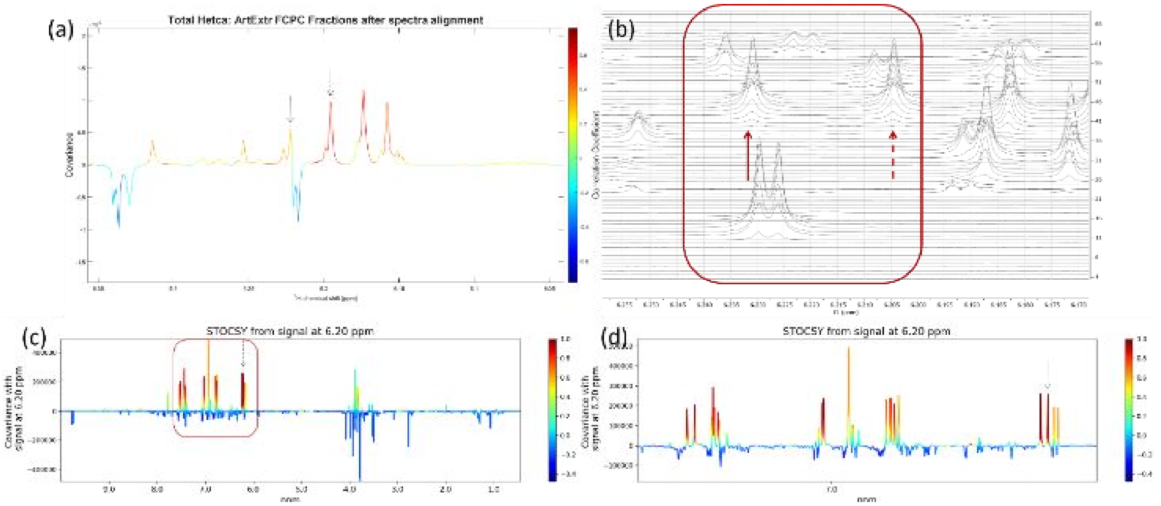

Figure S12. (a) NMR-Total HetCA plot region (6.55-6.25 ppm), where the black dashed arrow shows the selected component, while the black arrow shows the second component of the peak of the *trans* double bond; (ii) stack plot of the spectra ^{1}H NMR of the ArtExtr fractions (Fr02-70), where the red dashed arrow marks the selected component of the trans double bond peak, while the red arrow marks the second component of the trans double bond peak; (c) STOCSY pseudospectrum from the signal at 6.33 ppm in the Fr20-70 fractions of ArtExtr (10.00-0.80 ppm) and zoomed regions (d: 8.00-5.95 and e: 4.00-3.35 ppm).

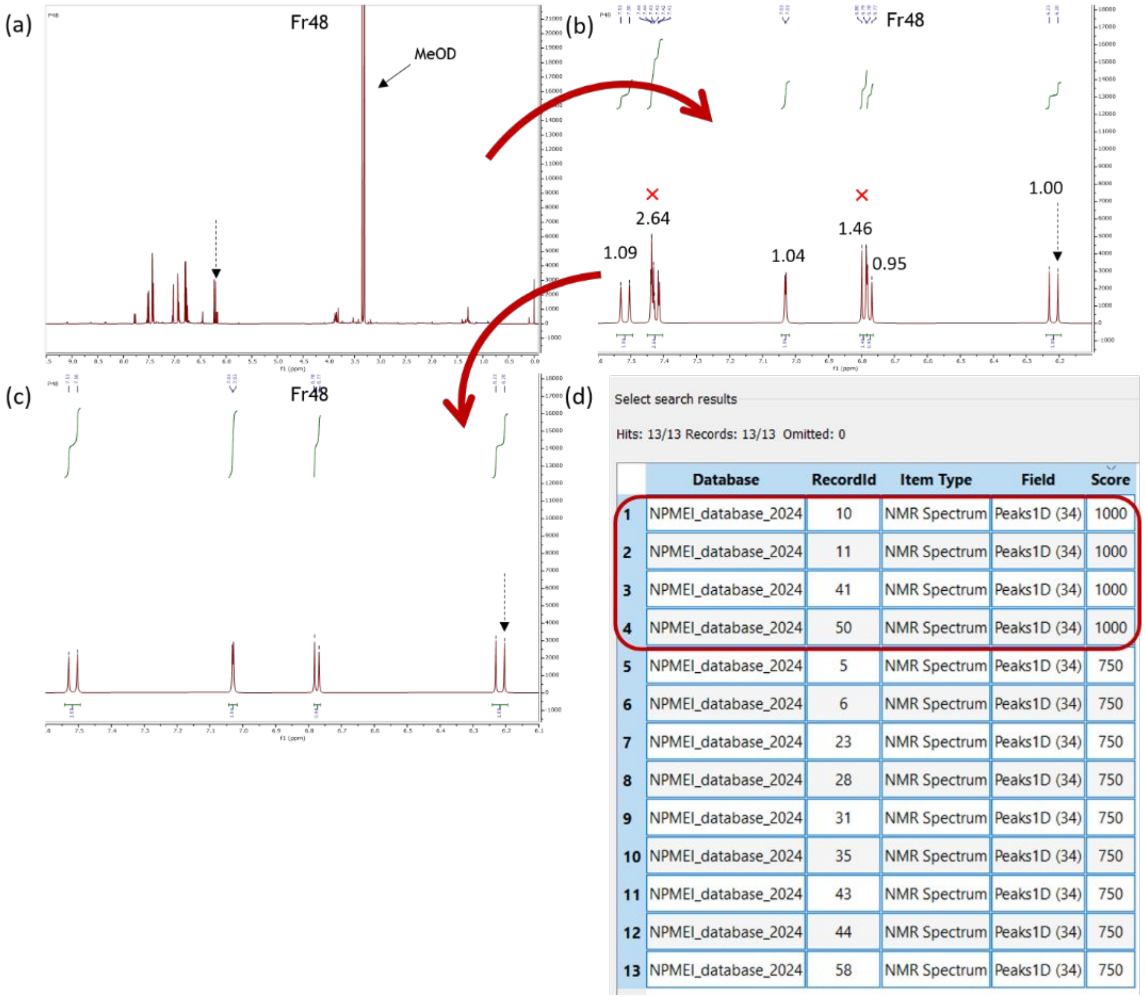

	Database	RecordId	Item Type	Field	Score
1	NPMEI_database_2024	10	NMR Spectrum	Peaks1D (34)	1000
2	NPMEI_database_2024	11	NMR Spectrum	Peaks1D (34)	1000
3	NPMEI_database_2024	41	NMR Spectrum	Peaks1D (34)	1000
4	NPMEI_database_2024	50	NMR Spectrum	Peaks1D (34)	1000
5	NPMEI_database_2024	5	NMR Spectrum	Peaks1D (34)	750
6	NPMEI_database_2024	6	NMR Spectrum	Peaks1D (34)	750
7	NPMEI_database_2024	23	NMR Spectrum	Peaks1D (34)	750
8	NPMEI_database_2024	28	NMR Spectrum	Peaks1D (34)	750
9	NPMEI_database_2024	31	NMR Spectrum	Peaks1D (34)	750
10	NPMEI_database_2024	35	NMR Spectrum	Peaks1D (34)	750
11	NPMEI_database_2024	43	NMR Spectrum	Peaks1D (34)	750
12	NPMEI_database_2024	44	NMR Spectrum	Peaks1D (34)	750
13	NPMEI_database_2024	58	NMR Spectrum	Peaks1D (34)	750

Figure S13. (a) ^{1}H NMR spectrum of the fraction Fr48 (8.00-0.00 ppm); (b) selection of STOCSY resulting peaks and spectral depletion (7.60-6.15 ppm); (c) remaining peaks (7.60-6.15 ppm) and (d) NMR database results.

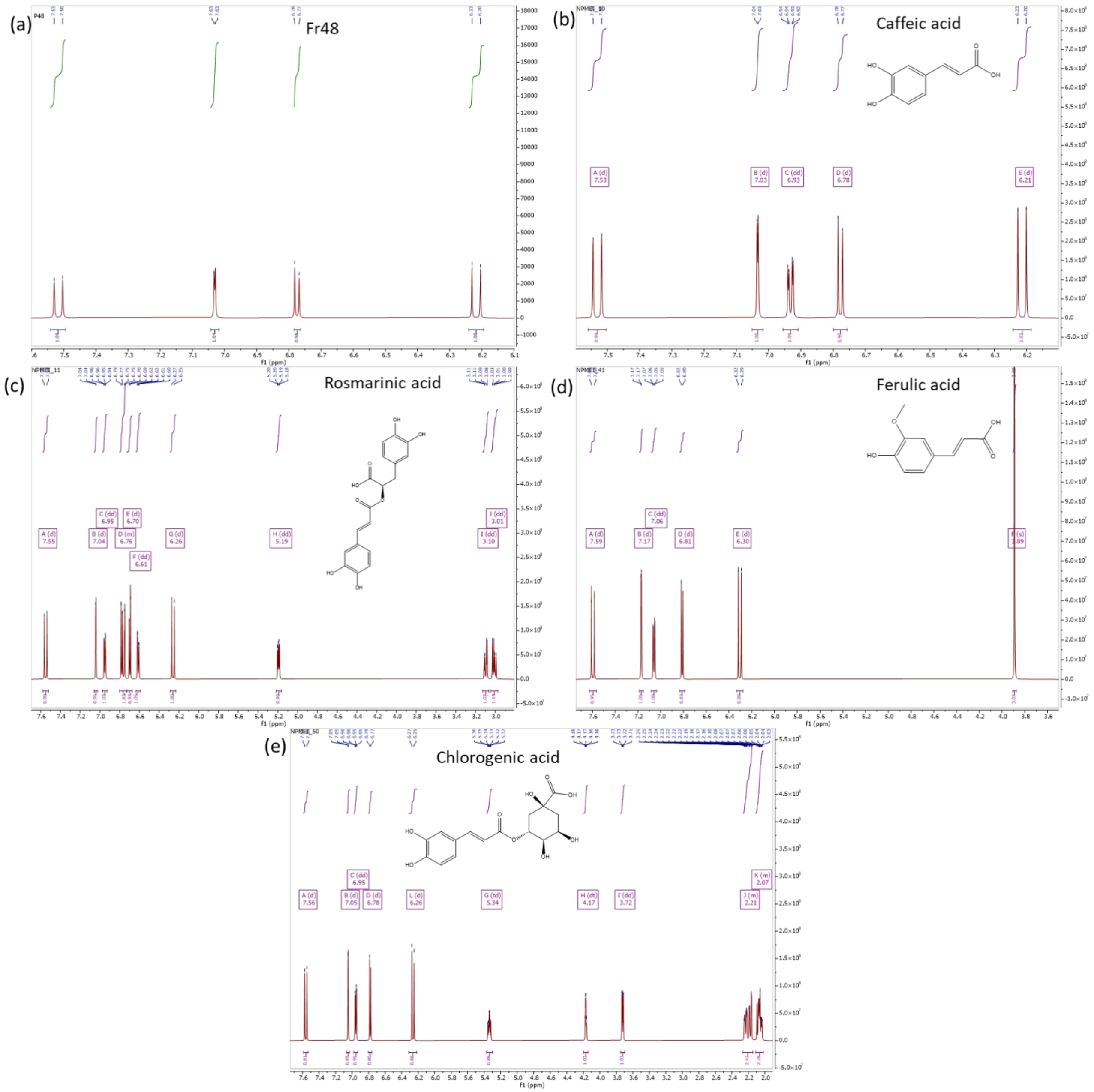

Figure S14. (a) Spectral region (7.60-6.15 ppm) of the fraction Fr48 where the selected peaks are shown; (b) spectral region of the standard caffeic acid (7.60-6.15 ppm); (c) spectral region of the standard rosmarinic acid (7.60-2.95 ppm); (d) spectral region of the standard ferulic acid (7.65-3.60 ppm) and (e) spectral region of the standard chlorogenic acid (7.65-2.00 ppm).

Table S7. Compounds identified via the STOCSY algorithm and the NMR database.

Identified compound	Characteristic peak in the ^{1}H NMR spectrum	Score (/1000)
Nicotinic acid	9.09	1000
Rutin	7.67	1000
Ellagic acid (dihydrate)	7.55	1000
Protocatechic acid	7.42	1000
Gallic acid	7.06	1000
Baicalein	6.62	884
3,5-Dihydroxybenzoic acid	6.46	1000
Resveratrol	6.45	882
Sinapic acid	6.33	1000
Caffeic acid	6.20	1000
6,7-Dihydroxycoumarin	6.17	1000
Oleuropein	6.09	942
Rosmarinic acid	5.14	1000
Caffeine	3.97	1000
**Partially identified compound**	**Characteristic peak in the ^{1}H NMR spectrum**	**Score (/1000)**
Quercetin-type flavonol glycoside	6.38	-

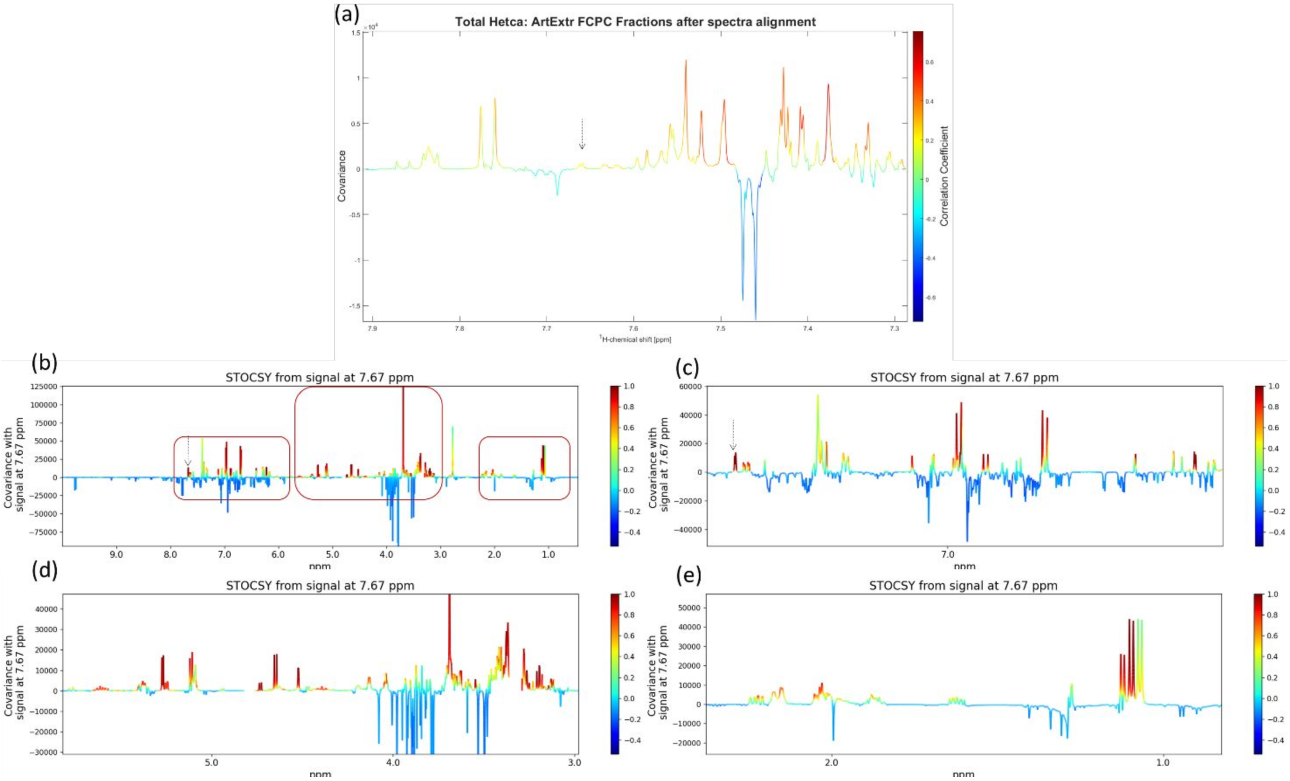

Figure S15. (a) Region of Total HetCA plot (7.90-7.29 ppm); (b) STOCSY pseudospectrum from the signal at 7.67 ppm in the fractions Fr20-70 of ArtExtr (10.00-0.80 ppm) and zoomed areas (c: 7.75-6.15; d: 5.80-3.00 and f: 2.40-0.80 ppm).

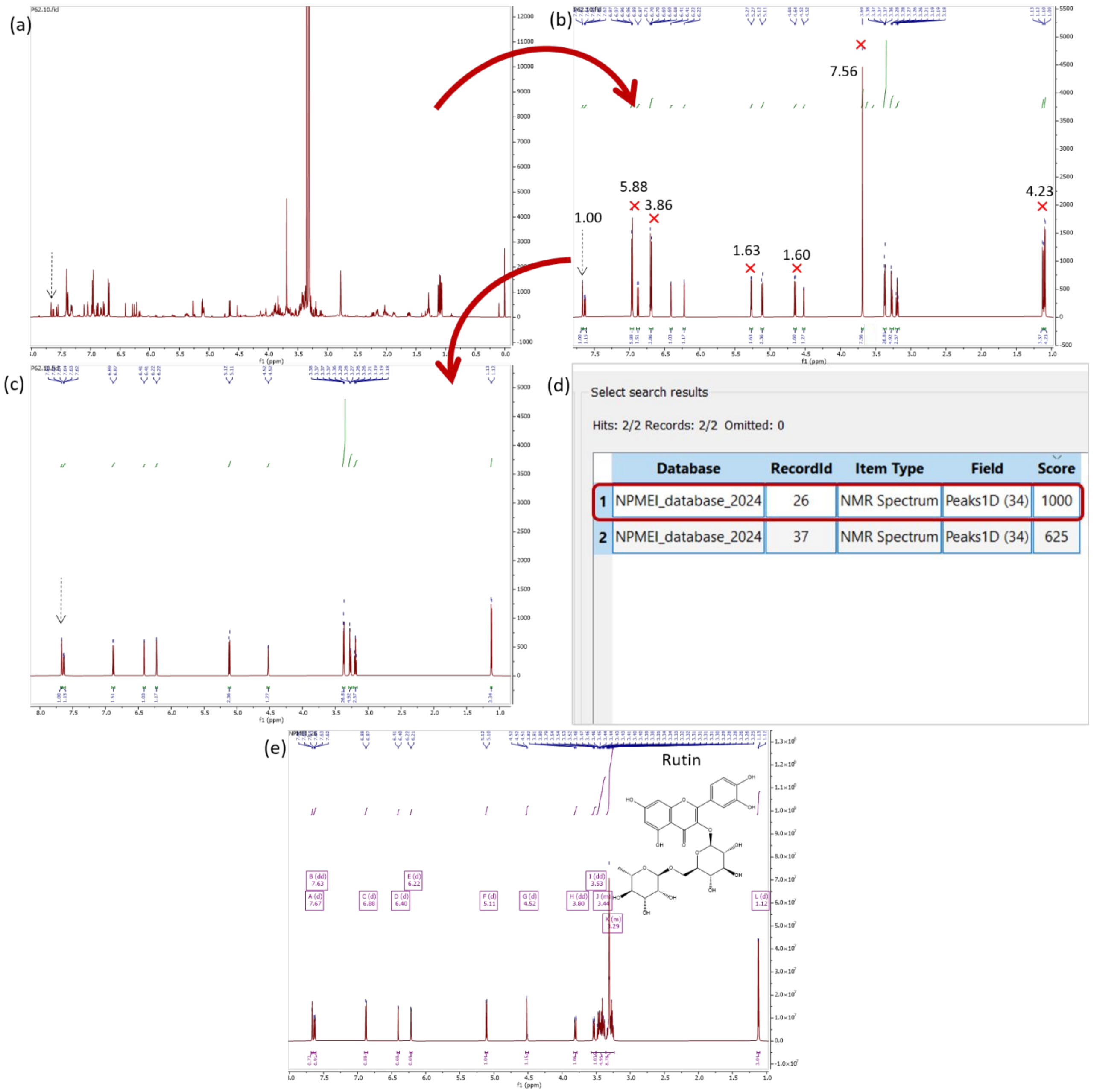

Figure S16. (a) ^{1}H NMR spectrum of the fraction Fr62 (8.00-0.00 ppm); (b) selection of STOCSY resulting peaks and spectral depletion (8.00-1.00 ppm); (c) remaining peaks (8.00-1.00 ppm); (d) NMR library results and (e) ^{1}H NMR spectrum region of standard rutin (8.00-1.00 ppm).

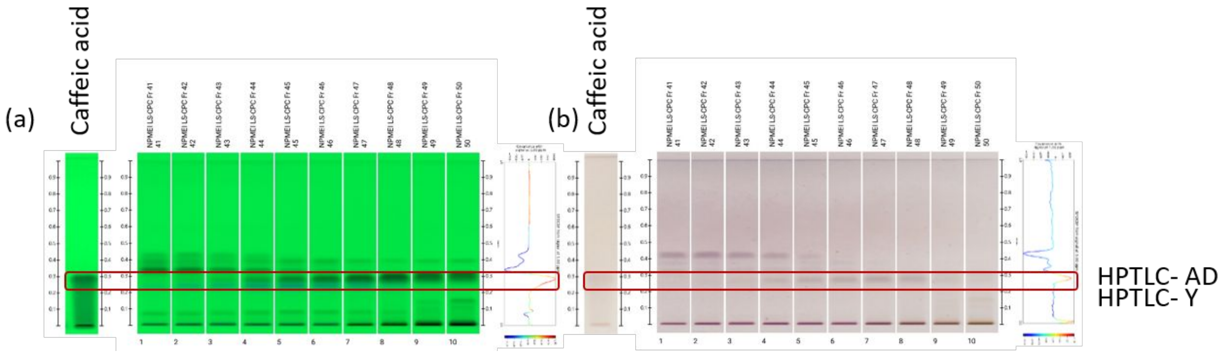

Figure S17. HPTLC comparison of standard caffeic acid with the ArtExtr fractions Fr41-50 and SH-SCY between NMR and HPTLC for the driver peak at 6.20 ppm in NP (a) at 254 nm and (b) in visible light after derivatization with sulfuric vanillin reagent.

Partial identification of compounds with high covariance in their concentrations via combination of NMR and HPTLC – Driver peak at 6.38 ppm

The peak at 6.38 ppm (d, $J = 2.1$ Hz) demonstrated high correlation and covariance with multiple peaks in the STOCSY pseudospectrum, while the integration values between them were correspondent (Figure S18 and S19). From the study of the ^{1}H NMR spectra of the fractions Fr51-58, it is evident that the signals of the highly correlated STOCSY peaks correspond to more than one compounds with high covariance in their concentrations. One of these compounds is identified as a quercetin-type flavonoid, containing at least one sugar moiety. The reasoning is as follows: (a) peaks at 6.38 ppm and 6.21 ppm (d, $J = 2.0$ Hz) corresponding to H8 and H6, respectively; (b)the classical ABX coupling system in the three substituted ring for H6' (7.34 ppm, d, $J = 2.0$ Hz), H2' (7.31 ppm, dd, $J = 8.0/2.0$ Hz) and H3' (6.91 ppm, d, $J = 8.0$ Hz) corresponding to the B-ring of the flavonoid and (c) anomeric proton peaks of sugars at 5.36 ppm and 5.03 ppm. The selection of these specific peaks was based on the presence of the peaks at 6.38 ppm and 6.21 ppm, which suggest the existence of a flavonoid, in combination with some peaks located in the 7.50–6.65 ppm region of the ^{1}H NMR spectrum. The peaks in (b) were chosen because, as shown in Figure S20, where the selected peaks that exhibited the highest correlation with the peak at 6.38 ppm through STOCSY are displayed, the peaks at 7.41 ppm and 7.19 ppm (marked in a red frame) appear to be correlated and correspond to the same compound. However, their coupling constant ($J = 11.0$ Hz) excludes them as peaks of the B ring of the flavonoid. In the same spectrum, peaks at 7.34 ppm (d, $J = 2.0$ Hz) and 7.31 ppm (dd, $J = 8.0/2.0$ Hz) are observed (Figure S20, marked in a black frame), which correspond to a trisubstituted aromatic ring. Among the two remaining double peaks with a coupling constant of $J = 8.0$ Hz, the peak at 6.91 ppm appears to complete the ABX system of the flavonoid's B ring, since the peak at 6.68 ppm (Figure S20, marked in a yellow frame) is too shielded to belong to this particular system. Since no other peaks corresponding to

flavanones, flavanones, etc. were observed, it was concluded that it was a quercetin-type flavonol glycoside.

Since quercetin-type flavonols are known to exhibit high DPPH scavenging activity[3], an attempt was made to locate the flavonoid in HPTLC. Figure S21 depicts the SH-SCY of the driver peak (6.38 ppm) with the HPTLC densitograms of the fractions Fr51-58 where three correlated spots were evident. However, one was excluded after derivatization with SVR, as it did not show quercetin-type flavonoid compatible staining (HPTLC-AI, Rf 0.35, orange color). Between the other two spots, HPTLC-AH is not clearly visible at 366 nm, while it fluoresces with a light blue color after derivatization, a common characteristic of flavonols[4], contrary to the spot HPTLC-AG, which does not fluoresce. This observation is consistent with the SH-SCY between NMR and HPTLC at 366 nm after derivatization, where a correlation with the spot HPTLC-AH is evident (Figure S22). Given these observations, the quercetin-type flavonoid corresponds to the HPTLC-AH spot. However, it is not possible to identify the two compounds that exhibit a high covariance in their concentration with the flavonoid.

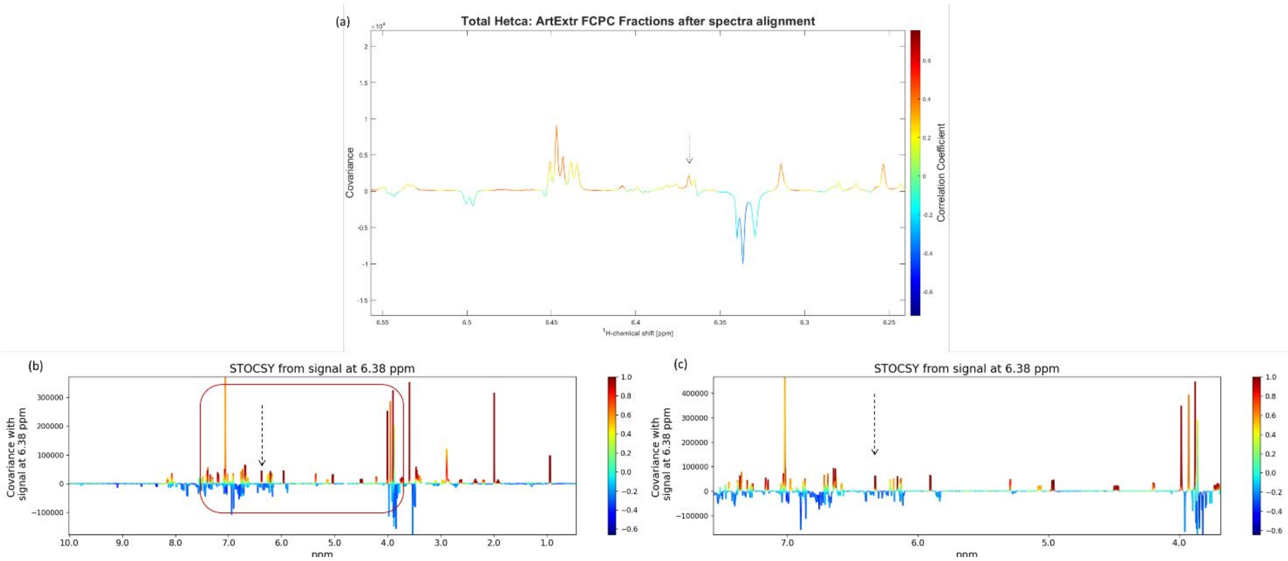

Figure S18. (a) Region of Total HetCA plot (6.55-6.25 ppm); (b) STOCSY pseudospectrum from the signal at 6.38 ppm in the fractions Fr20-70 of ArtExtr (10.00-0.80 ppm) and (c) zoomed area (7.50-3.80 ppm).

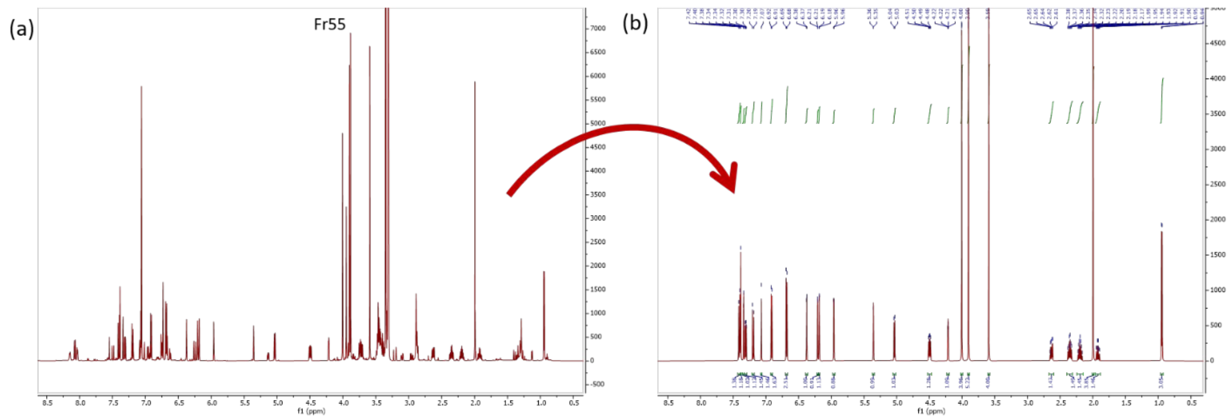

Figure S19. (a) ^{1}H NMR spectrum of the fraction Fr55 (8.50-0.50 ppm) and (b) selection of STOCSY resulting peaks and spectral depletion (8.50-0.50 ppm).

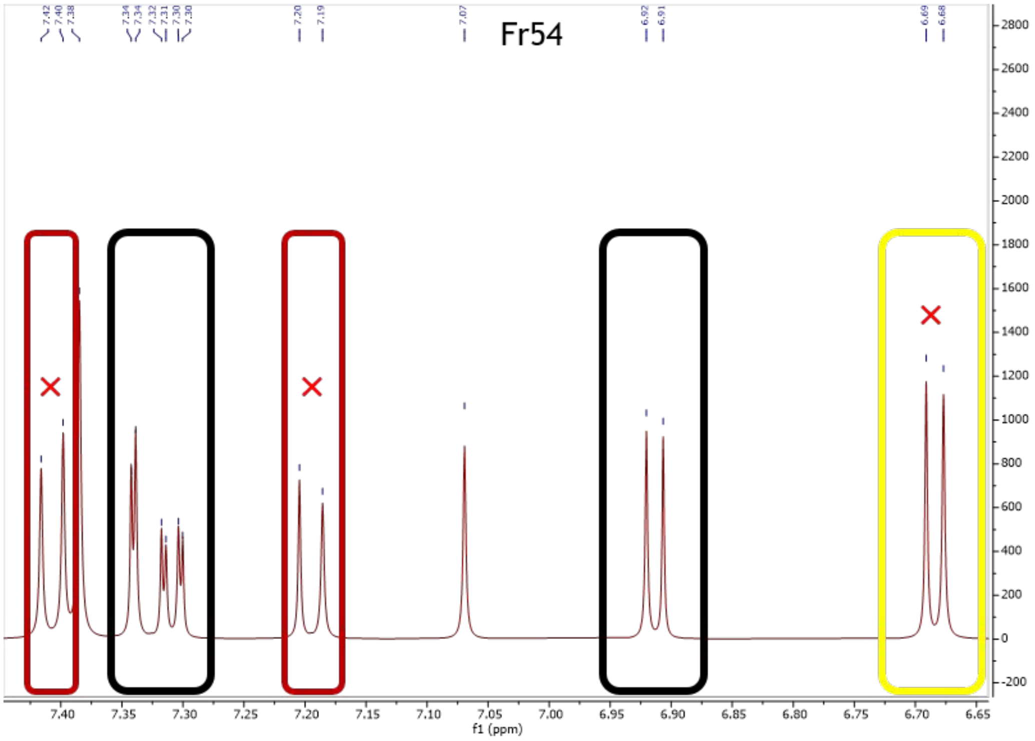

Figure S20. Selected STOCSY peaks from the signal at 6.38 ppm in the fraction Fr54 ^{1}H NMR spectrum (7.45-6.65 ppm).

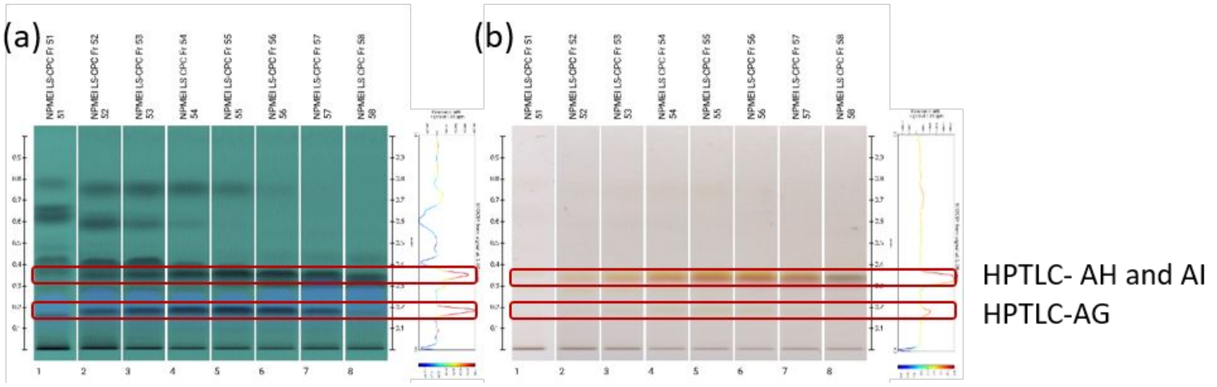

Figure S21. SH-SCY between NMR and HPTLC in the fractions Fr51-58 for the driver peak at 6.38 ppm in RP (a) at 254 nm and (b) in visible light after derivatization with sulfuric vanillin reagent.

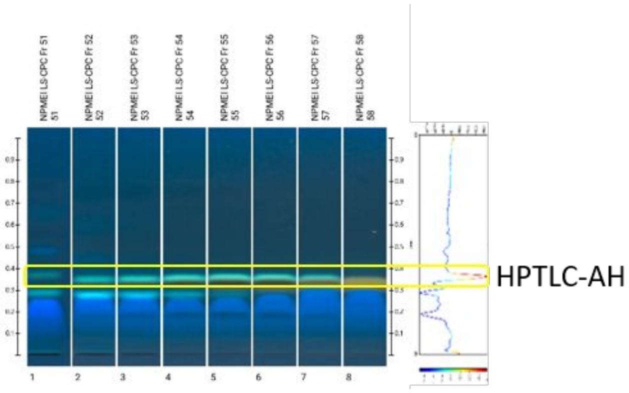

Figure S22. SH-SCY between NMR and HPTLC in the fractions Fr51-58 for the driver peak at 6.38 ppm in RP at 366 nm, after derivatization with sulfuric vanillin reagent.

Table S8. Assignment of NMR-HetCA resulting compounds to HPTLC spots.

Identified compound	Characteristic peak in the ^{1}H NMR spectrum	Assigned HPTLC peak
Rutin	7.67	HPTLC-AO

Protocatechic acid	7.42	HPTLC-AD
Gallic acid	7.06	HPTLC-AF
Resveratrol	6.45	HPTLC-AB
Caffeic acid	6.20	HPTLC-AR
6,7-Dihydroxycoumarin	6.17	HPTLC-Y
Oleuropein	6.09	HPTLC-AL
Rosmarinic acid	5.14	HPTLC-AK
**Partially identified compound**	**Characteristic peak in the ^{1}H NMR spectrum**	**Assigned HPTLC peak**
Quercetin-type flavonol glycoside	6.38	HPTLC-AH

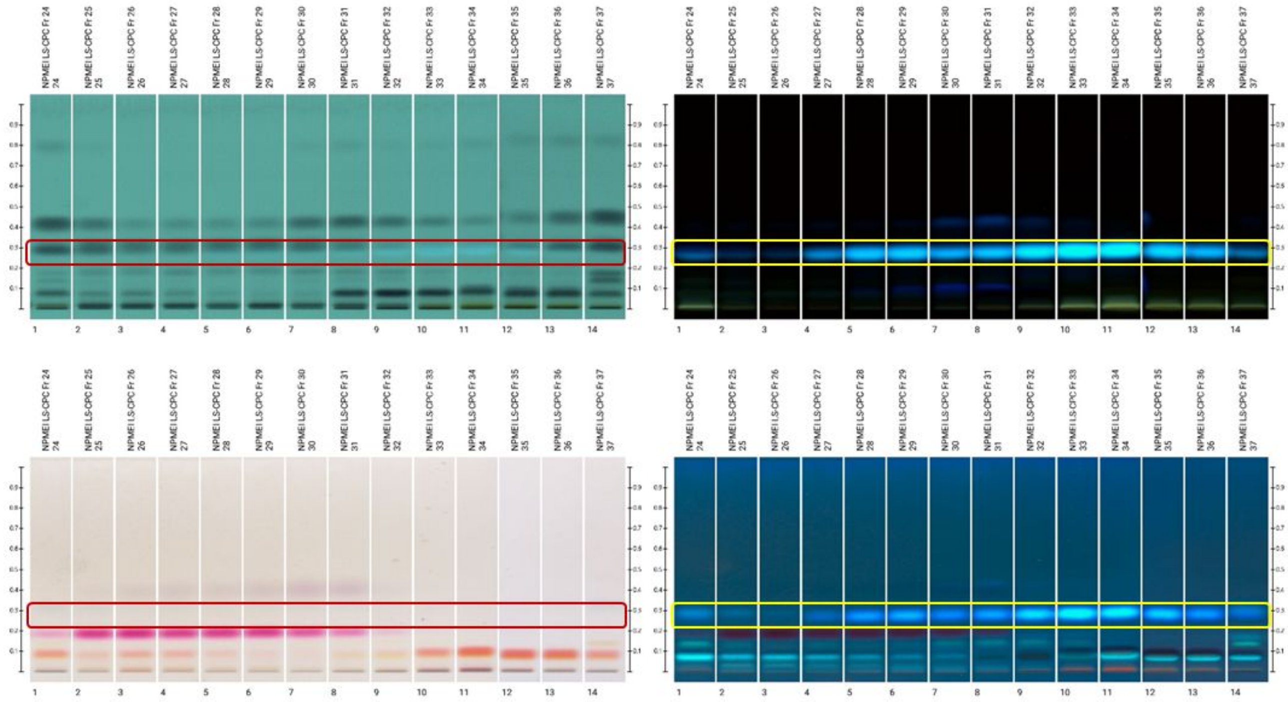

Figure S23. Chromatograms of the ArtExtr fractions Fr24-37, where the spot HPTLC-N in RP is highlighted. The order of the chromatograms is 254 nm, 366 nm, visible light after derivatization with sulfuric vanillin reagent, and 366 nm after derivatization.

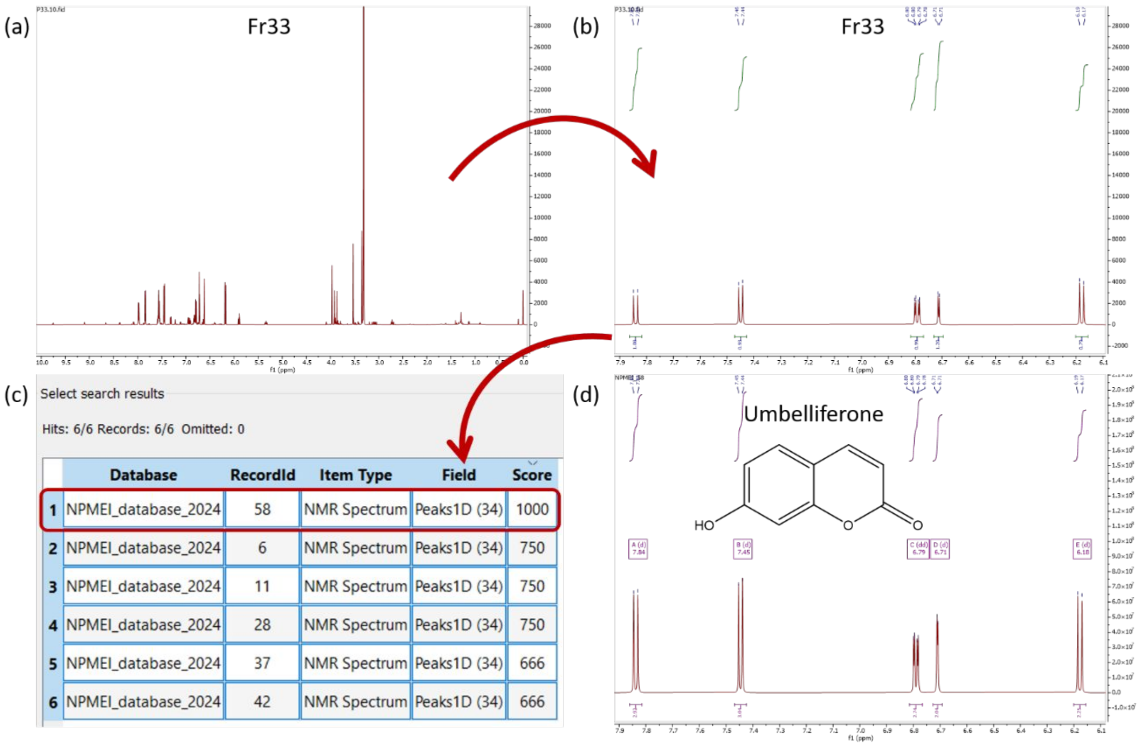

Figure S24. (a) ^{1}H NMR spectrum of the fraction Fr33 (10.00-0.00 ppm); (b) selection of STOCSY resulting peaks (7.90-6.10 ppm); (c) NMR library results and (d) ^{1}H NMR spectrum region of standard umbelliferone (7.90-6.10 ppm).

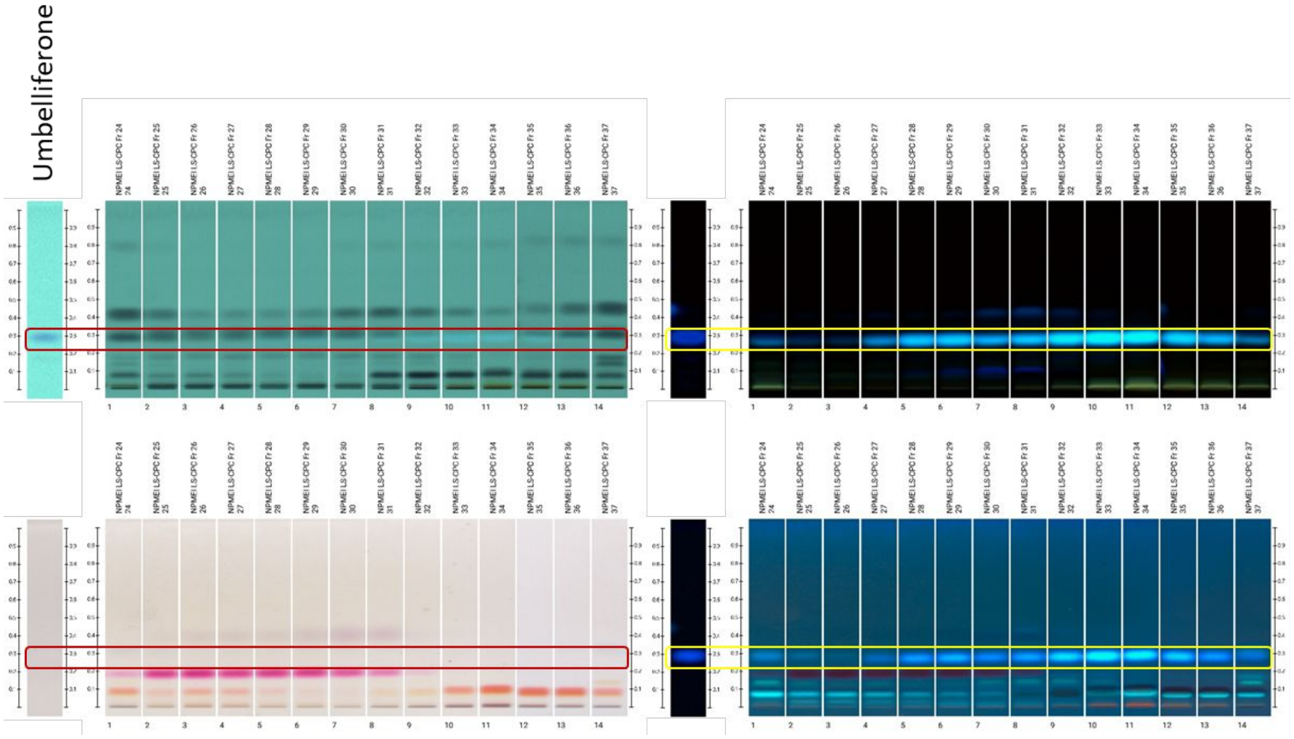

Figure S25. Comparison of the chromatograms of standard umbelliferone with the chromatograms of the fractions Fr24-37 of ArtExtr in RP. The order of the chromatograms is 254 nm, 366 nm, visible light after derivatization with sulfuric vanillin reagent, and 366 nm after derivatization.

Identification via NMR database despite peak misalignment - Spot HPTLC-AJ

The analysis of the fractions Fr49-61 chromatograms for the spot HPTLC-AJ (Rf_{RP} 0.10-0.30) (Figure S26) showed absorption at 254 nm, with a blue appearance in color. Additionally, it fluoresced at 366 nm both before and after derivatization, yet did not react with SVR. Based on these observations, it is likely that the corresponding compound contains π-conjugated systems.

Figure S27 presents the results of the SH-SCY analysis between HPTLC and NMR. Peaks that exhibited high correlation with HPTLC-AJ were identified in fractions Fr49-61. From these, the fraction Fr54 ^{1}H NMR spectrum was selected, as the peaks were adequately resolved and there was no significant overlap or shift displacement (Figure S28). After the exclusion of peaks, via spectral depletion, that did not correspond to the same compound according to the integration values, a comparison was made with the standard substances spectra of the database (Figure S29). The results suggested harmine and kaempferol with scores of 785 and 714 out of 1000, respectively. However, upon further examination, the characteristic flavonoid peaks of H8 and H6 did not correspond to the same compound based on integration values, so kaempferol was rejected. As for harmine, there were differences in the chemical shift of the spectral peaks in the Fr54

fraction compared to the standard substance. These differences were likely due to the influence of methanol-d_4, which has slightly acidic characteristics, as well as the presence of other substances in the same fractions, which may also have an acidic nature.[5] These factors likely contributed to the lower score in the database during the identification process. Moreover, due to these reasons, harmine was not initially identified in the ^{1}H NMR spectra. To verify the result, the chromatograms of standard harmine and the ArtExtr fractions Fr49-61 were compared, confirming that the Rf index, absorption, and fluorescence properties of the spot HPTLC-AJ were consistent with those of the standard compound (Figure S30). The observed differences in the Rf values were attributed to the presence of other compounds in the same fractions. Based on these findings, there is a strong likelihood that the compound in question is harmine.

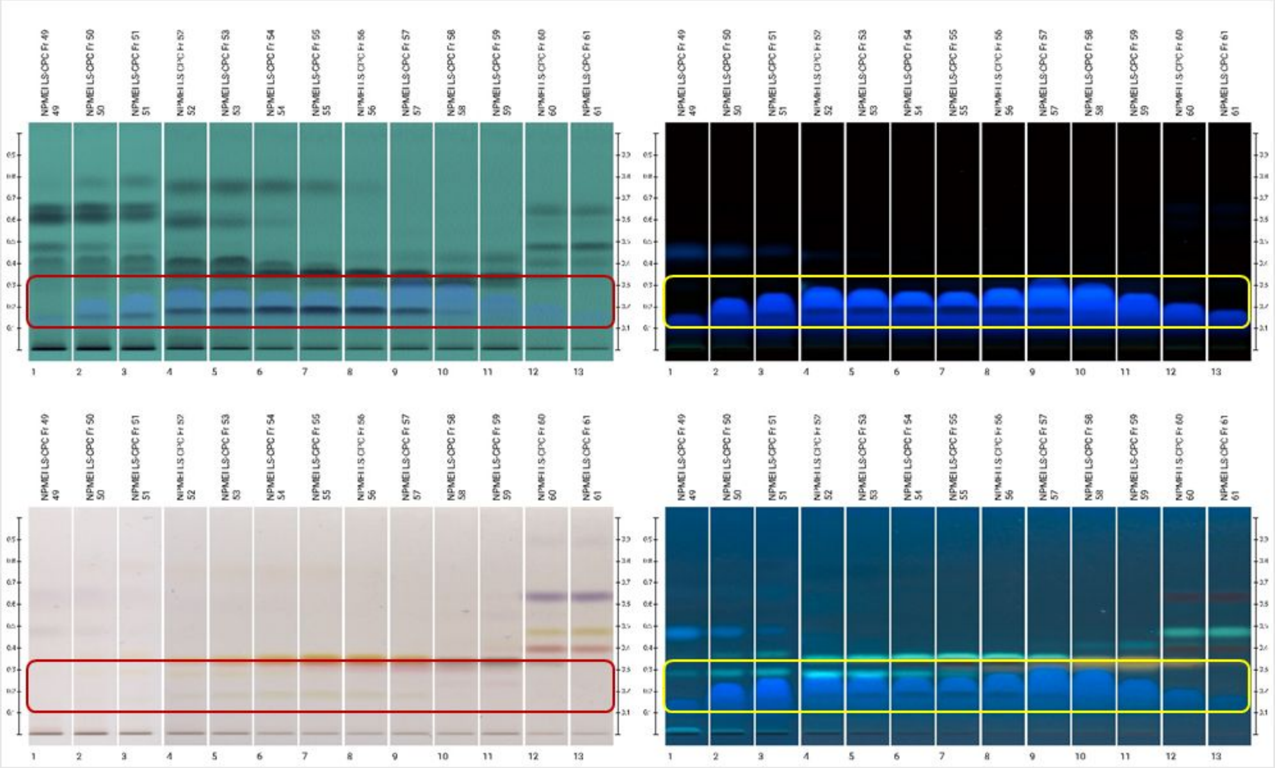

Figure S26. Chromatograms of the ArtExtr fractions Fr49-61, where the spot HPTLC-AJ is highlighted in RP. The chromatograms order is 254 nm, 366 nm, visible light after derivatization with SVR, and 366 nm after derivatization.

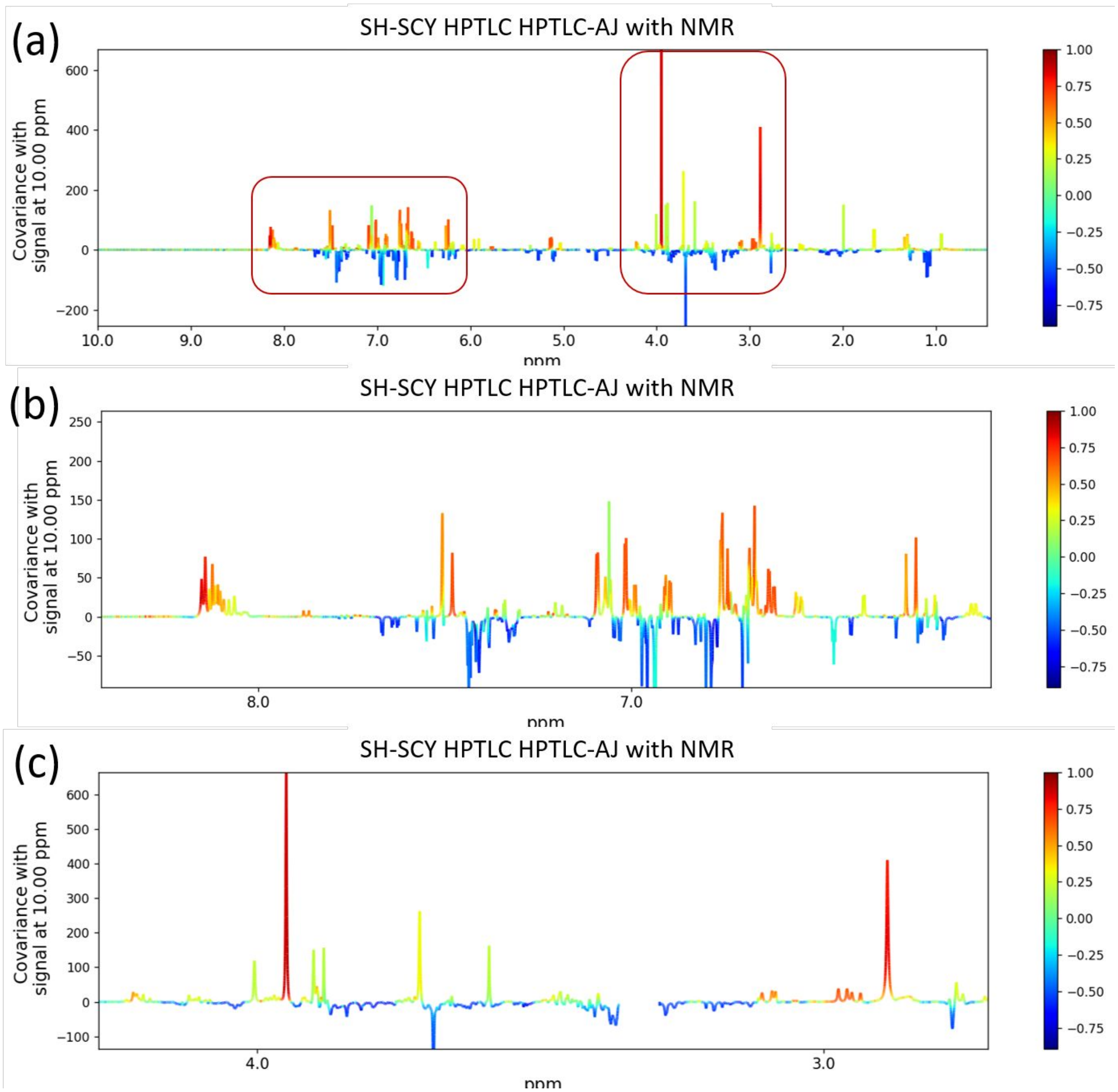

Figure S27. (a) SH-SCY pseudospectrum between HPTLC and NMR in the ArtExtr fractions Fr49-61 (10.00-0.80 ppm) for the spot HPTLC-AJ and zoomed areas (b: 8.40-6.00 and c: 4.30 - 2.70 ppm).

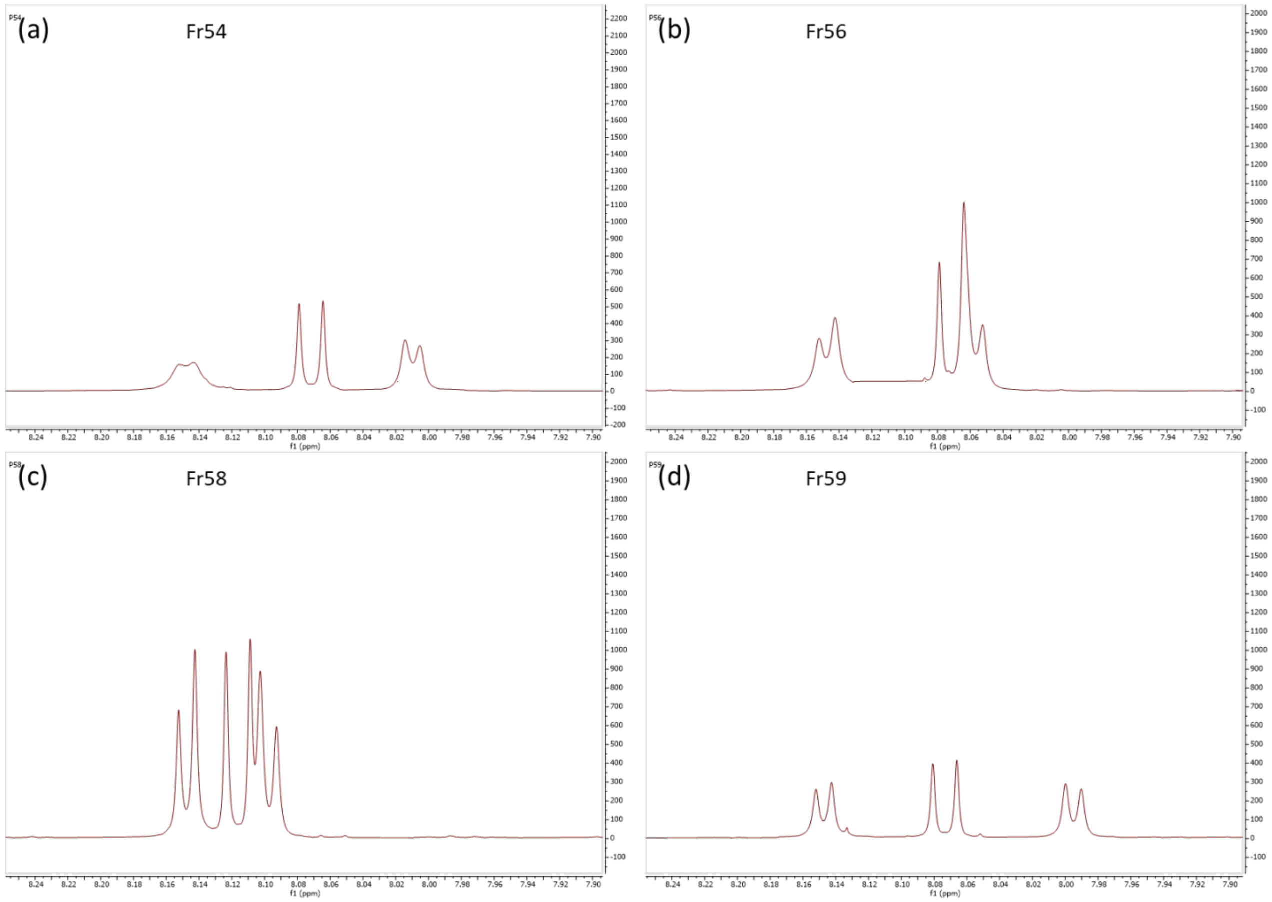

Figure S28. Region of ^{1}H NMR spectra (8.25-7.90 ppm) of ArtExtr fractions (a) Fr54, (b) Fr56, (c) Fr58 and (d) Fr59 depicting the shift displacement of some of the peaks that exhibited a high correlation coefficient with the spot HPTLC-AJ.

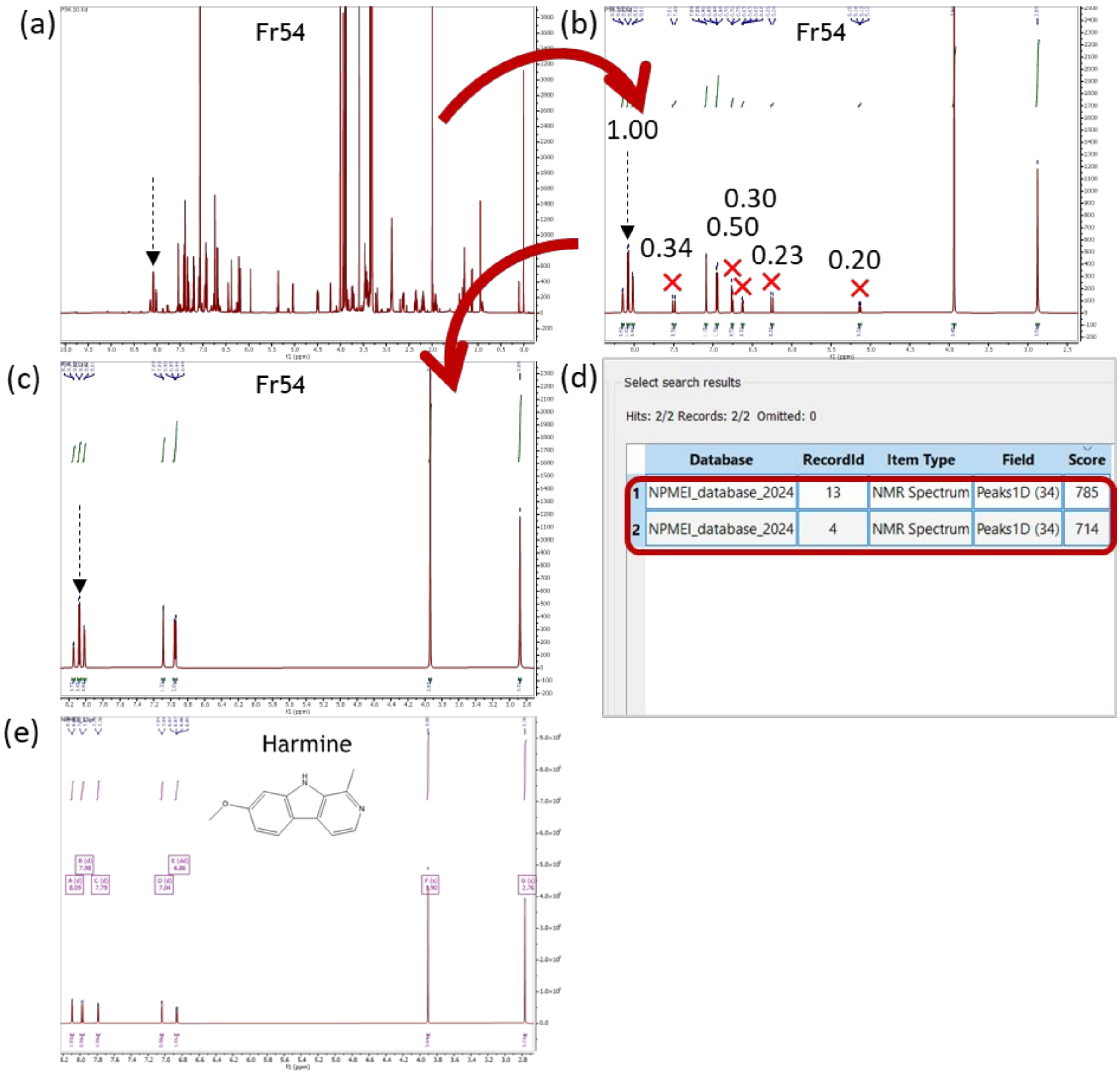

Figure S29. (a) ^{1}H NMR spectrum of the fraction Fr54 (10.00-0.00 ppm); (b) selection of STOCSY resulting peaks and spectral depletion (8.20-2.50 ppm); (c) remaining peaks (8.20-2.80 ppm); (d) NMR library results and (e) ^{1}H NMR spectrum region of the standard harmine (8.20-2.80 ppm).

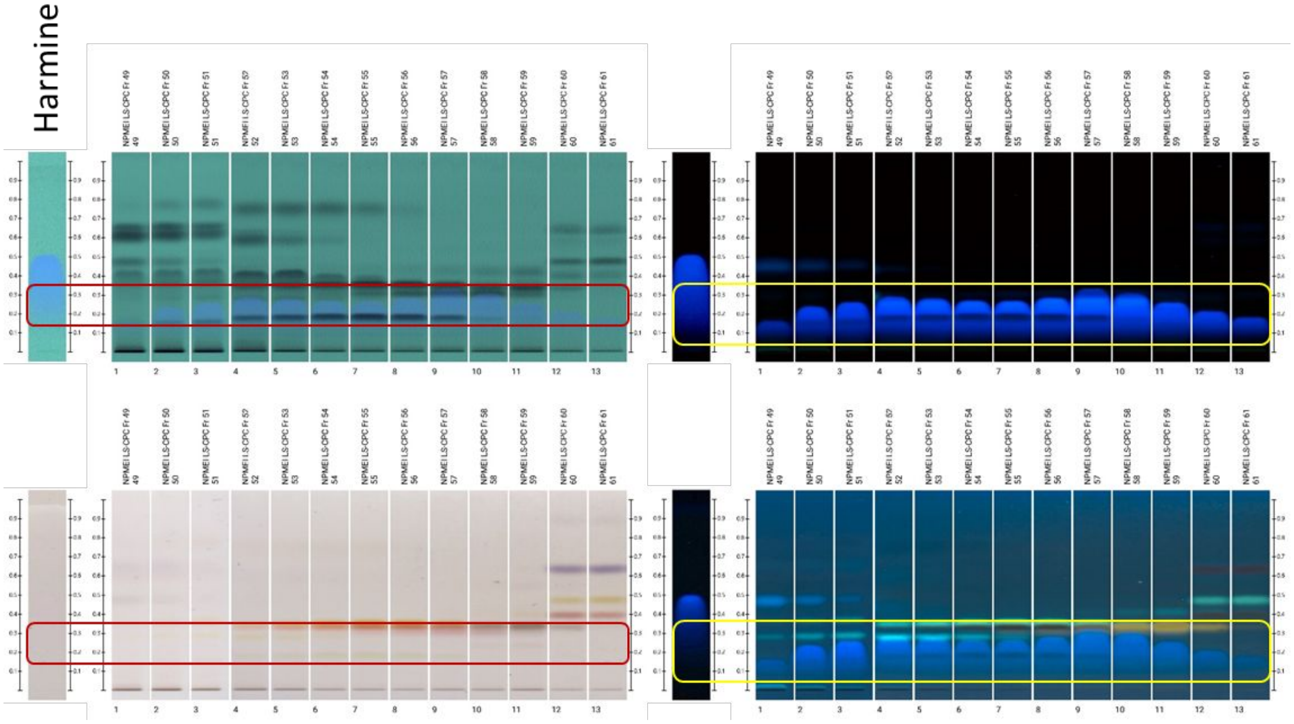

Figure S30. Comparison of the chromatograms of standard harmine with the chromatograms of the ArtExtr fractions Fr49-61 in RP. The order of the chromatograms is 254 nm, 366 nm, visible light after derivatization with SVR and 366 nm after derivatization.

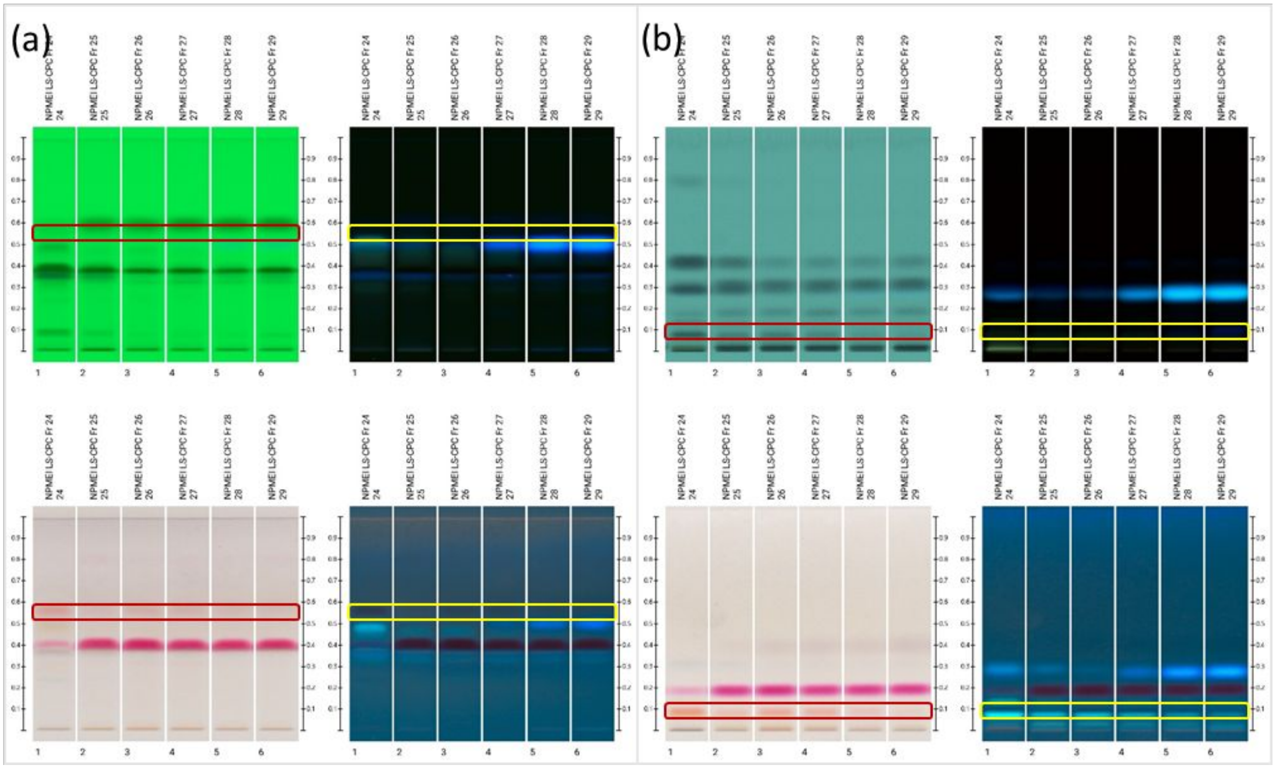

Figure S31. Chromatograms of ArtExtr fractions Fr24-29, where the HPTLC-M spot is highlighted (a) in NP and (b) in RP. The order of the chromatograms is 254 nm, 366 nm, visible after derivatization with SVR and 366 nm after derivatization.

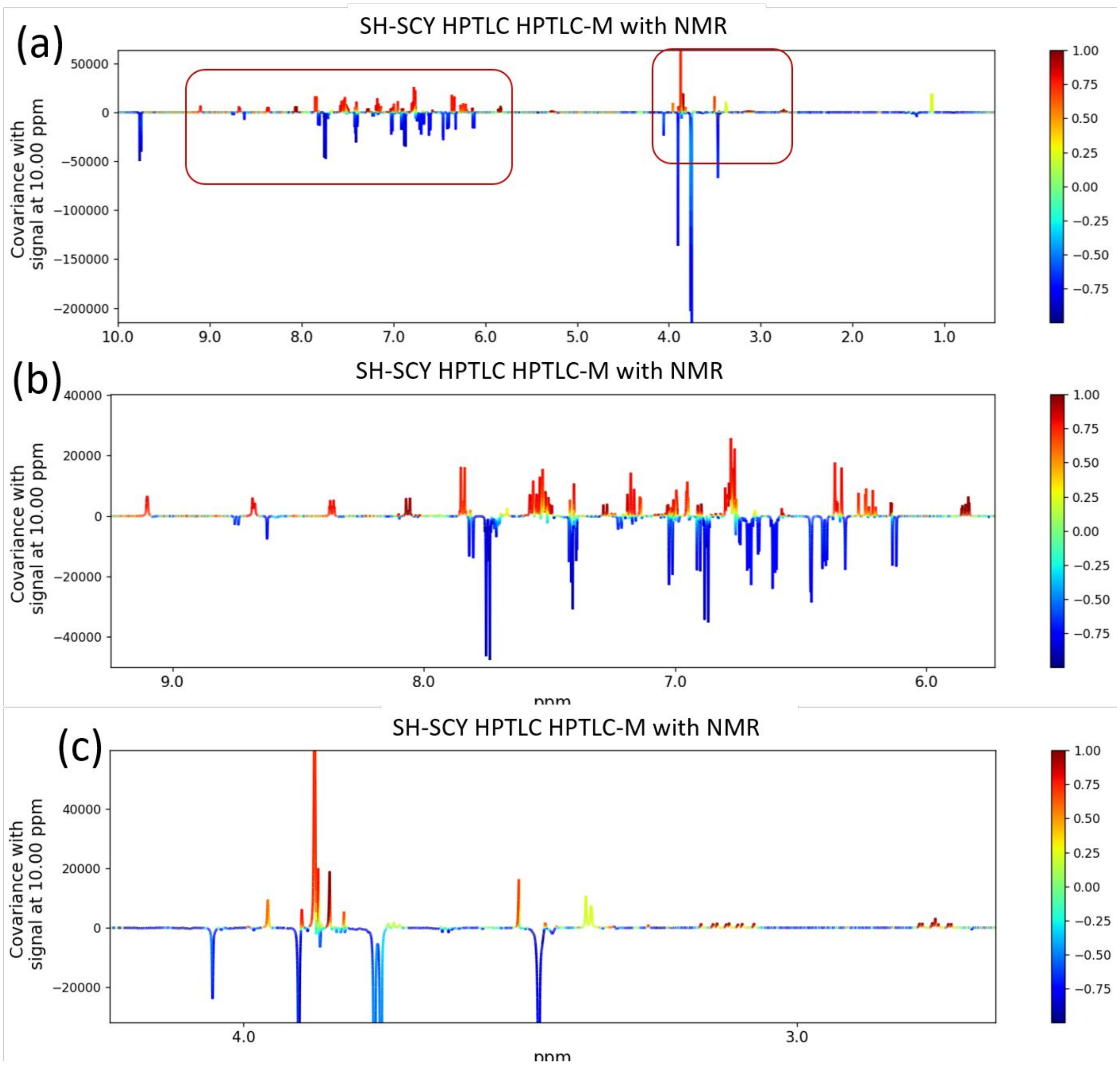

Figure S32. (a) SH-SCY pseudospectrum between HPTLC and NMR in the ArtExtr fractions Fr24-29 (10.00-0.80 ppm) for the spot HPTLC-M and zoomed areas (b: 9.20-5.80 and c: 4.20-2.60 ppm).

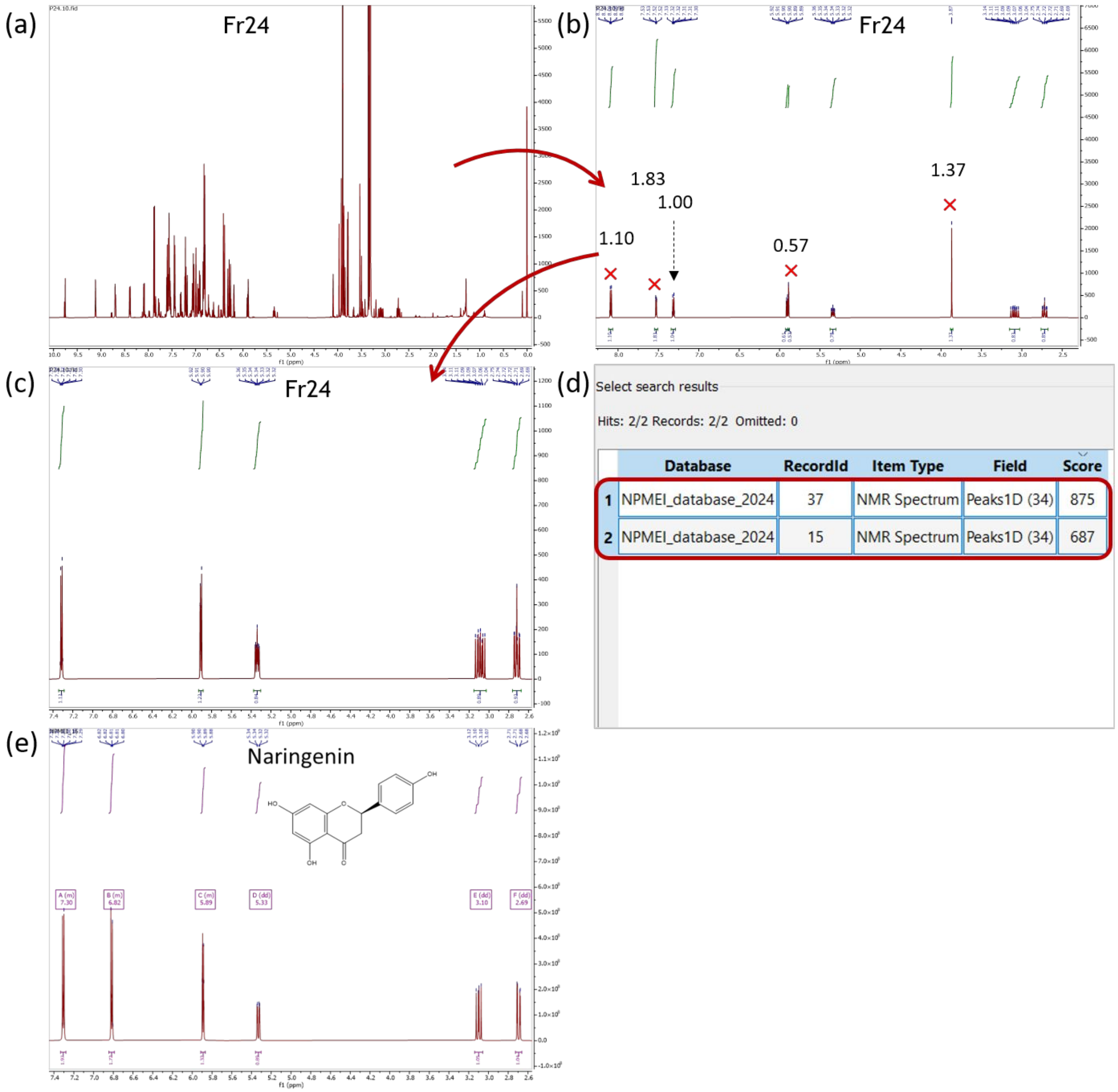

Figure S33. (a) ^{1}H NMR spectrum of the fraction Fr24 (10.00-0.00 ppm); (b) selection of STOCSY resulting peaks and spectral depletion (8.20-2.50 ppm); (c) remaining peaks (7.40-2.60 ppm); (d) NMR library results and (e) ^{1}H NMR spectrum region of the standard naringenin (7.40-2.60 ppm).

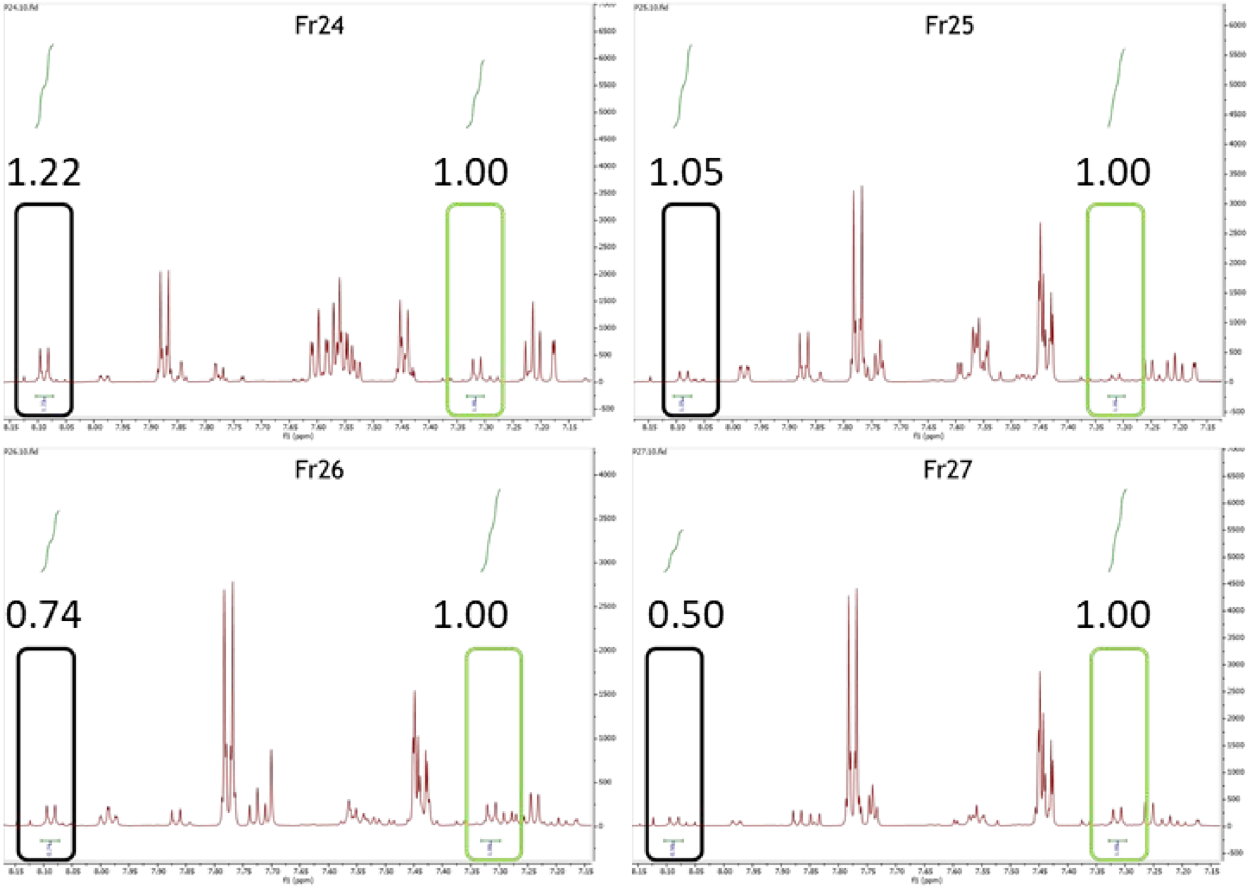

Figure S34. ^{1}H NMR spectra of ArtExtr fractions Fr24-27, in which the peaks at 8.09 ppm (black frame, excluded) and 7.31 ppm (green frame, selected) are highlighted and the inconsistency in peak integration is depicted.

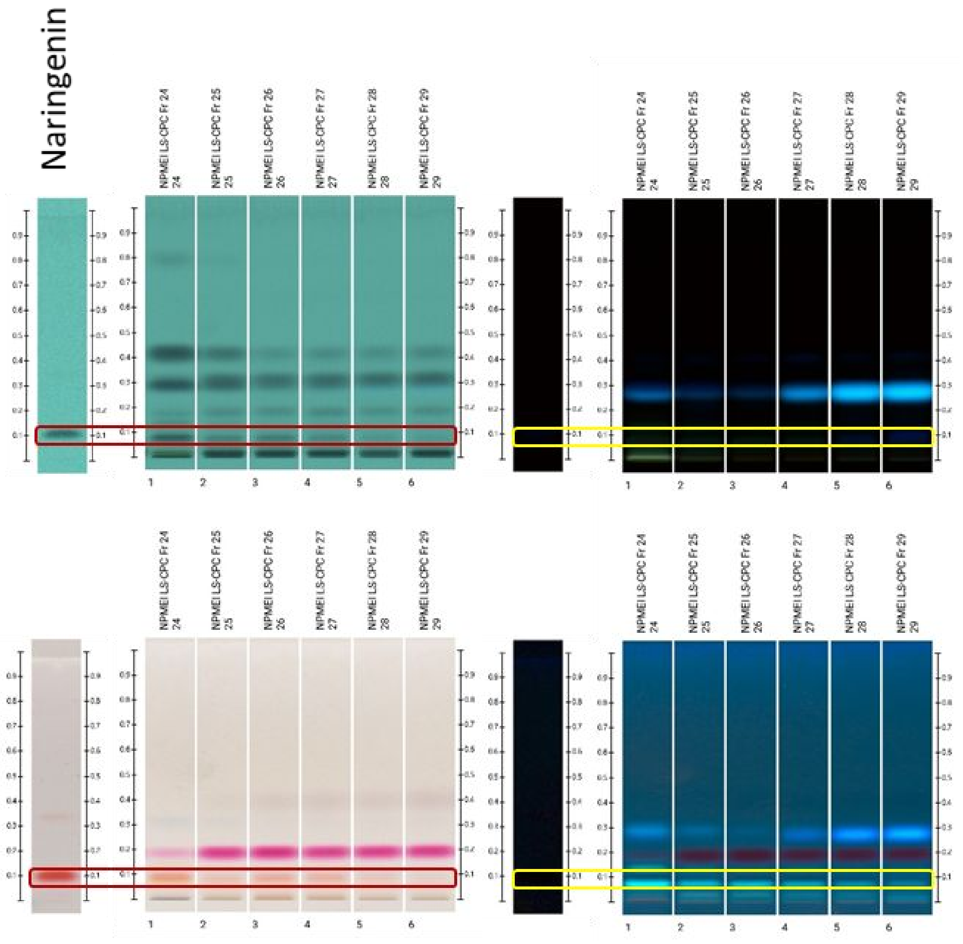

Figure S35. Comparison of the chromatograms of standard naringenin with the chromatograms of the ArtExtr fractions Fr24-29 in RP. The order of the chromatograms is 254 nm, 366 nm, visible light after derivatization with SVR and 366 nm after derivatization.

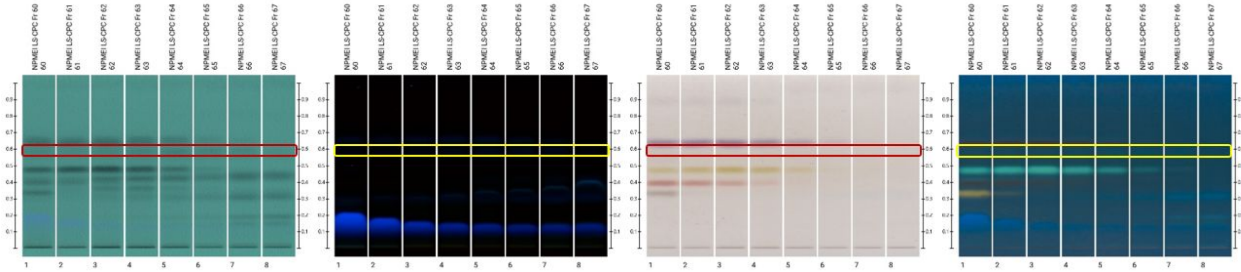

Figure S36. Chromatograms of ArtExtr fractions Fr60-67, where the HPTLC-AO spot is highlighted in RP. The order of the chromatograms is 254 nm, 366 nm, visible after derivatization with SVR and 366 nm after derivatization.

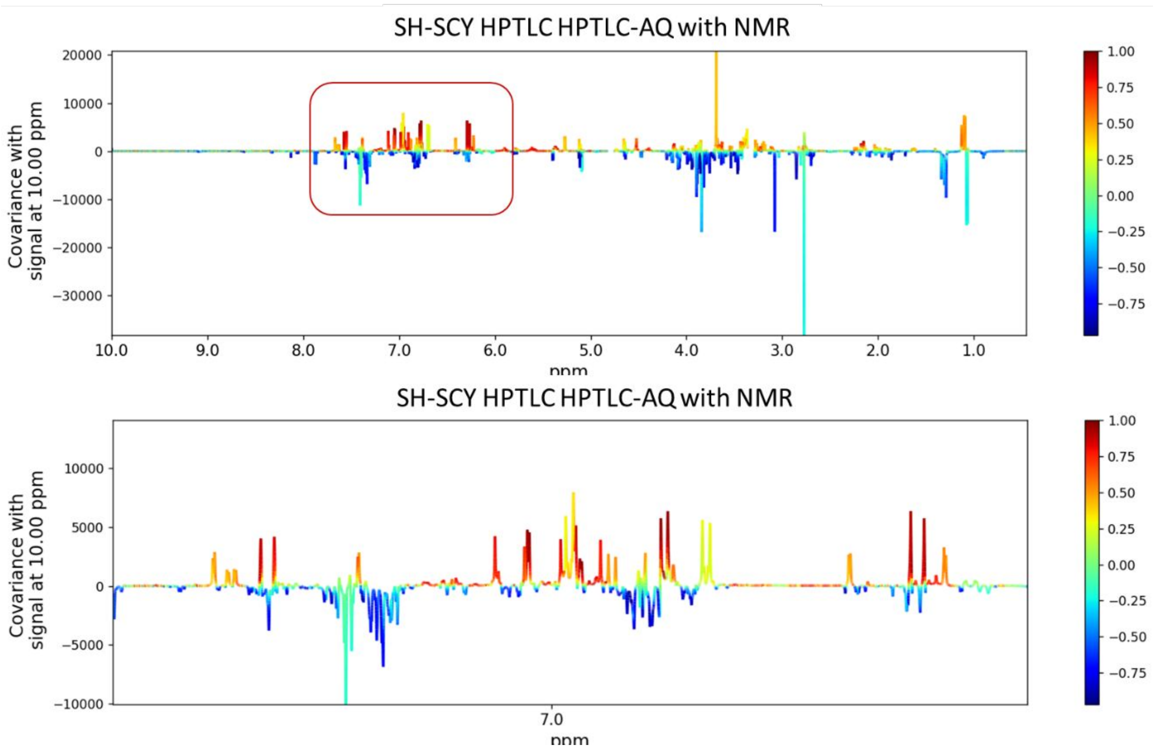

Figure S37. (a) SH-SCY pseudospectrum between HPTLC and NMR in the ArtExtr fractions Fr60-67 (10.00-0.80 ppm) for the spot HPTLC-AO and (b) zoomed area (7.80-6.00 ppm).

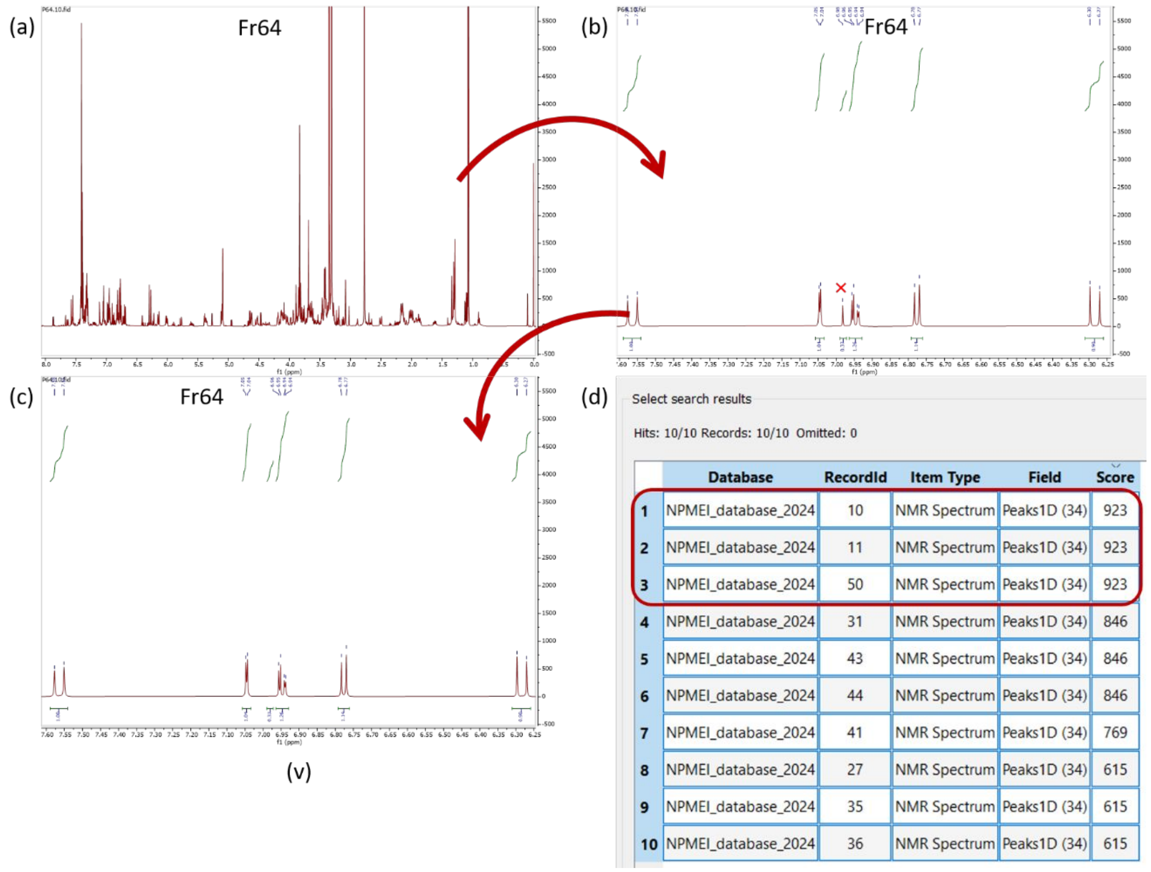

Figure S38. (a) ^{1}H NMR spectrum of the fraction Fr64 (10.00-0.00 ppm); (b) selection of STOCSY resulting peaks and spectral depletion (7.60-6.25 ppm); (c) remaining peaks (7.60-6.25 ppm) and (d) NMR library results.

Table S9. Identification of HPTLC-sHetCA resulting spots via SH-SCY with NMR.

HPTLC code	Identified compound	Score (/1000)
HPTLC-G	Aristolochic acid	909
HPTLC-I	Curcumin	1000
HPTLC-M	Naringenin	687
HPTLC-N	Umbelliferone	1000
HPTLC-O	Catechol	1000
HPTLC- P	Caffeine	1000
HPTLC- Q	Nicotinic acid	1000
HPTLC- V	-	
HPTLC- W	-	
HPTLC-X	Quercetin	1000
HPTLC-AC	-	
HPTLC-AE	-	
HPTLC-AJ	Harmine	785
HPTLC-AP	Loganin	1000
**HPTLC code**	**Partially identified compound**	**Score (/1000)**
HPTLC-AQ	Cinnamic acid derivative	

Table S10. Summary table of results from the application of the "PLANTA" protocol for the detection of bioactive compounds against DPPH in the ArtExtr.

Name	Driver ^1H NMR peak	HPTLC spot code	DPPH activity (100 μg/mL)	IC_{50} DPPH (μM)	Result	Comment
Nicotinic acid	9.09	HPTLC-Q	0.4 ± 0.5			Rejected
Umbelliferone	7.84	HPTLC-N	0.0 ± 0.4			Rejected
Rutin	7.67	HPTLC-AO	91.6 ± 0.0	46.8	Correct	
Chlorogenic acid	7.56	HPTLC-AQ	91.7 ± 0.5	87.9	Correct	Partially identified as a caffeic acid derivative
Ellagic acid (dihydrate)	7.55		95.7 ± 0.0	20.8	Correct	
Protocatechic acid	7.42	HPTLC-AD	92.7 ± 0.3	110.6	Correct	
Curcumin	7.11	HPTLC-I	95.6 ± 0.2	80.8	Correct	
Gallic acid	7.06	HPTLC-AF	95.7 ± 0.0	30.2	Correct	
Catechol	6.75	HPTLC-O	95.9 ± 0.1	48.2	Correct	
Baicalein	6.62		95.4 ± 0.0	26.3	Correct	

3,5-dihydroxybenzoic acid	6.46		1.0 ± 0.3			Rejected
Resveratrol	6.45	HPTLC-AB	74.5 ± 0.7	225.4	Correct	
Quercetin	6.39	HPTLC-X	96.6 ± 0.1	30.3	Correct	
Quercitrin	6.38	HPTLC-AH	91.4 ± 0.2	50.5	Correct	Partially identified as a quercetin-type flavonoid glycoside
Sinapic acid	6.33		93.5 ± 0.1	82.2	Correct	
Caffeic acid	6.20	HPTLC-AR	95.8 ± 0.1	44.7	Correct	
6,7-Dihydroxycoumarin (Esculetin)	6.17	HPTLC-Y	95.2 ± 0.0	27.8	Correct	
Oleuropein	6.09	HPTLC-AL	96.2 ± 0.0	61.1	Correct	
Phlorizin	5.96	HPTLC-AI	0.0 ± 0.6			Not identified
Rosmarinic acid	5.14	HPTLC-AK	95.8 ± 0.0	37.7	Correct	
Colchicine	4.50	HPTLC-AG	0.0 ± 0.3			Not identified
Aristolochic acid	4.08	HPTLC-G	0.0 ± 1.1			Rejected
Caffeine	3.97	HPTLC-P	0.0 ± 0.8			Rejected
Naringenin	3.09	HPTLC-M	0.0 ± 0.4			Rejected

Harmine	2.89	HPTLC-AJ	0.0 ± 0.8			Rejected
Loganin	1.09	HPTLC-AP	0.0 ± 0.5			Rejected
Reserpine		HPTLC-AC	0.0 ± 1.0		False positive	Not identified
Ephedrine		HPTLC-AE	0.0 ± 0.4		False positive	Not identified
Ferulic acid		HPTLC-V	90.2 ± 0.3	95.2	Correct	Not identified
Vanillic acid		HPTLC-W	6.1 ± 0.1		False positive	Not identified

Table S11. Percentages of actives, false positive and false negative results predicted by the application of the PLANTA protocol for the identification of bioactive compounds against DPPH in the ArtExtr.

DPPH	PLANTA Protocol
Compounds included in the study	55
Active compounds included in the study	19
Correctly predicted active compounds	17
Correctly predicted active compounds (%)	89.5
Non-active compounds detected through HetCA studies	32
False positive results	5

False negative results	0

ADDITIONAL REFERENCES

(1) Lee, S. E.; Hwang, H. J.; Ha, J.-S.; Jeong, H.-S.; Kim, J. H. Screening of Medicinal Plant Extracts for Antioxidant Activity. *Life Sci.* **2003**, *73* (2), 167–179. https://doi.org/10.1016/S0024-3205(03)00259-5.

(2) Wojdyr, M. *Fityk%*: A General-Purpose Peak Fitting Program. *J Appl Crystallogr* **2010**, *43* (5), 1126–1128. https://doi.org/10.1107/S0021889810030499.

(3) Zhang, Q.; Yang, W.; Liu, J.; Liu, H.; Lv, Z.; Zhang, C.; Chen, D.; Jiao, Z. Identification of Six Flavonoids as Novel Cellular Antioxidants and Their Structure-Activity Relationship. *Oxid Med Cell Longev* **2020**, *2020*, 1–12. https://doi.org/10.1155/2020/4150897.

(4) Lawag, I. L.; Sostaric, T.; Lim, L. Y.; Hammer, K.; Locher, C. The Development and Application of a HPTLC-Derived Database for the Identification of Phenolics in Honey. *Molecules* **2022**, *27* (19), 6651. https://doi.org/10.3390/molecules27196651.

(5) Schripsema, J.; Verpoorte, R.; Svendsen, A. B. Trifluoroacetic Acid, A ^{1}H-NMR Shift Reagent for Alkaloids. *Tetrahedron Lett.* **1986**, *27* (22), 2523–2526. https://doi.org/10.1016/S0040-4039(00)84574-8.
